# Metronidazole Cocrystal Polymorphs with Gallic and Gentisic Acid Accessed through Slurry, Atomization Techniques, and Thermal Methods

**DOI:** 10.1021/acs.cgd.3c00951

**Published:** 2023-10-12

**Authors:** Aleksandra J. Dyba, Ewa Wiącek, Maciej Nowak, Jan Janczak, Karol P. Nartowski, Doris E. Braun

**Affiliations:** † Institute of Pharmacy, University of Innsbruck, Innrain 52c, 6020 Innsbruck, Austria; ‡ Department of Drug Form Technology, Wroclaw Medical University, Borowska 211A, 50-556 Wroclaw, Poland; § Institute of Low Temperature and Structure Research, Polish Academy of Sciences, P.O. Box 1410, Okolna 2, 50-950 Wroclaw, Poland; ∥ School of Pharmacy, University of East Anglia, Norwich Research Park, NR4 7TJ Norwich, U.K.

## Abstract

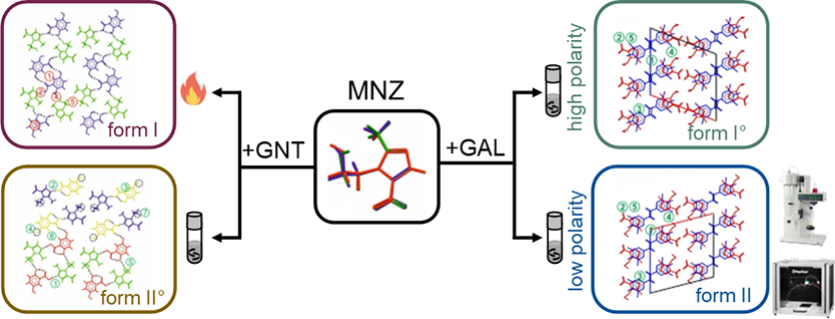

In this study, key features of metronidazole (MNZ) cocrystal polymorphs with gallic acid (GAL) and gentisic acid (GNT) were elucidated. Solvent-mediated phase transformation experiments in 30 solvents with varying properties were employed to control the polymorphic behavior of the MNZ cocrystal with GAL. Solvents with relative polarity (RP) values above 0.35 led to cocrystal I°, the thermodynamically stable form. Conversely, solvents with RP values below 0.35 produced cocrystal II, which was found to be only 0.3 kJ mol^–1^ less stable in enthalpy. The feasibility of electrospraying, including solvent properties and process conditions required, and spray drying techniques to control cocrystal polymorphism was also investigated, and these techniques were found to facilitate exclusive formation of the metastable MNZ-GAL cocrystal II. Additionally, the screening approach resulted in a new, high-temperature polymorph I of the MNZ-GNT cocrystal system, which is enantiotropically related to the already known form II°. The intermolecular energy calculations, as well as the 2D similarity between the MNZ-GAL polymorphs and the 3D similarity between MNZ-GNT polymorphs, rationalized the observed transition behaviors. Furthermore, the evaluation of virtual cocrystal screening techniques identified molecular electrostatic potential calculations as a supportive tool for coformer selection.

## Abstract 2

The cocrystallization potential of metronidazole (MNZ) using various experimental and computational methods is explored. A new, high-temperature polymorph of MNZ-gentisic acid is identified, which is enantiotropically related to the already described form II°. Solvent polarity influences the crystallization kinetics and determines whether MNZ-gallic acid cocrystallize into form I° or II, while electrospraying and spray drying exclusively yield the metastable form II.

## 1 Introduction

Pharmaceutical cocrystals are widely recognized as an effective strategy for improving the physicochemical properties of active pharmaceutical ingredients (APIs). These properties include dissolution profile, bioavailability, stability, compressibility, and so forth.^1−3^ However, cocrystals, just like their components, are also prone to exhibit polymorphism, and several polymorphic forms have been reported for some cocrystal systems.^4,5^ Moreover, the outcome of cocrystallization is not entirely predictable as many coformer pairs do not generate cocrystals despite their theoretical compatibility, which is often assessed prior to the experimental trials. Therefore, the selection of coformers, coupled with careful tuning of the crystallization conditions, is of great importance to the cocrystal screening process and control of the polymorphic outcome of cocrystallization reactions.

During solvent-based crystallization experiments, either for single components or potential cocrystal API-coformer pairs, the interactions between the dissolved components and the solvent molecules play a significant role in nucleation, crystal growth, solvate formation, and transformation to other polymorphic forms.^6−9^ Therefore, the solvent selection process must be a focal point for solvent-based cocrystallization, taking into consideration not only the properties of the API but also those of the coformers, such as their solubility and possible interactions with solvent molecules.^10−12^ Many essential parameters have been previously described for solvent-based (co)crystallizations, including the hydrogen bond acceptance ability of the solvent (δ_hAcc_)^13^ or its polarity,^14^ as well as the effect of stirring and temperature conditions,^15^ cooling rate (if applicable), and seeding.^16^ Furthermore, Eddleston et al. have emphasized the need for a multitechnique approach when screening for cocrystals.^4^

**Scheme 1:**
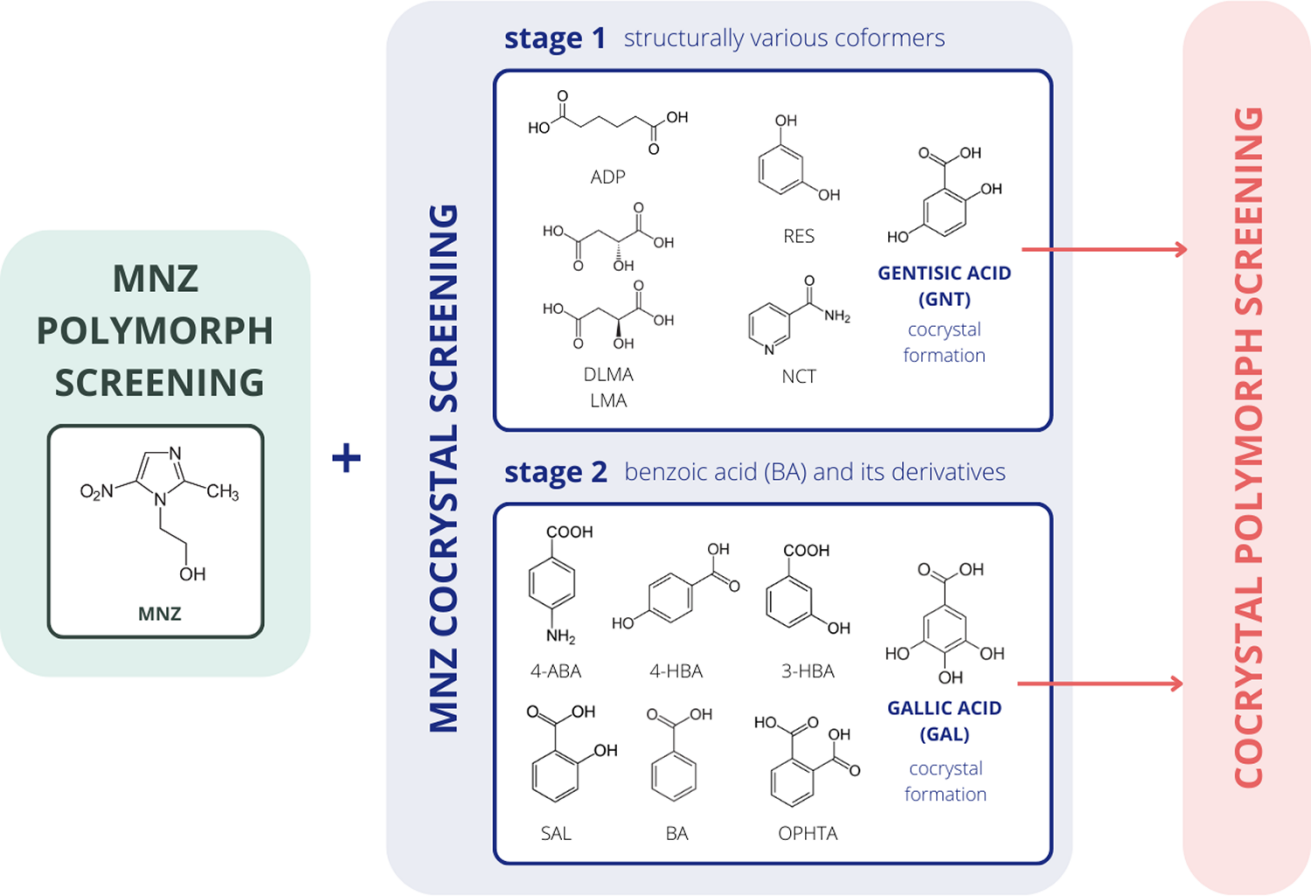
Molecular Diagrams of MNZ and Coformers Used in This Study for Polymorphism and Cocrystal Screening

In this work, we focused on metronidazole [2-(2-methyl-5-nitro-1H-imidazol-1-yl)ethanol, MNZ, Scheme 1], a BCS class I compound from the World Health Organization’s List of Essential Medicines used for the treatment of protozoan and anaerobic infections.^17,18^ MNZ is available on the market in many forms, *e. g.* tablet, oral liquid, suppository, or injection. In its pure form, MNZ exhibits a water solubility of 10 mg mL^–1^ at 20 °C.^19^ To date, two different sets of lattice parameters have been reported for MNZ (CSD-Refcode family MNIMET). The first one, namely MNIMET and MNIMET02–06, is a *P*2_1_/*c* cell,^20−25^ whereas for MNIMET01, the *P*11 2_1_/*n* space group has been assigned.^26^ Transforming the MNIMET01 cell to the standard setting indicates the presence of different lattice parameters. A discrepancy in the formula weight has been noted, suggesting that the given cell parameters may correspond to a different compound and not MNZ. The latter is further supported by the fact that MNIMET01 would be approximately 30% denser than the MNIMET, MNIMET03, and MNIMET06 structures (all determined at RT). Therefore, so far, it may be assumed that MNZ is monomorphic.

Only a few MNZ cocrystals have been reported so far, including cocrystals with gallic acid (GAL) (VOKYEC^27^ and VOKYEC01^28^), pyrogallol (KUDRUZ),^29^ ethyl gallate (ABITAK),^19^ and 3,5-dihydroxybenzoic acid cocrystal hydrate (JUMQAM).^28^ Although the MNZ cocrystal structure with gentisic acid (GNT) was included in the US 2009/0258869 A1 patent,^30^ atomic positions were not disclosed and the structure was not made publicly available. Both polymorphs of the MNZ 1:1 cocrystal with GAL have already been deposited in the CSD,^31^ although no systematic polymorphism control study was performed. Form I° (VOKYEC) was produced by the dropwise addition of an aqueous GAL solution into a stirred aqueous MNZ solution. The cocrystal was found to exhibit lower *c*_max_ and longer *T*_max_ as compared with neat MNZ.^27^ Form II (VOKYEC01) was obtained using thermal inkjet printing using a water/EtOH solution as the organic ink. The MNZ-GAL cocrystal is also mentioned in the US 2009/0258859 A1 patent. The peaks of the recorded diffractogram can be assigned mainly to form II of the cocrystal.^30^ Furthermore, the polymorphism of MNZ benzoate ionic cocrystals with salicylic and fumaric acid as coformers has been reported very recently.^32^

Electrospraying (ES) and spray drying (SD) are emerging techniques for cocrystal and polymorph screening.^33−36^ SD is a particle processing technique that is already well established in the pharmaceutical industry and allows for modification of particle properties, for example, to aid with suitable dispersibility for pulmonary drug delivery.^37^ Both methods involve dispersing a liquid into fine droplets that dry within the processing time, and the main factor promoting nucleation and crystal growth is the rapid concentration increase. Spray-dried particles are dried by coming into contact with a hot gas stream, while ES utilizes an electric field to charge the drying particles and prevent their aggregation.^38,39^ As electrospraying does not require the high temperature of SD, it is suitable for compounds that are thermolabile.^40^ Crystallization using ES has been demonstrated by Radacsi et al. as a way to obtain nanosized crystals of the anti-inflammatory niflumic acid^41^ and an energetic material, cyclotrimethylene trinitramine.^42^ ES has also been investigated in the context of polymorphism control of the model compound carbamazepine.^35^ However, few investigations of cocrystallization by means of ES have been reported. Patil et al. explored several systems like caffeine/maleic acid, carbamazepine/nicotinamide, and itraconazole cocrystals with fumaric and succinic acids.^34,43^ An insight into processing congruently and incongruently saturating systems using ES deposition has been published, where ES proved more efficient than solvent evaporation, considering the incongruent conditions of the experiment.^44^ ES could potentially be applied for other incongruently saturating systems, which pose a significant challenge for standard techniques, although studies on cocrystallization using this method are limited thus far.

The selection of suitable coformers is a crucial step for cocrystal design and often involves multiple unsuccessful experimental attempts due to the vast array of available options. To streamline the process and reduce costs and time, several computational methods have been developed to predict the likelihood of cocrystal formation for a given API. One such method is the molecular complementarity (MC) approach developed by Fabian, which utilizes a set of molecular descriptors such as the dimensions of a rectangular box around the molecule, the dipole moment, and the fraction of nitrogen/oxygen atoms.^45^ Another widely used virtual cocrystal screening method is the multicomponent hydrogen bond propensity tool (MCHBP),^46^ which predicts the likelihood of hydrogen bond formation. Additionally, Musumeci et al. have demonstrated the usefulness of molecular electrostatic potential (MEP) calculations in identifying complementary H-bond donor and acceptor site pairs ranked by their strength.^47^ In this work, all three methods were applied to the compounds of interest, and the results were compared with experimental data.

The aim of this study was to investigate MNZ cocrystal formation (Scheme 1) using the stirred suspension technique and computational methods, such as MC, MCHBP, and MEP. Additionally, we aimed to conduct polymorphism screenings for MNZ-GAL and MNZ-GNT, investigating a range of solvents to control the outcomes of polymorphism. We also examined the feasibility of ES, SD, and freeze-drying cocrystallization in selected solvents for either MNZ-GNT or MNZ-GAL systems. The study further addresses the important parameters, including solvent and reagent properties, as well as instrument settings required for these processes. Furthermore, we aimed to gain insights into the thermodynamic relationship between the polymorphic cocrystal forms by using differential scanning and isothermal calorimetries and to understand the influence of solvents on transformation kinetics in the MNZ-GAL cocrystal system.

## 2 Experimental Section

### 2.1 Materials

MNZ (99.0–101.0%), urea (UREA, ≥ 99.0%), L-malic acid (LMA, ≥ 99.0%) form I,^48^ DL-malic acid (DLMA, ≥ 99.0%), benzoic acid (BA, 99.0–101.0%), salicylic acid (SAL, 99.0–101.0%), GNT (≥ 99.0%) form II°,^49^ GAL (99.0–101.0%) form II,^50^ nicotinamide (NCT, ≥ 99.5%) form α,^51^ 4-aminobenzoic acid (4-ABA, 99.0–101.0%), 4-hydroxybenzoic acid (4-HBA, ≥ 99.0%), D-limonene (D-LIM, ≥ 95.0%), tetrahydrofuran (THF, ≥ 99.5%), *N*-methyl-2-pyrrolidon (NMP, ≥ 99.5%), 1,3-dimethyl-1,3-diazinan-2-one (DMPU, ≥ 97.0%), *n*-buthylmethyl ether (BuMetET, ≥ 99.0%), diisopropylether (isPrET, ≥ 99.0%), 4-methyl-2-pentanone (methyl isobutyl ketone, MIBK, ≥ 98.0%), 1,2-dichloroethane (DCE, ≥ 99.5%), and butyl acetate (BuAc, ≥ 98.0%) were purchased from POL-AURA. *O*-phthalic acid (OPHTA, ≥ 99.5%), 3-hydroxybenzoic acid (3-HBA, 99.0%) form I,^52^*n*-butanol (*n*-BuOH, 99.8%), *tert*-butanol (*t*-BuOH, ≥ 99.0%), *iso*-propanol (*i*-PrOH, ≥ 99.5%), *n*-propanol (*n*-PrOH, ≥ 99.7%), ethyl acetate (EtAc, ≥ 99.5%), dichloromethane (DCM, ≥ 99.5%), chloroform (Clf, ≥ 99.0%), dimethyl isosorbide (isSORB, ≥ 99.0%), 2-phenoxypropanol (phenPrOH, ≥ 93.0%), and α,α,α-trifluorotoluene (BTF, ≥ 99.0%) were purchased from Sigma-Aldrich. Adipic acid (ADP, 99.8–99.9%) form I,^53^ resorcinol (RES, ≥ 99.0%) form α,^54^ toluene (TOL, ≥ 98.0%), *n*-hexane (HEX, ≥ 99.0%), and diethyl ether (ETDE, ≥ 99.5%) were purchased from CHEMPUR. Methanol (MeOH, ≥ 99.0%) and ethanol (EtOH, ≥ 99.0%) were purchased from Honeywell. Acetic acid (AcCOOH, ≥ 96.0%) and acetonitrile (ACN, ≥ 99.9%) were purchased from Witko. Acetone (ACT, 99.5%) was purchased from J. T. Baker. 2-pentanone (2-PENT, ≥ 98.0%) was purchased from Acros Organics. *Iso*-butanol (*i*-BuOH, ≥ 99.0%) was purchased from Fluka Chemie AG. All chemicals were used without further purification. Care was taken to keep the water content of the solvent as low as possible under adequate storage conditions.

### 2.2 Preparative Methods

Throughout this work, RT (room temperature) corresponds to a value of 24 ± 2 °C.

#### 2.2.1 MNZ Polymorph Screening Procedures

Stirred suspension crystallization and slow solvent evaporation experiments, as detailed in ESI Table S1, were employed. The powder X-ray diffraction (PXRD) and Fourier transform infrared (FTIR) data collected during the polymorphism screening are presented in ESI Figures S1 and S2.

#### 2.2.2 MNZ Cocrystal Screening Procedures

The MNZ cocrystal screening process included two stages (Scheme 1). In the first stage, a structurally diverse set of coformers was used. All stage 1 coformers are Generally Recognized as Safe compounds which exhibit diversity regarding their structural features (hydrogen bond donor/acceptor groups and aromatic/nonaromatic groups) and physicochemical properties (p*K*_a_, melting temperature, and log *P*). They are known to form cocrystals with other pharmaceutically relevant compounds featuring functional groups related to MNZ.^55,56^ Only experiments employing GNT, a BA derivative, yielded a cocrystal with MNZ during stage 1 of the screening process. Therefore, BA and selected derivatives (focusing on known cocrystal-forming compounds) were chosen for stage 2 of the MNZ cocrystal screening.

For both stages of the MNZ slurry cocrystal screening, MNZ and the coformer were separately ground and then weighed in a 1:1 molar ratio and transferred into capped glass vials. The resulting powder was then suspended in the solvent of choice and stirred for 7 days at 300 rpm using a magnetic stirrer at RT. Afterward, the product was filtered, dried, and subjected to structural and thermal analyses. For additional information and PXRD and FTIR data, please refer to Tables S2 and S3 and Figures S3, S4, and S6–S15 in the ESI.

For the MNZ-NCT system, additional slow evaporation experiments were performed. Briefly, 100 mg of an equimolar physical mixture of MNZ and NCT was weighed and transferred into a glass vial, followed by solvent addition and stirring at RT until both components dissolved. The solution was then left to crystallize, and the collected powder was subjected to further analysis. For additional information and PXRD and FTIR data, please refer to Table S2 and Figure S5 in the ESI.

For MNZ and selected coformers (3-HBA, 4-ABA, 4-HBA, and BA), additional freeze-drying experiments were performed. Briefly, 50 mg of 1:1 physical mixtures were dissolved in 10 mL of H_2_O and frozen using liquid nitrogen and then freeze-dried using the Lyovac GT2 freeze drier (SRK Systemtechnik GmbH, Germany) with a drying time of 19 h. For additional information, PXRD and FTIR data refer to ESI Table S3 and Figures S10–S13.

#### 2.2.3 MNZ-GAL Polymorph Cocrystal Screening

##### 2.2.3.1 Stirred Suspension Crystallization (Slurry Experiments)

Equimolar mixtures containing 150.5 mg of MNZ and 149.5 mg of GAL were prepared and transferred to capped glass vials. The mixtures were then suspended in the solvents (200 μL) and stirred for 7 days at 300 rpm using a magnetic stirrer at RT. If a phase pure product was not obtained from the experiment, the physical mixture was stirred in a saturated solution of both reagents for 2 weeks at 500 rpm with temperature cycling between 10 and 30 °C. The saturated solutions of the reagents were prepared by suspending an excess of both components in a solvent and stirring the mixture for 24 h at RT. The suspension was then filtered, and the resulting liquid was used in the experiments. The use of saturated solutions was necessary due to solubility differences between MNZ and GAL.

##### 2.2.3.2 Cocrystallization Using ES

Equimolar solutions of MNZ-GAL were electrosprayed using a Spraybase Kit (Spraybase, UK). The solutions were fed by using a pump and a 10 mL syringe with a stainless steel 22G needle (0.413 mm inner diameter) as the emitter, which was connected to a syringe (Ossila Syringe Pump Single/Dual, L2003D-0055, Ossila, UK) via a PTFE tube. The solutions were sprayed onto a grounded metal collector plate, and the particles were stored in a desiccator for further analysis. Various needle-to-collector working distances (4/6/8/10/15 cm), feed rates (0.5/1.0/1.5/2.0 μL s^–1^), and applied voltages (in the range of 10–22 kV) were tested to determine the optimum ES conditions. In the case of water, a lower feed rate of 0.019 μL of s^–1^ was necessary. All experiments were carried out at RT. The optimal parameters for each experiment are denoted in ESI Table S4.

##### 2.2.3.3 Cocrystallization Using SD

Equimolar solutions of MNZ-GAL were spray-dried using Mini Spray Drier B-290 (Buchi, Flawil, Switzerland). A 0.7 mm two-fluid nozzle was used as the emitter in combination with the Inert Loop B-295 and/or the 296 Dehumidifier (Buchi, Flawil, Switzerland). The aqueous sample was processed in the open mode, while all other solutions were dried in the closed mode with nitrogen as the drying medium. The resulting dried product was collected and stored in a desiccator until structural and thermal analyses. The processing parameters were adjusted to account for the varying properties of the solvents, and all conditions are listed in ESI Table S5.

#### 2.2.4 MNZ-GNT Polymorph Cocrystal Screening

##### 2.2.4.1 Stirred Suspension Crystallization (Slurry Experiments)

Please refer to Section 2.2.2 for the methodology and Table S2 of the ESI for the list of experiments.

##### 2.2.4.2 Cocrystallization Using ES, SD, and Freeze-Drying

To electrospray the equimolar MNZ-GNT aqueous solution (50 mg of 1:1 physical mixture in 10 mL H_2_O), the equipment described in the previous section was used. The emitter was a 22G stainless steel injection needle, the working distance was 15 cm with a 0.019 μL s^–1^ feed rate, and the applied voltage was 20–22 kV. The SD experiment was conducted using the Mini Spray Drier B-290 (Buchi, Flawil, Switzerland) with a 0.7 mm two-fluid nozzle in the open mode under the compressed air flow rate of 414 L h^–1^ and 500 mg of a 1:1 physical mixture in 100 mL H_2_O was used for the experiment. The inlet and outlet temperatures were 100 and 60–62 °C, respectively. The feeding rate was 3 mL min^–1^ and the aspirator was set to 100%. The freeze-drying experiment was carried out using the Lyovac GT2 freeze drier (SRK Systemtechnik GmbH, Germany), with 50 mg of a 1:1 physical mixture in 10 mL of H_2_O. The aqueous MNZ-GNT solution was first frozen using liquid nitrogen before freeze-drying, and the drying time was 19 h.

### 2.3 Analytical Methods

#### 2.3.1 PXRD

The PXRD patterns were collected using a D2 PHASER diffractometer (Bruker AXS, Karlsruhe, Germany) with Cu Kα (1.5418 Å) radiation in the 2θ range of 5–36°. A step size of 0.02° was used with a 1.0 s irradiation time per step. A 2.5° Soller slit module system was used with a 0.2 mm divergence slit as well as a 1 mm air-scatter screen and a Ni filter. The operating conditions for the X-ray tube were 30 kV and 10 mA. Samples were ground by using an agate pestle and a mortar prior to analysis.

#### 2.3.2 Temperature-Controlled PXRD and Structure Solution of MNZ-GNT Form I from Powder Pattern

PXRD heating experiments were performed using an X’Pert PRO diffractometer (PANalytical, Almelo, NL) in transmission geometry in the 2θ range from 2 to 40° (70°), a 2θ step size of 0.013°, and 40 or 400 s per step. A Cu–K_α1,2_ radiation source was used with a PIXcel1D detector and the settings of 40 kV/40 mA. A Bruker heatable accessory holder was used as the heating device, and the measurements were performed on an Al foil.

The MNZ-GNT form I diffraction pattern was indexed to a triclinic unit cell using the first 20 peaks with DICVOL, and the space group was determined to be *P*-1.^57,58^ From the cell volume, it was derived that there is one MNZ and one GNT molecule in the asymmetric unit. The data were background subtracted, and Pawley refinement^59^ was used to extract the intensities and their correlations. Simulated annealing was used to optimize the model against the diffraction data set in direct space. The internal coordinate (Ζ-matrix) description was derived from the PBE0/6-31G(d,p) gas-phase global conformational minima, with O–H distances normalized to 0.9 Å and the C–H distances normalized to 0.95 Å. The structure was solved using 100 simulated annealing runs of 2 × 10^8^ moves per run as implemented in DASH, allowing 12 external and 4 internal degrees of freedom. The best solution returned a χ^2^ ratio of ca. 3.4 (profile χ^2^/ pawley χ^2^). Rigid body Rietveld refinements^60^ were performed in TOPAS V7.^61^ The rigid body description was derived from the Ζ-matrix of the PBE-MBD* optimized structure (for details, see ESI Section 4.3). Form II° impurities were still present; therefore, a mixed Rietveld refinement using the experimental form II° parameters (see next section), and the PBE-MBD* optimized form I structure was applied. The background was modeled with Chebyshev polynomials, and the modified Thompson-Cox-Hastings pseudo-Voigt (TCHZ) function was used for peak shape fitting. The final refinement refined to 6.7% of form II°, with a total of 81 parameters (20 profile, 12 cell, 1 scale, 1 isotropic temperature factor, 11 preferred orientation, and 36 rotations), and yielded a *R*_wp_ of 6.30 (ESI Table S6 and Figure S22).

#### 2.3.3 Single Crystal X-ray Diffraction

Graphite monochromatic Mo Kα radiation on a four-circle κ geometry Rigaku Xcalibur diffractometer with a two-dimensional Atlas CCD detector was used to collect X-ray intensity data for the MNZ-GNT cocrystal II°. The ω-scan technique with Δω = 1.0° for each image was employed for data collection. No correction for the relative intensity variations was performed. The CrysAlis CCD program was used to collect the data at RT (295 K) and LT (100 K).^62^ The CryAlis Red program was used for integration, reflections scaling, correction for Lorenz and polarization effects, and absorption corrections.^62^ SHELXT was used to solve the structure by direct methods.^63^ The refinement was done using the SHELXL-2018 program.^64^ The COOH H-atoms were identified from different Fourier maps, and the remaining H-atoms were included in the refinement using a riding model. For more details, see ESI Table S6 and Section 4.4.

#### 2.3.4 FTIR Spectroscopy

FTIR spectra in the attenuated total reflectance (ATR) mode were collected using a Nicolet iS50 spectrometer (Thermo Scientific, USA). Overall, 32 scans per sample over a range of 400–4000 cm^–1^ were collected with a resolution of 4 cm^–1^.

#### 2.3.5 Hot-Stage Microscopy

For hot-stage thermomicroscopic investigations, a Reichert Thermovar polarization microscope, equipped with a Kofler hot-stage (Reichert, A) was used. Heating rates in the range of 1–10 °C min^–1^ were applied. Photographs were taken with a DP71 digital camera (Olympus, A).

#### 2.3.6 Differential Scanning Calorimetry

Initially, approximately 5 ± 0.5 mg of sample were used to record differential scanning calorimetry (DSC) thermograms using a DSC 214 Polyma (Netzsch, Germany) instrument. The material was sealed in aluminum pans with pierced lids. The materials were heated in a nitrogen atmosphere (flow rate of 25 mL min^–1^) from 0 to 269 °C with a heating rate of 5 °C min^–1^. The instrument was calibrated using a set of references (In, Sn, Bi, Zn, Al, and Hg). The DSC thermograms were analyzed using Proteus Analysis software v.7.1.10 (Netzsch, Germany).

The heats of transformations were recorded using a DSC 7 (PerkinElmer Norwalk, Norwalk, CT, USA) instrument and Pyris software. Using an UM3 ultramicrobalance (Mettler, Greifensee, CH), samples of approximately 3 mg were weighed into perforated aluminum pans. The samples were heated at rates of 5 °C min^–1^ with dry nitrogen as the purge gas (purge: 20 mL min^–1^). The instrument was calibrated for temperature with pure benzophenone (Mp = 48.0 °C) and caffeine (Mp = 236.2 °C), and the energy calibration was performed with indium (heat of fusion 28.45 J g^–1^). The errors in the stated onset temperatures and enthalpy values are calculated at the 95% confidence level and are based on at least three measurements.

The thermodynamically stable forms at RT are highlighted with “°” in this study.

#### 2.3.7 Isothermal Solution Calorimetry

The experiments were performed with a TAM III nanocalorimeter unit (TA Instruments, US). Solution calorimetry data were recorded using a 20 mL micro solution ampule. The experiments were performed at 25 °C in 15 mL of DMSO. Approximately 7–12 mg of the sample were accurately weighed into reusable stainless-steel capsules using a UM3 ultramicrobalance (Mettler, CH). Once the baseline had stabilized to ±50 nW, the capsule was dropped into the calorimeter. The heat flow into or out of the calorimeter was recorded, and data analysis was performed using TAM Assistant software. The heat flow of the empty RH capsule was subtracted from the heat flow of the sample measurements. The errors on the stated enthalpy values are calculated at 95% confidence intervals and are based on at least three measurements. The calorimeter was calibrated periodically using the electrical substitution method as well as with reference materials (KCl and sucrose).

### 2.4 Virtual Cocrystal Screening

#### 2.4.1 MC

Using the CSD Materials module from Mercury 2023.1.0,^45,65^ MC calculations were performed and the results were classified as either PASS or FAIL. For MNZ and the coformers used in the experimental section of this study, as well as three additional cocrystal-forming coformers (ethyl gallate, 3,5-dihydroxybenzoic acid, and pyrogallol), the calculations were conducted using gas-phase optimized conformers [B3LYP/6-311++G(d,p)], calculated using Gaussian16.^66^ The experimentally observed conformers were also taken into account, with constrained optimizations applied (i.e., rotatable bonds fixed to experimentally observed values, while the rest of the molecule was optimized). The optimal combination of MNZ and coformer was then determined and used for the final MC results.

#### 2.4.2 MCHBP

The calculations were conducted using the Hydrogen Bond Propensities module from Mercury^65^ and the CSD Python API. The same set of coformers used in the MC calculations were employed. The propensity of the most significant heteromeric interaction between MNZ and a coformer (A–B) was compared with the highest homomeric interaction, either MNZ–MNZ (A–A) or coformer–coformer (B–B). The difference, Δ_HBP_ = *P*_A–B_ – [max(*P*_A–A_, *P*_B–B_)], was calculated.

#### 2.4.3 MEP Maps

Geometry optimization, both full and constrained, was performed using Gaussian16^66^ with the B3LYP DFT method and the 6-311++G(d,p) basis set. The set of conformers comprised the gas-phase global minima and diverse experimentally observed conformations. To map the MEPs of MNZ and the selected coformers onto their 0.002e^–^ Å^–3^ electron density isosurface, Multiwfn 3.7^67^ was employed. Different conformations were considered during this process.

Using the identified local maxima (MEP_max_) and minima (MEP_min_), the H-bond donor parameter (α) and H-bond acceptor parameter (β) were calculated as described in previous studies.^47^ To estimate the potential energy gain (Δ*E*_MEP_, in kJ mol^–1^) upon cocrystal formation, eq 1 was used:
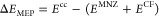
1

Either all possible combinations of MNZ and coformers, differing in their conformation and H-bond parameters (α and β) (approach 1), or the combination of the lowest *E*^MNZ^ and *E*^CF^ values (approach 2) was used to estimate Δ*E*_MEP_. CC, MNZ, and CF represent the cocrystal, metronidazole, and coformer, respectively, and the energies correspond to “interaction” energies calculated from the α and β values (for details, see ESI Section 6.3).

### 2.5 Pairwise Intermolecular Energy Calculations

CrystalExplorer V17^68^ was utilized in conjunction with the PBE-MBD* optimized structures (for calculation details see ESI Section 5) and a 3.8 Å radius to determine the pairwise intermolecular energy.^69−71^ Gaussian16 software^66^ was employed to compute B3LYP/6-31G(d,p) molecular wave functions, which were used to derive the densities of unperturbed monomers. This enabled the determination of the four distinct energy components, namely, electrostatic (*E*_E_), polarization (*E*_P_), dispersion (*E*_D_), and exchange-repulsion (*E*_R_).

## 3 Results and Discussion

### 3.1 Experimental MNZ Polymorph Screening

This study performed a polymorphism screening of MNZ using two different techniques: slurry crystallization (utilizing 9 solvents) and slow solvent evaporation (utilizing 6 solvents) whenever the MNZ solubility allowed for the formation of a solution. Notably, in the evaporative experiments, MNZ readily formed colorless crystals. In the investigated experimental conditions, MNZ was determined to be monomorphic, as confirmed by the analysis of PXRD patterns and IR spectra (ESI Table S1 and Figures S1 and S2). The phase obtained corresponded to the known form.

### 3.2 Experimental MNZ Cocrystal Screening

Cocrystal screening experiments for MNZ were carried out in two stages (Scheme 1). In the first stage, stirred suspension crystallization was performed using six coformers (NCT, ADP, LMA, DLMA, GNT, and RES) in eight solvents with different physicochemical properties (MeOH, EtOH, *i*-PrOH, *n*-BuOH, *t*-BuOH, ACN, TOL, and Clf). The coformers were selected based on their molecular features, that is, exhibiting hydrogen bond donor and acceptor groups and aromatic/nonaromatic groups. Structural (PXRD and IR) and thermal [hot-stage microscopy (HSM) and DSC] analyses were used to identify the solid phase(s) produced. Only one cocrystal, the 1:1 MNZ-GNT cocrystal (form II°), was obtained (phase pure from ACN, TOL, Clf, *t*-BuOH, and *n*-BuOH) (ESI Figure S9). Experiments with the other coformers yielded only physical mixtures, as confirmed by PXRD and IR (ESI Figures S3–S8).

Based on the step one results, additional aromatic carboxylic acid derivatives (3-HBA, 4-HBA, 4-ABA, OPHTA, BA, SAL, and GAL) were chosen as potential coformers in the second stage of the MNZ cocrystal screening. In addition to the MNZ-GNT cocrystal, two polymorphs of the MNZ-GAL 1:1 cocrystal were obtained–form I° from ACN and form II from TOL and Clf. Experiments with other stage 2 coformers did not yield cocrystals as the PXRD patterns recorded were attributed only to the individual starting materials (ESI Figures S10–S15).

The three MNZ cocrystals (MNZ-GAL I°, MNZ-GAL II, and MNZ-GNT II°), including the effect of solvent and crystallization technique, were then thoroughly investigated.

### 3.3 MNZ-GAL Cocrystals

**Figure 1:**
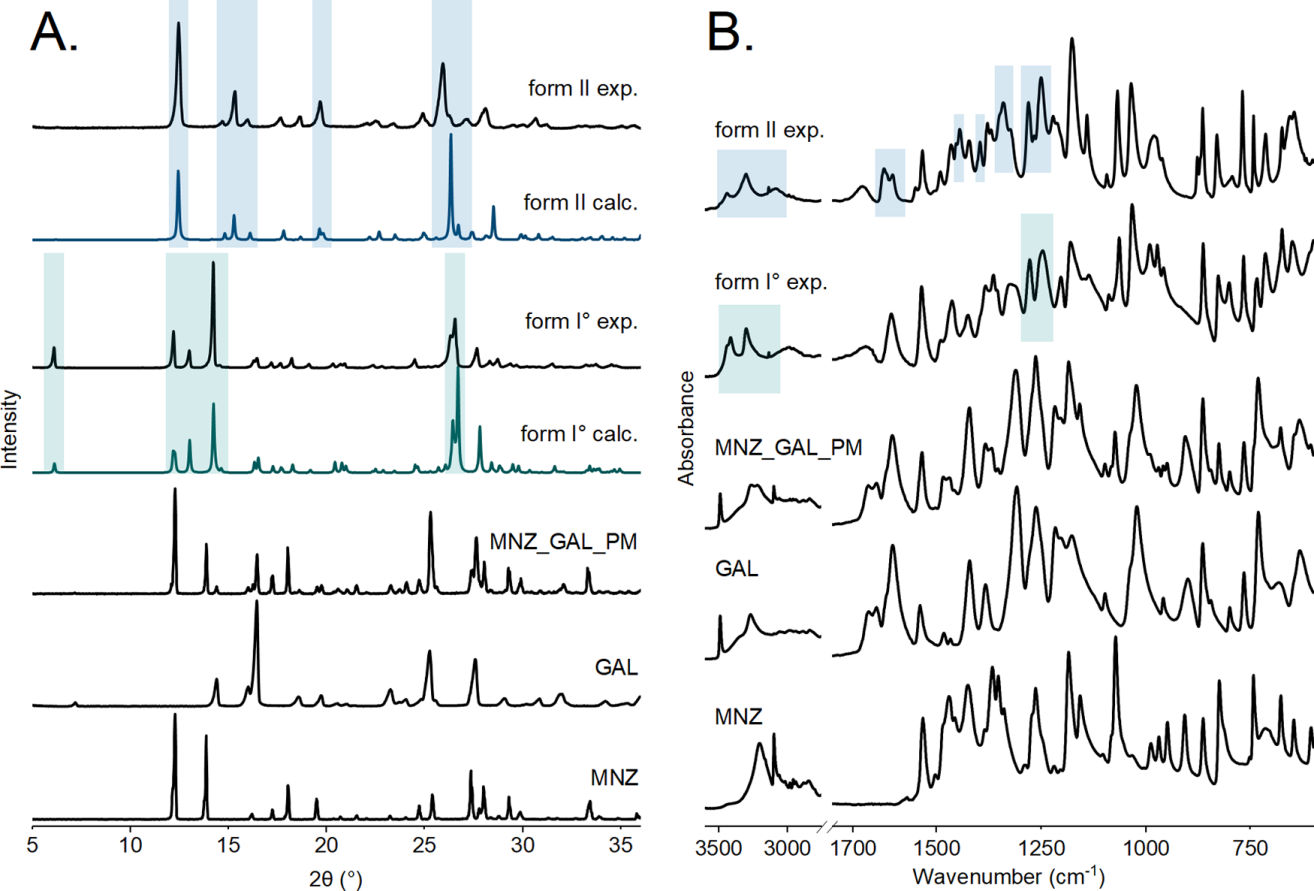
PXRD patterns (A) and IR spectra (B) of the starting materials (MNZ and GAL), their 1:1 physical mixture, and experimentally obtained MNZ-GAL cocrystals forms I*°* and II (form I° exp. and form II exp.). From single-crystal structures, simulated PXRD patterns of both cocrystal forms are also shown (form I° calc. and form II calc.). Characteristic reflection positions and absorbance bands are marked: green – MNZ-GAL cocrystal form I*°;* blue – MNZ-GAL cocrystal form II.

The MNZ-GAL cocrystal formation was confirmed by comparing the experimental diffractograms to the ones obtained from single-crystal structures calculated PXRD patterns (polymorph I° - VOKYEC^27^ and polymorph II - VOKYEC01^28^). GAL itself is known to be a complex polymorphic system with three anhydrous forms (I–III) (CSD Refcode-family: IJUMEG), five monohydrates, and many solvates. Anhydrous forms I and III are the metastable polymorphs; form II° is the thermodynamic form at RT and was used in this study as a starting material.^50,72−74^ The diffractograms of both cocrystal forms lacked the reflection positions assigned to MNZ and GAL present in the diffractogram of the 1:1 physical mixture, which confirmed the full conversion into the cocrystals (Figures 1A, S16A, and S17A). Characteristic form I° peak positions occur at 2θ = 6.12, 12.15, 13.02, 14.20, and 26.58°. Characteristic form II reflection positions occur at 2θ = 12.47, 15.20, 19.52, and 25.75°.

ATR-FTIR spectroscopy was also used to confirm cocrystal formation. Spectra of the obtained samples were compared to the spectra of the starting materials, as well as their 1:1 physical mixture. The physical mixture spectrum showed the characteristic bands of the separate starting materials. In contrast, both cocrystal forms exhibited new distinct band positions in the range of 3000–3500 cm^–1^ which includes ν(O–H) vibrations. MNZ-GAL form I° displayed new bands at 3445, 3289, and 3138 cm^–1^, while form II exhibited new bands at 3444, 3301, 3090, and 2975 cm^–1^. Additionally, the spectra of both cocrystal forms differed from the physical mixture in the range of 1200–1450 cm^–1^, indicating the formation of new O–H···N intermolecular hydrogen bonds. Form I° exhibits bands at 1429, 1370, 1317, and 1206 cm^–1^, whereas the band positions characteristic for form II are found at 1622, 1603, 1445, 1396, 1340, and 1217 cm^–1^ (Figures 1B, S16B, and S17B).

#### 3.3.1 Selective Crystallization of MNZ-GAL Cocrystal Polymorphs Using Stirred Suspension Crystallization

To investigate the impact of solvent properties on the polymorphs of MNZ-GAL, we conducted polymorphic screening using 30 solvents with varying polarities (Table 1). Stirred suspension experiments carried out with solvents having a relative polarity (RP) above 0.35 resulted in the formation of form I° within 1 week. During this time, samples were periodically withdrawn and analyzed using PXRD/IR measurements, revealing the initial formation of cocrystal form II, which subsequently transformed into form I°. In contrast, solvents with an RP below 0.35 produced either form II or mixtures of the starting materials and form II within 1 week. No formation of form I° was observed with this second group of solvents. To investigate this further, the stirring time was extended to 2 weeks at 500 rpm, and a temperature cycle program (between 10 and 30 °C) was implemented. These modifications led to complete conversion into cocrystal form II within 5–7 days (in some cases 14 days; see Table 1), but no transformation into form I° was observed. Notably, three solvents (DMPU, NMP, and D-LIM) did not facilitate the formation of any MNZ-GAL cocrystal polymorphs during the initial or extended stirring period.

**Table 1:** MNZ-GAL Cocrystal Phases Obtained from Stirred Suspension (Slurry) Crystallization, ES, and SD

solvent	relative solvent polarity	polarity index	dielectric constant	slurry (time until full conversion occurred)	electrospraying	spray drying
HEX	0.009	0.1	1.9	II (2 weeks)	b	b^,^^c^
PhenPrOH	nonpolar^a^	a	a	II (1 week)	b	b^,^^c^
isSORB	nonpolar^a^	a	a	II (2 weeks)	d	c
D-LIM	a	a	2.4	PM (2 weeks)	d	c
TOL	0.099	2.4	2.4	II (1 week)	b	b^,^^c^
Is-PrEt	a	2.2	3.9	II (5 days)	b	b^,^^c^
ETDE	0.117	2.8	4.2	II (5 days)	b	b^,^^c^
BuMetEt	0.124	2.5	a	II (1 week)	b	b^,^^c^
DCM	a	3.1	8.9	II (1 week)	PM	b^,^^c^
BuAc	a	4.0	5.0	II (1 week)	II	II
THF	0.207	4.0	7.6	II (1 week)	II	b
EtAc	0.228	4.4	6.0	II (1 week)	II	II
BTF	0.241	a	9.40	II (2 weeks)	b	b^,^^c^
Clf	0.259	4.1	4.8	II (1 week)	b	b^,^^c^
DCE	0.269	a	10.4	II (1 week)	b	b^,^^c^
MIBK	a	4.2	13.1	I° (1 week)	b	b^,^^c^
DMPU	0.352	a	36.1	PM (2 weeks)	d	c
2-PENT	0.321	a	15.5	I° (1 week)	b	b^,^^c^
ACT	0.355	5.1	20.6	I° (1 week)	II/I° + II	II
*t*-BuOH	0.389	a	10.9	I° (1 week)		c
ACN	0.460	5.8	37.5	I° (5 days)	b	b
*i*-PrOH	0.546	3.9	18.6	I° (1 week)		
*i*-BuOH	0.552	4.0	17.9	I° (1 week)		
*n*-BuOH	0.586	a	17.8	I° (1 week)		
*n*-PrOH	0.617	4.0	20.45	I° (5 days)		
AcCOOH	0.648	a	6.17	I° (5 days)	b	b^,^^c^
EtOH	0.654	5.2	24.5	I° (3 days)	II	II
MeOH	0.762	5.1	32.7	I° (3 days)	II	II
NMP	a	6.7	a	PM (2 weeks)	d	c
water	1.000	10.2	80.1	I° (3 days)	II	II

a Data not found.

b Component(s) solubility too low.

c Not suitable to the method.

d Solvent does not evaporate under the experimental conditions tested. Solvents are sorted according to their RP values from the literature or (if no RP data was found) according to their polarity index or dielectric constant values from the literature.^75−77^ I*°* - MNZ-GAL cocrystal form I°; II – MNZ-GAL cocrystal form II; *PM* – physical mixture of the starting materials.

Thus, in slurry experiments, the initial cocrystal formed is form II, which subsequently undergoes transformation into form I°. The transformation kinetics is significantly influenced by the solvent polarity and cocrystal solubility in the organic solvents. A longer experiment duration or an increase in the temperature is expected to promote the transformation from form II to form I°, irrespective of the solvent used. Form I° is the thermodynamically favored form at RT. On the other hand, form II exhibits metastability but simultaneously demonstrates high kinetic stability, as no transformation into form I° was observed within a year of storing the sample under ambient conditions (end of the investigation period).

#### 3.3.2 Selective Form II Cocrystallization Using ES Deposition

**Figure 2:**
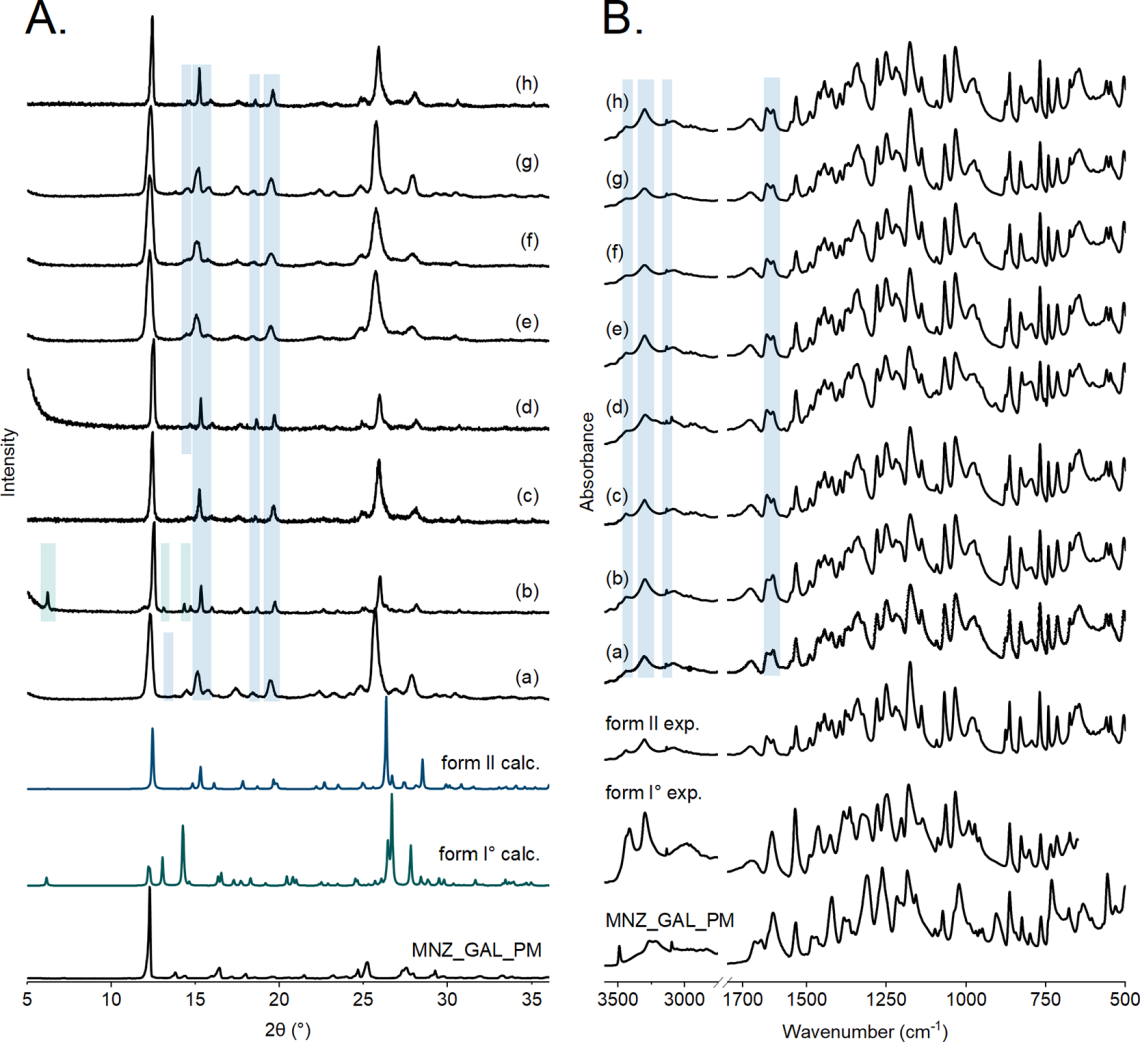
ES experiments: PXRD patterns (A) and IR spectra (B) of MNZ-GAL 1:1 physical mixture, MNZ-GAL cocrystal simulated from the crystal structures of forms I° and II, and the MNZ-GAL cocrystals obtained from (a) ACT deposited on the collector, (b) ACT crystallized on the emitter, (c) H_2_O, (d) EtAc, (e) EtOH, (f) MeOH, (g) THF, and (h) BuAc. Characteristic reflection positions and absorbance bands are marked (green – MNZ-GAL cocrystal form I*°;* blue – MNZ-GAL cocrystal form II).

To facilitate MNZ-GAL cocrystal formation, the ES process parameters were adjusted for eight solvents: EtAc, BuAc, EtOH, MeOH, THF, DCM, ACT, and H_2_O, covering solvents resulting either in form I° and form II in stirred suspension crystallizations (Table 1). The solution feeding rate, the working distance, the applied voltage, and the emitter diameter (needle size) had to be optimized. The conditions chosen for each experiment are listed in ESI Table S4. Rapid solvent evaporation during ES experiments drove the formation of cocrystal form II from all investigated solvents except DCM, as confirmed with PXRD and IR (Figure 2). In the case of the ACT electrospray experiment, two distinct PXRD patterns were recorded, one for the crystals deposited on the metal collector (cocrystal form II) and one for the crystals formed at the tip of the emitter throughout the spraying process (mixture of cocrystal forms I° and II). The solution collected on the metal emitter may have contributed to the formation of both forms as variable evaporation conditions were present.

We also investigated other solvents that had sufficient solubility for both starting compounds to determine the optimal experimental conditions. However, for D-LIM, isSORB, and NMP, suitable ES parameters could not be found due to the low volatility of these solvents. [D-LIM is described in the literature as having an “oil-like appearance” and has a high relative vapor density of 4.7 (air = 1) and a low vapor pressure of 0.19 kPa at 20 °C; these properties make this solvent unlikely to evaporate during an ES experiment. Similar behavior is to be expected from isSORB and NMP with their vapor pressures of 0.013 and 0.039 kPa at 25 °C, respectively, and the relative vapor density of NMP of 3.4 (air = 1).^96−98^]. Despite testing several low flow rates and large needle-to-collector distances, no dry product was deposited on the collector. Hence, solvents such as alcohols (MeOH and EtOH), acetates, or THF, which exhibit much higher vapor pressures and lower relative vapor densities, are much more suitable for a technique that relies on rapid solvent evaporation as the driving factor for crystallization.

#### 3.3.3 Selective Form II Cocrystallization Using SD

**Figure 3:**
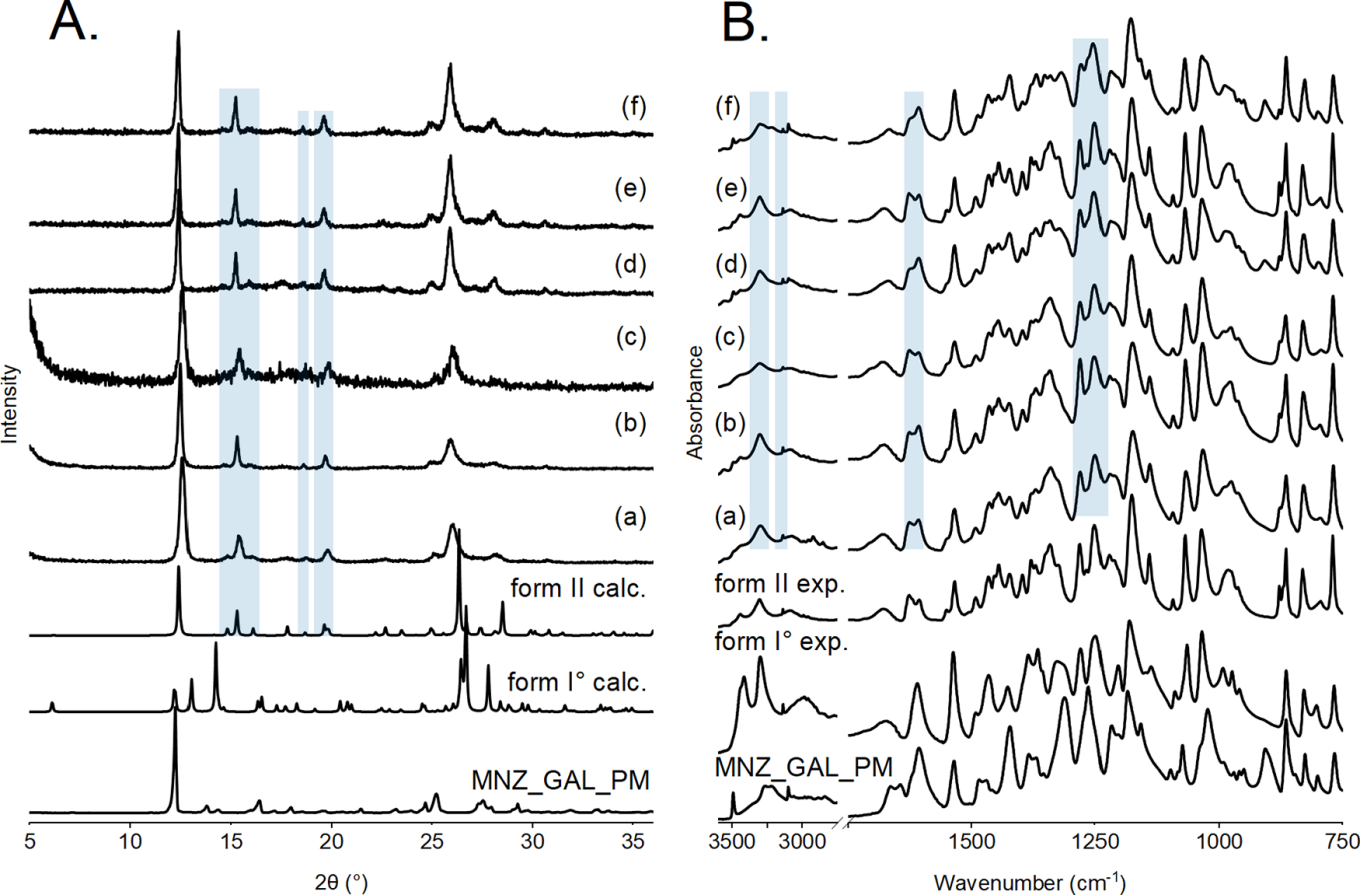
SD results: PXRD patterns (A) and IR spectra (B) of MNZ-GAL 1:1 physical mixture, MNZ-GAL cocrystal simulated from the crystal structures of form I° and II, and the SD products obtained from (a) ACT, (b) H_2_O, (c) EtAc, (d) BuAc, (e) MeOH, and (f) EtOH. Reflection positions and absorbance bands characteristic for MNZ-GAL cocrystal from II are marked in blue.

The suitability of six solvents (ACT, H_2_O, EtAc, BuAc, MeOH, and EtOH) for the formation of MNZ-GAL cocrystals using SD was investigated. To account for the boiling points of the solvents, the inlet drying temperature and the pump speed were adjusted. Stirred suspension experiments using ethyl and butyl acetate produced form II, while using ACT, H_2_O, MeOH, and EtOH led to cocrystal form I° (ESI Figures S16 and S17). In SD experiments, cocrystal form II was exclusively produced (Figure 3), similar to the results obtained from ES (Table 1). The formation of the metastable polymorphs is likely triggered by the rapid solidification during the drying process, which is further heightened by the elevated temperature of the drying medium (nitrogen).

#### 3.3.4 Thermal and Thermodynamic Stability of the MNZ-GAL Cocrystal Polymorphs

**Figure 4:**
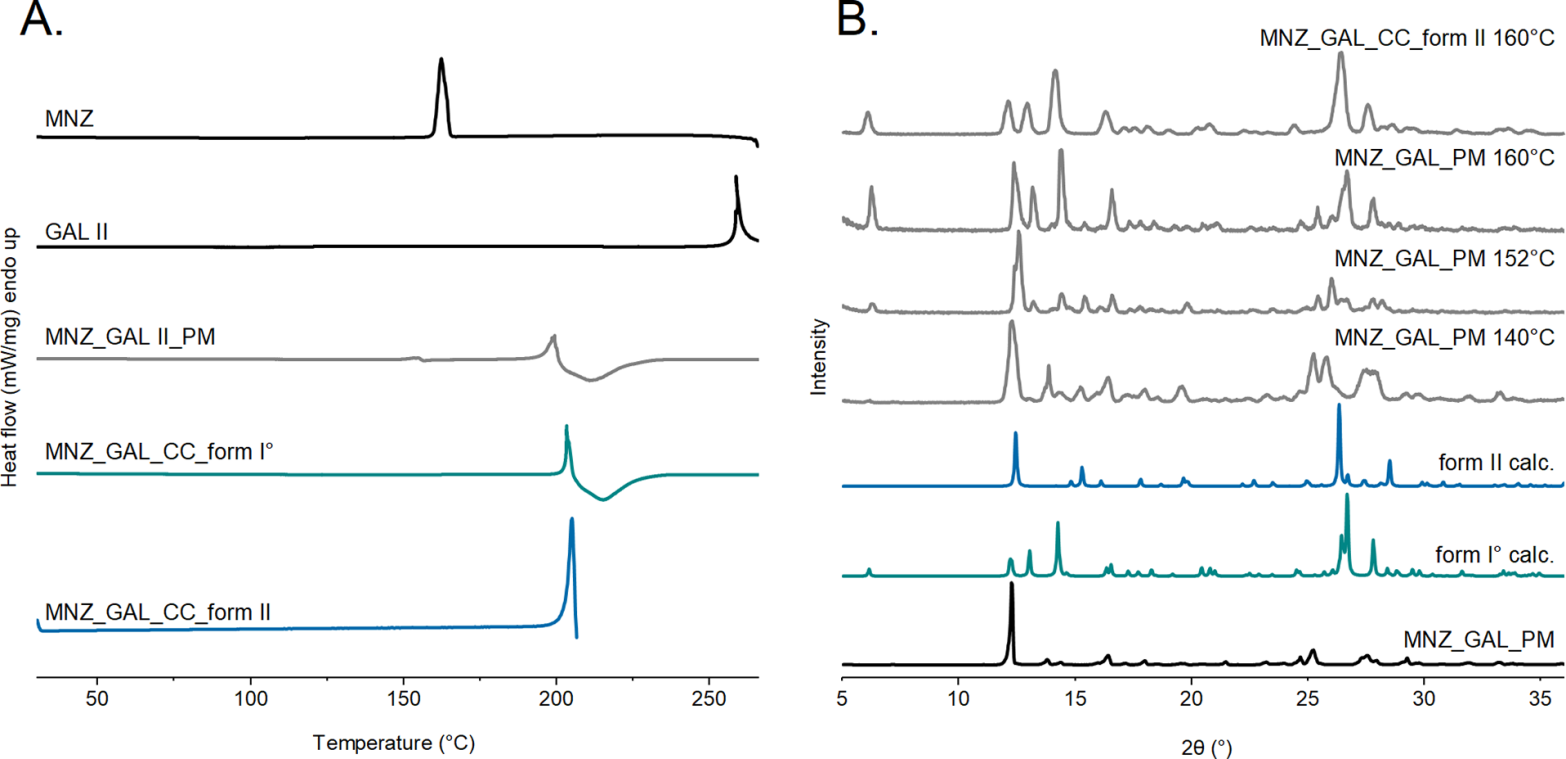
DSC thermograms (A) and PXRD patterns (B) of starting materials (MNZ and GAL II), the MNZ-GAL 1:1 physical mixture, and MNZ-GAL cocrystal forms I*°* and II (calc; CSD VOKYEC^27^ and VOKYEC01^28^, respectively). The MNZ-GAL 1:1 physical mixture was heated up to 140, 152, and 160 °C, and cocrystal form II was heated up to 160 °C.

DSC thermograms were recorded for both starting materials and both cocrystal polymorphs of MNZ-GAL (Figure 4A). The melting points of MNZ and GAL form II° are 159.7 ± 0.3 and 260.0 ± 0.3 °C, respectively, and are in agreement with the literature data.^50,78^ The thermogram of the 1:1 MNZ-GAL physical mixture exhibits a small thermal event with an onset temperature of 152.2 ± 0.1 °C, wherein melting, recrystallization, and a polymorphic phase transformation coincide (see next paragraph). Concomitantly, a crystallization process was visible at approximately 157 °C in HSM investigations. The resulting phase was confirmed with PXRD to be cocrystal form I° (see the next section). The DSC traces of the two cocrystal polymorphs look superimposable. At approximately 200 ± 0.8 °C, melting under decomposition is seen in the DSC curves of cocrystals form I° and II.

To further investigate the crystallization event of the physical mixture at 157 °C and the thermal stability of the cocrystal polymorphs, additional experiments were conducted. The samples were heated to predetermined temperatures and the PXRD patterns were recorded (Figures 4B and S18). When the physical mixture was heated to 140 °C, the PXRD analysis revealed characteristic reflection positions of cocrystal form II. However, it was not a complete conversion as some reflection positions corresponding to the starting materials were still present. As the temperature increased to 152 °C, the sample exhibited peak positions of both cocrystal forms, with form II being more dominant than form I°. The diffractogram of the physical mixture heated to 160 °C showed only the form I° reflection positions, indicating complete conversion. Similarly, when cocrystal II was heated to 160 °C, the PXRD pattern mainly displayed peak positions corresponding to form I°, with only a few reflexes characteristic of form II. Hence, both the 1:1 MNZ-GAL physical mixture and form II of the cocrystal transformed into form I° during the heating process.

The DSC trace of form II did not reveal the transformation event, despite the fact that the form II to I° transformation had occurred (as confirmed with PXRD). Therefore, isothermal solution calorimetric measurements were employed to derive the enthalpy difference between the two polymorphs. The heats of solution (Δ_sol_*H*) of the cocrystal forms I° and II were measured as −1.95 ± 0.11 kJ mol^–1^ and −2.23 ± 0.17 kJ mol^–1^, respectively. The small energy difference of 0.28 ± 0.20 kJ mol^–1^ explains why the transformation was not detectable in the DSC experiments using heating rates of 5 and 10 °C min^–1^. Based on the Δ_sol_*H* values, the results of the slurry experiments at RT and heating experiments show that a monotropic relationship between the two polymorphs could be derived, with form I° being the stable polymorph in the entire temperature range. Long-term storage experiments revealed that the metastable form II exhibits high kinetic stability as no transformation into form I° occurred within 1 year (end of investigation time).

### 3.4 MNZ-GNT Cocrystal

#### 3.4.1 Solvent-Based Crystallization Experiments

**Figure 5:**
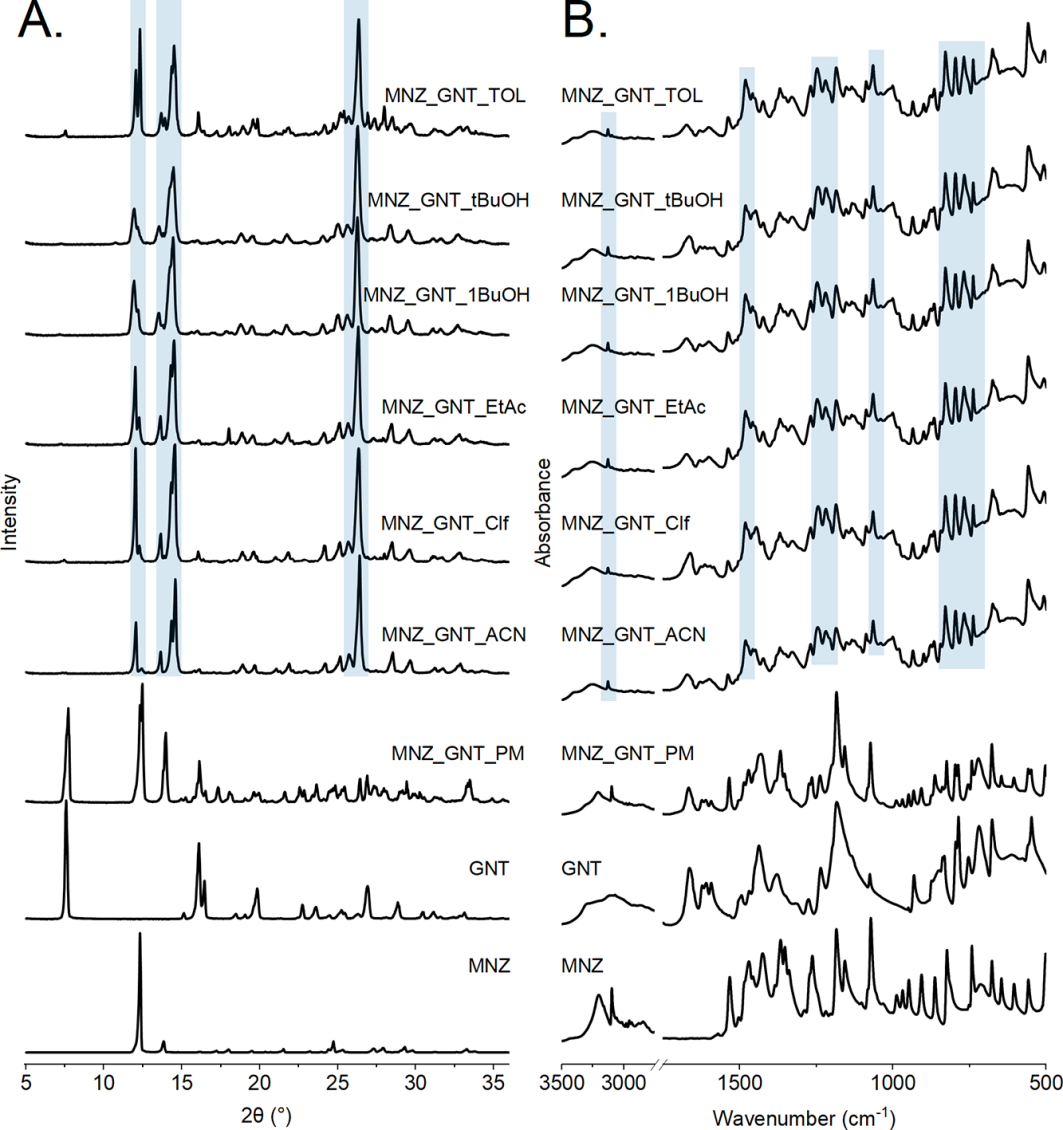
MNZ-GNT cocrystal screen: PXRD patterns (A) and IR spectra (B) of the starting materials (MNZ and GNT II°), their 1:1 physical mixture (MNZ_GNT_PM), and the cocrystals obtained from ACN, Clf, EtAc, *n*-BuOH, *t*-BuOH, and TOL through the stirred suspension method. Reflection positions and absorbance bands characteristic of the MNZ-GNT cocrystal are marked in blue.

In this study, stirred suspension crystallization was employed in various solvents (ACN, Clf, EtAc, *n*-BuOH, *t-*BuOH, and TOL) to produce the MNZ-GNT cocrystal. The obtained cocrystal was then analyzed using PXRD, IR, HSM, and DSC and compared to the starting materials and the 1:1 physical mixture. GNT is known to occur in two polymorphic forms (CSD Refcode-family: BESKAL) with form II° being the ordered monoclinic form, while form I contains a disordered hydrogen bond originating from the 5-substituted hydroxyl group.^49,79−81^ Form II° was used to prepare the physical mixture for the cocrystal screening experiments. The diffractogram of the MNZ-GNT cocrystal lacks the characteristic reflection positions of both reagents (MNZ and GNT form II°) but instead shows new reflexes at 2θ = 11.99, 13.60, 14.25, and 26.92°. Patterns recorded for samples prepared using Clf and TOL showed a GNT form II° residue, which manifested as a low-intensity peak at 2θ = 7.74° (Figure 5A).

MNZ-GNT cocrystal formation was further confirmed by FTIR spectroscopy (Figure 5B). No changes in peak positions were observed for the physical mixture. All IR spectra for the suspension crystallization experiments exhibited similar peak positions to each other but displayed differences in key band positions compared to the individual components. A characteristic of the MNZ-GNT cocrystal is the band at 3132 cm^–1^, which is shifted to higher wavenumbers compared to MNZ. Additionally, new bands appeared in the range <1500 cm^–1^ at 1427, 1333, 1222, 1084, 1007, and 675 cm^–1^.

In addition to the slurry experiments, ES, SD, and freeze-drying experiments were employed to investigate the feasibility of MNZ-GNT cocrystallization. All three methods resulted in the formation of the MNZ-GNT cocrystal (ESI Figure S19), which exhibited the distinctive and intense yellow color characteristic of this multicomponent solid-state form. The PXRD pattern obtained for the freeze-dried cocrystal displayed some peak broadening, indicating a lower degree of crystallinity compared to the other products. This observation aligns with the fact that freeze-drying is typically utilized for the preparation of amorphous samples, although it has also been documented as a method for cocrystallization.^82^

Regarding the ES experiment conducted with water, no cone or mist formation was observed below a voltage of 21–22 kV (at a 20 cm working distance). This is because droplets emitted from the sprayer can disintegrate into a mist only when the electrical forces overcome the surface tension of the liquid being processed. Water has a relatively high surface tension (72.02 mN m^–1^ at RT)^83^ compared to the organic solvents used in this study, necessitating a higher voltage to facilitate mist formation. Additionally, due to the low volatility of water, a longer working distance of 20 cm was utilized, which was considerably greater than the working distances employed for the organic solvents (6–10 cm). Nonetheless, both ES and SD methods were successfully applied to produce the MNZ-GNT cocrystal using water.

#### 3.4.2 Thermal and Thermodynamic Stability of the MNZ-GNT Cocrystal Polymorphs

**Figure 6:**
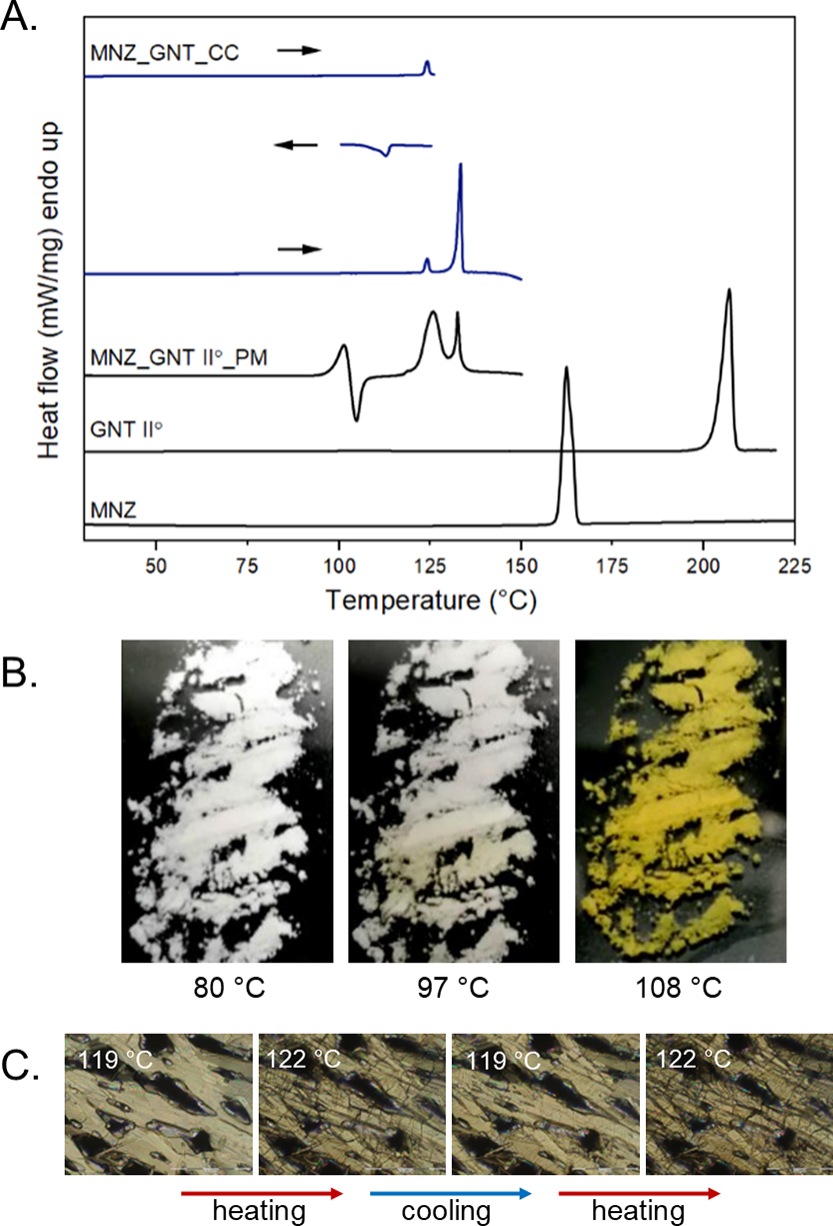
DSC thermograms (A) of MNZ, GNT II°, their 1:1 physical mixture (MNZ_GNT II°_PM), and the cocrystal form II° (MNZ_GNT_CC). CC form II° was subjected to a heating–cooling loop program (as indicated by arrows) to record the reversible phase transformation. Macroscopic images (B) of sample color change upon cocrystal formation from heating of the 1:1 physical mixture of MNZ and GNT II°. HSM images (C) of MNZ-GNT cocrystal form II° showing the reversible II°↔I phase transformation at approximately 122 °C.

DSC experiments were carried out to study the thermal behavior of the API, coformer, and cocrystal as the initial patent disclosed no thermal data on this system. MNZ and GNT form II° thermograms exhibited melting points at 159.7 ± 0.3 and 203.0 ± 0.4 °C, respectively (Figure 6A), which is in accordance with literature data.^78^ The DSC analysis of the 1:1 physical mixture revealed a total of four peaks, all occurring below the melting points of the starting materials. The first endothermic event at 97.5 °C corresponded to the eutectic melting of the two components, followed by a recrystallization process (formation of the cocrystal; conversion incomplete). The third thermal event, an endothermic process, resulted from a eutectic involving the cocrystal (overlapping with the phase transformation described in the next paragraph). Finally, at 131.8 °C, the cocrystal itself underwent melting. To confirm the thermal events associated with the MNZ-GNT physical mixture, the mixture was subjected to heating to observe macroscopic changes (noting that the MNZ-GNT cocrystal has a distinct yellow color) starting from 80 °C. At 97 °C, the mixture started changing from white to pale yellow, which became more pronounced with further heating (Figure 6B). At 108 °C, above the first melting and recrystallization processes observed in the DSC analysis, the entire sample turned yellow. The formation of the cocrystal during these experiments was additionally confirmed with PXRD.

The DSC curve of the cocrystal exhibited an endothermic peak at approximately 123 °C with an enthalpy of 5.32 ± 0.04 kJ mol^–1^. When the heating experiment was halted at 128 °C and the sample was cooled, the reversibility of the process was demonstrated at 113.9 °C (enthalpy of −5.33 ± 0.02 kJ mol^–1^). HSM investigations confirmed the presence of an enantiotropic phase transformation (Figure 6C). The cocrystal described in the literature will be referred to as form II°, while the high-temperature cocrystal will be referred to as form I. Therefore, the endothermic peak with an onset at 131.9 ± 0.1 °C and a heat of fusion of 43.12 ± 0.20 kJ mol^–1^ corresponds to the melting of cocrystal form I.

**Figure 7:**
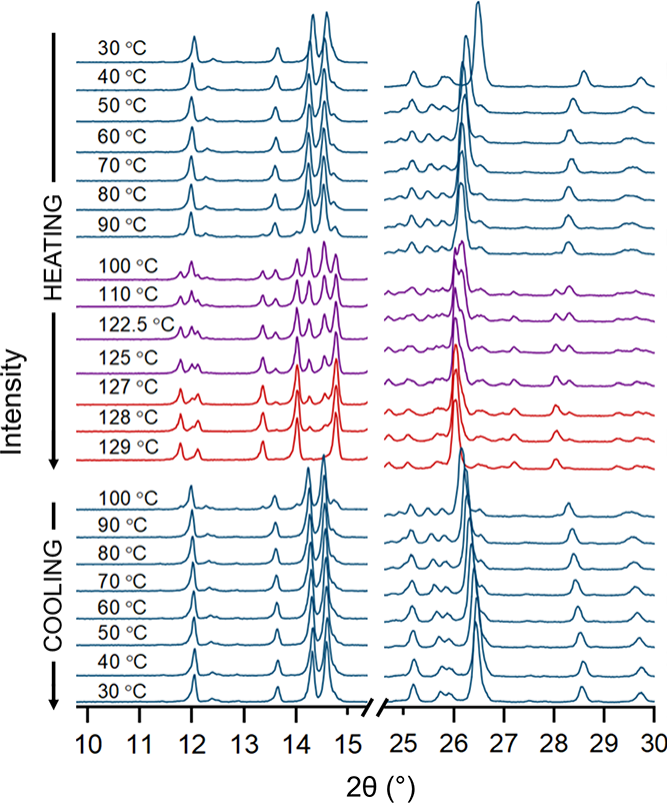
Temperature-dependent PXRD diffractograms of the MNZ-GNT cocrystals. Cocrystal form II° is presented in blue, and the high-temperature form I is presented in red. Purple represents the phase transformation (both polymorphs present) during the heating experiment.

Temperature-controlled PXRD measurements were conducted using the MNZ-GNT cocrystal form II° (Figures 7 and S21). Up to 123 °C, no significant changes were observed apart from shifts in peak positions due to temperature variations. However, at 125 °C, new reflexes appeared at 2θ = 11.8, 12.1, 13.4, 14.0, and 14.8, gradually increasing in intensity as the heating process continued. Concurrently, the reflection positions assigned to the MNZ-GNT cocrystal form II° exhibited a diminishing intensity. Subsequently, the sample was cooled down to 30 °C and the initial recorded MNZ-GNT diffractogram was restored, completing the reverse transformation below 100 °C. The temperatures in the PXRD measurements deviated slightly from the DSC measurements due to the distinct experimental setups (PXRD measurement temperatures are less precise compared to DSC temperatures). The PXRD measurements provide confirmation that the MNZ-GNT cocrystal system undergoes a highly reversible enantiotropic phase transformation at approximately 118.5 ± 4.5 °C, which explains why this phase was not observed in the solvent-based screening experiments conducted at RT.

### 3.5 Crystal Structures and Pairwise Intermolecular Energy Calculations

The crystal structures of MNZ and the GAL and GNT polymorphs have already been published^20−22,50,72−74,79−81^ and are discussed together with the cocrystal structures from the literature, and the structures are determined in this study.

#### 3.5.1 Single-Component Crystal Structures and Their Intermolecular Interaction Features

**Figure 8:**
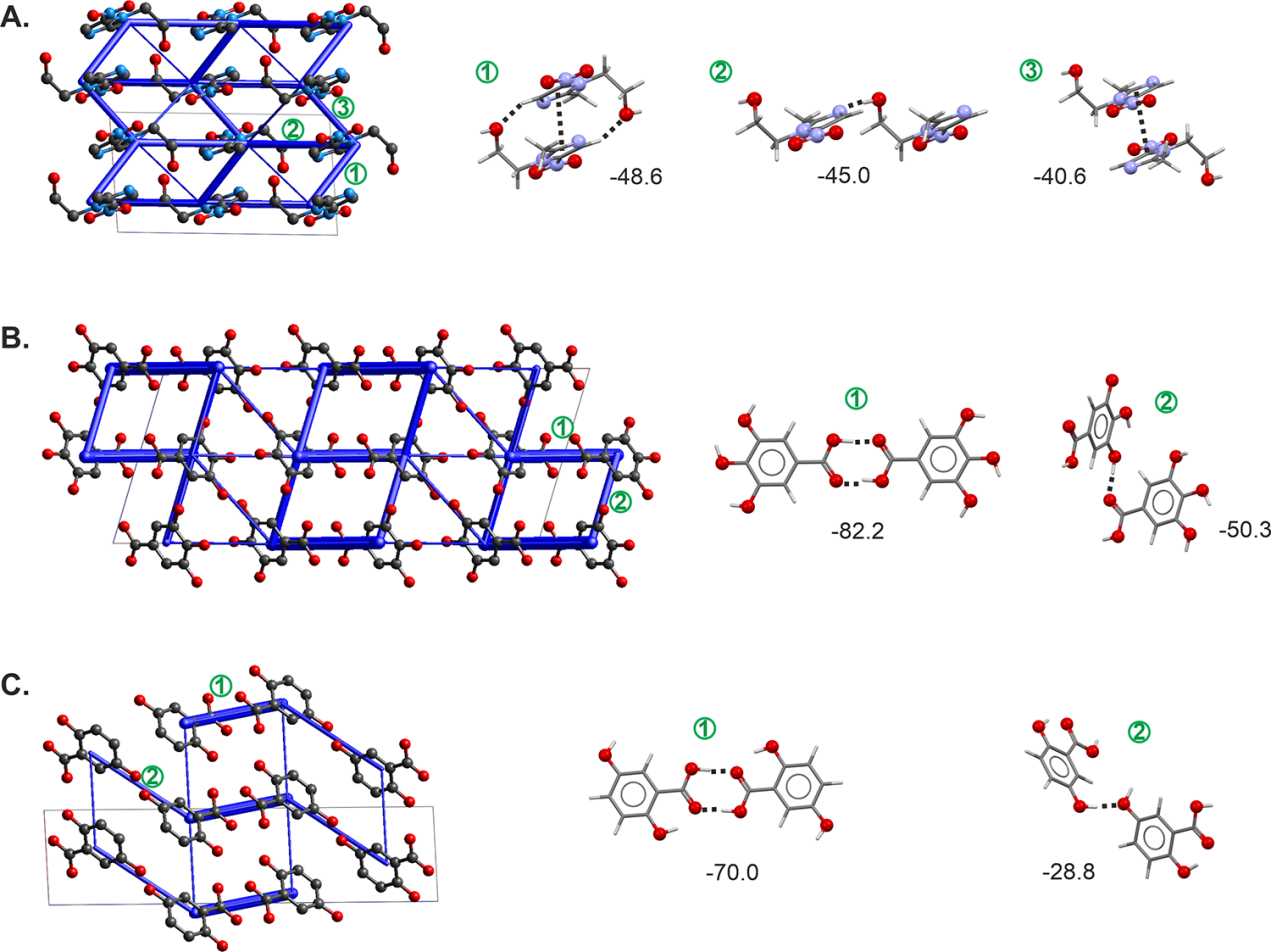
Energy framework diagram (total energy) for MNZ (A), GAL form I*I*° (B), and GNT form I*I*° (C). The energy scale factor is 50. Stabilizing contacts are shown in blue, and the thickness corresponds to the strength. Pairwise interaction energies <5 kJ mol^–1^ are omitted. Strongest pairwise interactions, incl. their energies in kJ mol^–1^, are shown on the right.

MNZ crystallizes in the monoclinic space group *P*2_1_/*c* with Z′ = 1. The molecule features several hydrogen-bonding acceptor groups but only one hydrogen-bonding donor group, the –OH moiety. Despite being a small molecule, MNZ exhibits flexibility, particularly in the –CH_2_–CH_2_–OH group. MNZ and its neighboring molecules form a strong O–H···N hydrogen bond chain motif, which propagates parallel to the *c* axis of the crystal structure. Pairwise intermolecular energy calculations indicate that this hydrogen bond has a strength of −45.0 kJ mol^–1^ (Figure 8A, ESI Table S7). Adjacent stacks of the *C(7)* motif interact via strong π···π interactions. Interestingly, one of the two strong π···π interactions was identified as the strongest pairwise interaction (Figure 8A). This interaction is additionally stabilized by C–H···O close contacts and accounts for −48.6 kJ mol^–1^. The third strongest interaction is the second π···π interaction with a pairwise energy of −40.6 kJ mol^–1^. Hence, MNZ packs tightly and forms strong intermolecular interactions in all three directions within the crystal structure.

Both GAL and GNT are polymorphic containing numerous hydrogen bond donor and acceptor groups along with an aromatic ring moiety. The three GAL polymorphs (CSD Refcode-family: IJUMEG)^50,72−74^ crystallize in *P*1̅, *C*2/*c*, and *P*2_1_/*c*, respectively, with Z’ values of 2 (form I) or 1 (forms II° and III). The GAL molecules of all three polymorphs can be related to the two lowest energy conformations of the molecule, but the positions of the −OH groups differ (from planar orientation) due to strong hydrogen-bonding interactions as described by one of us.^50^ The single component GAL structures all form centrosymmetric *R*_2_^2^(8) acid dimers, which contribute −77.7 to −82.2 kJ mol^–1^ in pairwise energy (ESI Tables S8–S10). The second strongest intermolecular interaction in all polymorphs is an O–H···O_acid_ hydrogen bond. Aromatic interactions (π···π and C–H···π) also significantly contribute to the stability of the crystal packings. Figure 8B depicts the energy framework diagram for GAL II° and its strongest pairwise intermolecular interactions. For GAL I and III, please refer to ESI Figure S26.

GNT (CSD Refcode-family: BESKAL) is a dimorphic compound,^79−81^ differing from GAL in the number and positions of its hydroxyl groups. Both GNT polymorphs crystallize in the monoclinic *P*2_1_/*c* (*P*2_1_/*n*) space group with *Z*′ = 1. In form I, the *m*–OH group is disordered over two positions, and thus the pairwise interaction energy calculations were performed on the Pc (*Z*′ = 2) cell, which contains the two distinct conformations. The form II° conformer and one of the form I disorder positions can be related to the global energy minimum, while the second *m*–OH orientation corresponds to a local minimum.^49^ As with the GAL polymorphs, the *m*–OH protons deviate from planarity due to the formation of strong intermolecular interactions. The *o*–OH groups form strong intramolecular interactions with one of the acid oxygen atoms. In both GNT polymorphs, the strongest intermolecular interaction arises from acid dimers, which were estimated as −72.2 kJ mol^–1^ (I) and −70.0 kJ mol^–1^ (II°) in pairwise energy (Figure 8C). Each of the polymorphs’ *m*–OH groups acts as an acceptor and donor for a strong hydrogen-bonding interaction, O–H···O (−21.0 to −32.1 kJ mol^–1^). Additionally, aromatic interactions stabilize the structures (ESI Tables S11 and S12).

#### 3.5.2 MNZ-GAL Cocrystal Polymorphs

**Figure 9:**
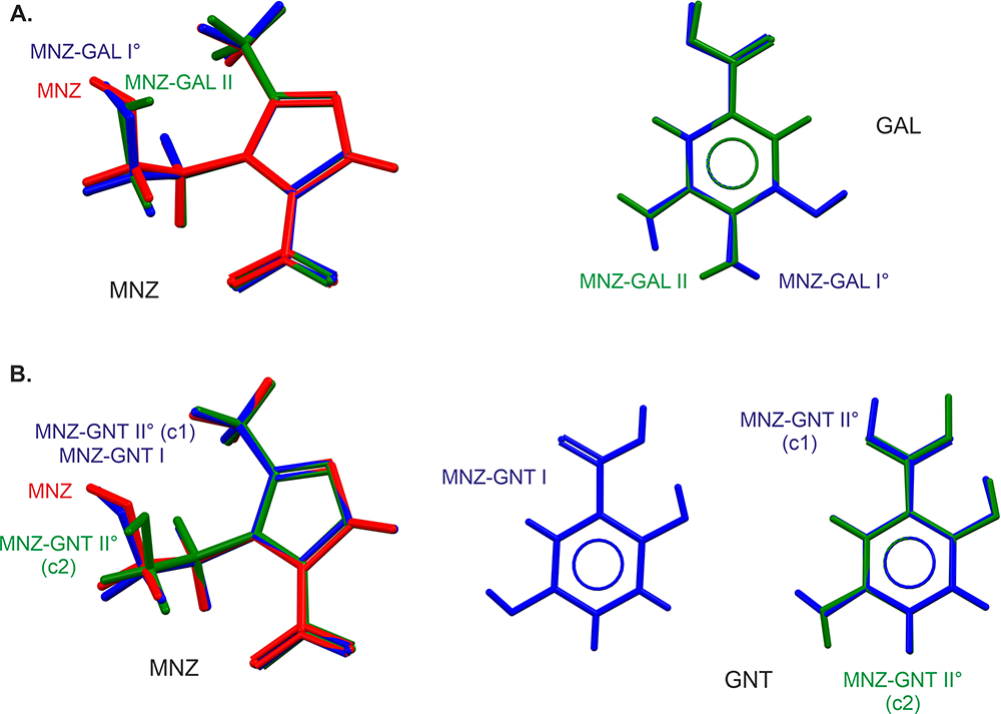
Conformational overlay of MNZ, GAL, and GNT conformers present in the investigated cocrystal structures. Note that for MNZ-GNT form II°, two crystallographically independent MNZ and GNT molecules are present in the structures (denoted *c*1 and *c*2).

The crystal structures of the two MNZ-GAL polymorphs have already been reported.^27,28^ Both MNZ-GAL polymorphs crystallize in the monoclinic space group *P*2_1_/*c* with *Z*′ = 1. The conformation of the MNZ molecule in form I° and neat MNZ is related to the same minimum, whereas in form II, an approximately 180° rotation of the OH group occurs (Figure 9A). The GAL acid conformations of the two cocrystals differ in that in form I°, two intramolecular hydrogen-bonding interactions are formed, whereas in form II only one intramolecular hydrogen bond is present.

**Figure 10:**
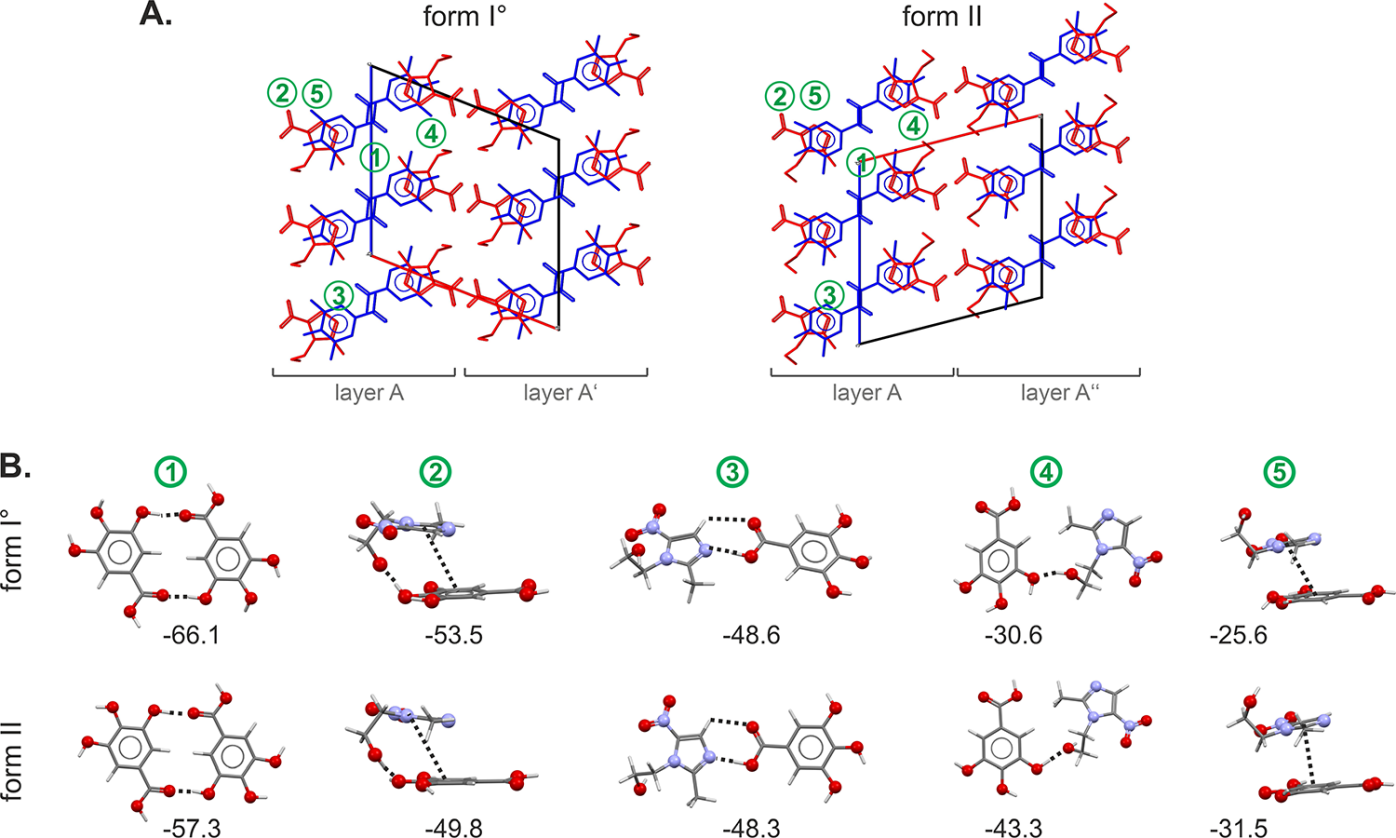
Packing comparison of MNZ-GAL polymorphs (A) viewed along the crystallographic *b* axes. Pairwise intermolecular interactions seen in MNZ-GAL forms I° and II (B). The interactions are depicted in (A), and their energies are given in kJ mol^–1^.

A comparison of the packing of the two polymorphs shows that they share structural similarities. Not only do the two polymorphs crystallize in the same space group but they also have similar lattice parameters and exhibit a 2D packing similarity (as shown in Figure 10A), with identical layers (layer A). The crystal structures differ as adjacent layers are shifted along *b* and *c* resulting in distinct arrangements.

Pairwise intermolecular interaction energy calculations were carried out to determine the strengths of the MNZ···MNZ, GAL···GAL, and MNZ···GAL contacts. Notably, the strongest intermolecular interactions are formed within the identical layers of form I° and II (Figure 10A), indicating that the common building block is a favorable arrangement of MNZ and GAL molecules. The strongest intermolecular interactions are formed between GAL molecules, homomeric strong cyclic O–H···O dimers between one of the hydroxyl groups, and the carbonyl O atom (Figure 10B). This interaction was found to be −57.3 kJ mol^–1^ (II) to −66.1 kJ mol^–1^ (I°) in pairwise energy, which is weaker than the strong GAL *R*_2_^2^(8) dimer seen in the polymorphs of GAL. The second strongest interaction is formed between MNZ and GAL, which is a combination of a strong O–H···O hydrogen bond and a π···π (aromatic) interaction. Interestingly, the directionality of the O–H···O interaction differs between the two polymorphs, with MNZ acting as the acceptor in form I°; in form II, GAL acts as the acceptor and MNZ as the donor. The third strongest interaction observed in the MNZ-GAL cocrystals involves a N atom of MNZ and the –OH group of the GAL acid function as well as a C–H···O close contact (ESI Tables S13 and S14). Additionally, another strong O–H···O interaction is formed between MNZ and GAL (4), with MNZ acting either as the acceptor or donor of the hydrogen bond. For further details on the interaction energies and energy framework diagrams, please refer to ESI Tables S13 and 14 and ESI Figure S27. Based on the pairwise energy calculations, it can be concluded that cocrystallization is not solely driven by any single heteromolecular interaction but instead by the cumulative effect of all interactions observed in the common building blocks (layers) of the two cocrystal polymorphs.

**Figure 11:**
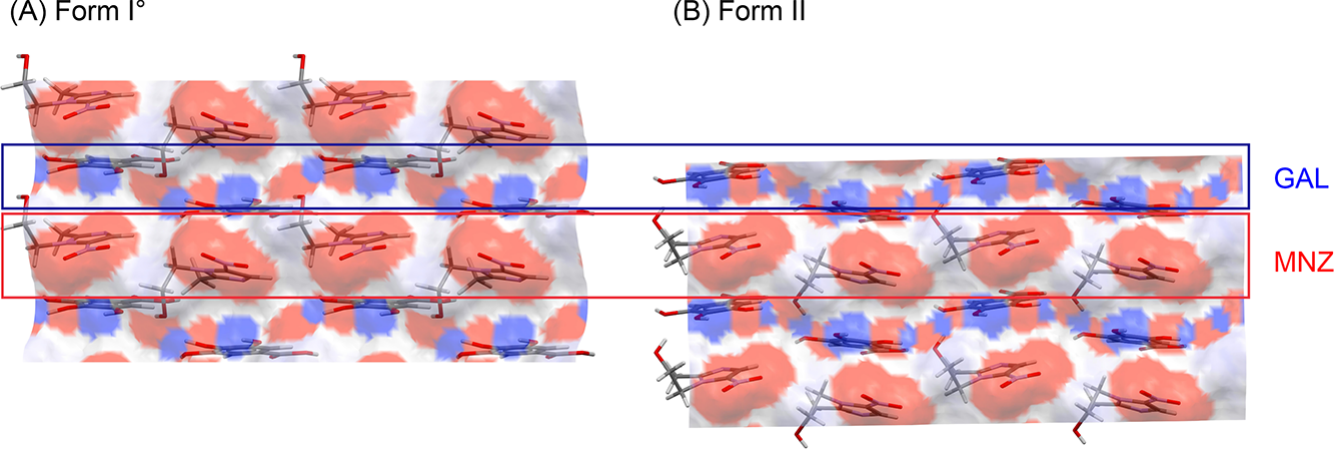
Surface analysis of the MNZ-GAL (1 0 0) planes with offset −7.21 (A) and −7.12 (B). The surfaces are color coded according to H-bond donor (blue) and H-bond acceptor sites (red) by applying a 50% transparency.

As illustrated in Figure 9A, the two polymorphs exhibit distinct orientations of the MNZ and GAL –OH groups, resulting in different directionalities of the hydrogen-bonding interactions and in different 3D packing arrangements. By mapping the hydrogen-bonding donor and acceptor sites onto the surfaces that separate the common layers of the two polymorphs (Figure 11) using the Mercury CSD-Particle Surface Analysis tool,^65^ the disparity between the two forms can be visualized, specifically in terms of the locations of the H-bond acceptor and H-bond donor sites. A careful selection of solvent properties, that is, solvent polarity, may therefore allow a specific surface solvation of crystals, thereby enabling access to different nucleation and crystallization regimes, resulting in optimized conditions for the formation of cocrystal polymorphs.^84,85^

#### 3.5.3 MNZ-GNT Cocrystal Polymorphs

Single crystals of MNZ-GNT form II° were obtained through slow evaporation from water. The crystal structure of form I was determined using PXRD after heating form II° above its transition temperature from form II° to form I (Figure 7).

**Figure 12:**
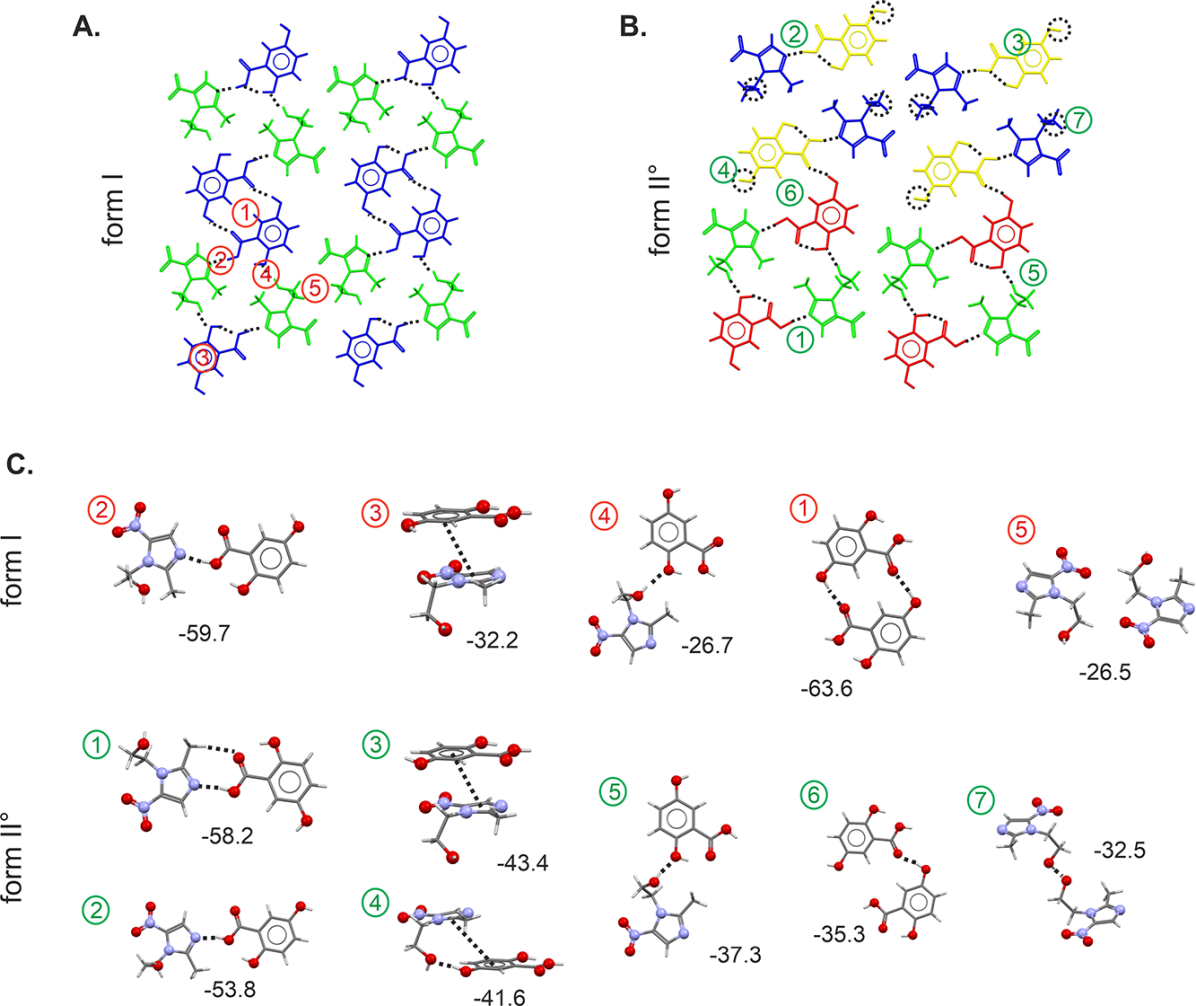
Packing of MNZ-GNT form I (A) and form II° (B) layers. Symmetry-independent molecules are color coded. Pairwise intermolecular interactions seen in MNZ-GNT forms I° and II° (C). The interactions are depicted in (A), and their energies are given in kJ mol^–1^.

Form I crystallizes in the triclinic space group *P*1̅, with one MNZ and one GNT in the asymmetric unit, confirming a 1:1 stoichiometric ratio. The conformation of the MNZ molecule is similar to that observed in MNZ and MNZ-GAL form I (Figure 9B). The GNT molecule of the cocrystal adopts a nearly planar conformation, with the *o*–OH group forming an intramolecular hydrogen bond with the –OH group of the carboxylic acid function (Figure 12A). The *m*–OH group forms a strong intermolecular interaction with an adjacent GNT molecule, related through the inversion symmetry. The pairwise intermolecular interaction of the *R*_2_^2^(14) ring motif was estimated to have an energy of −63.6 kJ mol^–1^, making it the strongest pairwise interaction in the cocrystal. This homomeric interaction is weaker than the strongest interaction seen in the coformer structure (GNT) but stronger than the strongest MNZ interaction observed in the single-component structure. The strongest heteromolecular interaction observed in MNZ-GNT form I, –60 kJ mol^–1^, arises from a strong O–H···N intermolecular interaction (Figure 12A). Other strong heteromolecular interactions in cocrystal form I are due to π···π interactions and the O–H···O hydrogen bond involving the MNZ –OH group as a donor and the GNT *o*–OH group as an acceptor (Figure 12C).

It should be noted that other possible orientations and disorder of the hydroxyl groups in MNZ and GNT are theoretically feasible in cocrystal form I, as seen in GNT form I. However, alternative orientations did not yield improved results in the Rietveld refinement. Thus, the strong hydrogen-bonding interactions are formed within the layers of form I (as shown in Figure 12A), and adjacent layers, related by inversion symmetry, interact through strong π···π interactions.

The second polymorph, cocrystal form II°, also crystallizes in the triclinic space group *P*1̅. The asymmetric unit contains two molecules of MNZ and two molecules of GNT. Figure 9B illustrates that crystallographically independent MNZ and GNT molecules have distinct conformations. Specifically, in MNZ, the orientations of the hydroxyl protons differ, while in GNT, the positions of the COOH group and the *m*–OH hydrogen atom undergo a 180° flip.

Pairwise intermolecular interaction calculations have revealed that the five strongest interactions in the MNZ-GNT cocrystal II° are heteromolecular in nature, occurring between symmetrically independent MNZ and GNT molecules (Figure 12B). These include hydrogen bond interactions, O–H···N (with close C–H···O contacts), O–H···O, and π···π contacts, with the strongest interaction estimated to have a pairwise intermolecular energy of −58.2 kJ mol^–1^. Comparatively, the MNZ-GNT interactions in the cocrystal are stronger than the strongest interactions observed in MNZ (−48.6 kJ mol^–1^) but weaker than the acid dimers observed in GNT forms I and II° (approximately −70.6 kJ mol^–1^). The heteromolecular interactions in cocrystals I and II° have similar strengths. Similar to the case of the MNZ-GAL cocrystals, pairwise energy calculations suggest that the driving force for cocrystallization is not solely attributed to a single intermolecular interaction but rather to the overall energy gain of the cocrystal structures, mediated by the sum of the intermolecular interactions.

**Figure 13:**
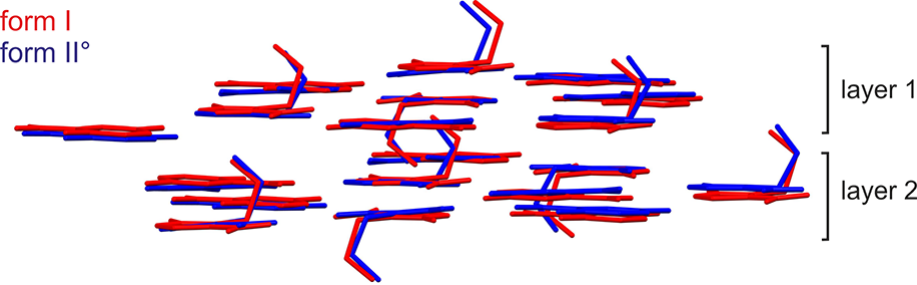
Structure overlay of MNZ-GNT forms I° (red) and II° (blue).

The structure comparison between the MNZ-GNT polymorphs reveals a high degree of packing similarity. Disregarding the hydrogen-bonding directionalities, which differ between the two polymorphs and the crystallographically independent molecules of cocrystal form II° (due to C–COOH and C–OH rotations), the two structures exhibit 3D packing similarity with an rmsd_35_^86^ value of 0.43 Å (Figure 13, only a subset of the cluster is shown). The high structural similarity observed between the two polymorphs may provide a rational explanation for the high reversibility of phase transformation. The need for a 180° rotation of the –COOH and –OH groups, despite the structural similarity, results in a relatively high transition enthalpy of 5.3 kJ mol^–1^.

### 3.6 Virtual Screening Results

In addition to the experimental cocrystal screen, a virtual screening was conducted, which involved the utilization of several tools: MC, MCHBP, and MEP calculations. All coformers that were investigated experimentally were included, along with three additional coformers known for their cocrystal-forming abilities with MNZ: 3,5-dihydroxybenzoic acid,^28^ ethyl gallate,^29^ and pyrogallol.^19^ It is important to note in this context that 3,5-dihydroxybenzoic acid forms a cocrystal hydrate.

It should be noted that the results of the MC screening are somewhat dependent on the conformation of the molecules under consideration.^87^ Therefore, a range of conformations for both MNZ and the coformers was used as the input, including the global gas-phase minima and constrained optimized conformers with dihedral angles based on experimental observations. Foreshortening of X–H was applied. Overall, for all selected coformers, at least one conformation successfully passed the MC screening test, indicating their potential to form cocrystals. This encompassed coformers known to form experimental cocrystals; however, also those not identified to form cocrystals in the present study were included. For more details (calculated molecular descriptors for all the API-CF pairs), please refer to ESI Table S17.

The MCHBP tool analyzes the occurrence of specific intermolecular interactions involving a given functional group in the CSD database. It assumes that the strongest hydrogen bond among all possible donor–acceptor pairs influences the formation of a crystal structure.^88−91^ The MCHBP score was calculated for the MNZ-coformer combinations (Tables 2 and S18). According to the MCHBP calculations, malic acid emerges as the preferred coformer followed by 4-aminobenzoic acid. Other acids and nicotinamide considered in the analysis had scores close to zero, indicating a lower probability of cocrystallization. For ethyl gallate, resorcinol, and pyrogallol, the hydrogen-bonding propensity values between MNZ molecules outweighed the heteromeric values, suggesting a lesser likelihood of cocrystallization. The last three coformers exhibited the lowest coformer-coformer hydrogen-bonding propensity (distinctively lower than the MNZ-coformer propensity). Overall, based on these results, it is not clear-cut to determine which combinations would lead to cocrystallization and which would not, especially since the 3D environment of the crystal packing is not considered in the analysis. Factors in the crystal packing environment might favor combinations with (slightly) negative multicomponent score values over those with positive values.

**Table 2:** Virtual Cocrystal Screening Results Based on MC (PASS/FAIL mark), MCHBP (Highest Calculated Hydrogen Bond Formation Propensities; MCHBP Score), and MEP (Δ*E*_MEP_, Energy Gain Upon Crystallization) Compared with the Result of Experimental Cocrystallization Attempts from This Work and from the Literature

coformer		MC	highest calculated H-bond formation propensity		MCHBP score	mean Δ*E*_MEP_ (kJ mol^–1^)	experimental cocrystal formation
			heterodimeric	homodimeric			
investigated in this work	GAL	PASS	0.65	0.61	0.05	–15.84	yes
	4-ABA	PASS	0.8	0.62	0.18	–13.82	no
	RES	PASS	0.53	0.65	–0.12	–11.95	no
	3-HBA	PASS	0.64	0.6	0.04	–7.35	no
	GNT	PASS	0.64	0.6	0.04	–7.32	yes
	4-HBA	PASS	0.64	0.6	0.05	–6. 97	no
	SAL	PASS	0.63	0.59	0.04	–5.25	no
	MA^a^	PASS	0.84	0.62	0.22	–5.05	no
	OPHTA	PASS	0.6	0.63	–0.03	–2.26	no
	NCT	PASS	0.76	0.77	–0.01	–1.76	no
	ADP	PASS	0.61	0.62	–0.01	–1.47	no
	BA	PASS	0.59	0.62	–0.03	–0.90	no
other literature cocrystals	3,5-DHBA	PASS	0.65	0.6	0.05	–13.59	yes^28^
	PGL	PASS	0.53	0.66	–0.13	–12.30	yes^19^
	GAL-ET	PASS	0.55	0.66	–0.11	–4.14	yes^29^

a MA – malic acid.

MEPs provide insights into the electrostatic properties of molecules, highlighting regions with positive and negative charge distributions. In the context of cocrystal prediction, MEPs are commonly employed to evaluate potential complementary interactions between molecules.^47,92^ Both the gas-phase minima and experimentally observed conformations (obtained through constrained optimization) were considered to account for the conformational influences. According to the work of Musumeci et al., a value of −11 kJ mol^–1^ and lower for the value of Δ*E*_MEP_ indicates a probability of more than 50% for cocrystal formation with either caffeine or carbamazepine. Only the global gas-phase minima were used in the published study. Furthermore, it was noted by the authors that depending on the molecule, different thresholds might be used.^47^ Khalaji et al. showed that the conformation used for the calculations influences the outcome.^87^

In the present work, two approaches have been chosen. First, the mean Δ*E*_MEP_ values were calculated for different conformational combinations of API and coformer in order to account for the flexibility of the molecules (approach 1, Table 2). The Δ*E*_MEP_ values of the five coformers known to form cocrystals with MNZ range from −4 to −16 kJ mol^–1^, with gallic acid showing the highest probability and ethyl gallate showing the lowest. Other coformers that showed high promise for MNZ cocrystallization are 4-aminobenzoic acid and resorcinol. Notably high SD values were found for pyrogallol, ethyl gallate, malic acid, GNT, and GAL in comparison to the other conformers. The latter can be explained by the fact that the experimental molecular conformations of the five coformers differ in the number of strong intra- and intermolecular hydrogen-bonding interaction sites, which affects the strengths of the local maxima and minima and therefore the availability of a functional group to act as a hydrogen bond donor or acceptor, as already described by Khalaji et al. for 2,6-dihydroxybenzoic acid.^87^

In the case of some coformers (PGL, GNT, and 4-HBA), each experimental conformation was found to be significantly different from the others which subsequently influenced the interaction strengths, that is, the GNT conformation found in its cocrystal with telmisartan (CSD GIJSOK)^93^ and pyrogallol observed in its cocrystal with thymine (CSD OGIYUA).^94^ An unexpected 4-HBA conformation was reported in its 1*H*-imidazole solvate (CSD XUBSUJ).^95^ The three conformations were excluded for the second calculation method, as the unusual conformation would significantly influence the outcome as only the lowest Δ*E*_MEP_ for API, coformer, and binary mixture were considered (approach 2, Table 3). In agreement with the first set of calculations, the same five coformers were estimated to show a high probability to cocrystallize with MNZ, and three of those have been confirmed to form MNZ cocrystals.

**Table 3:** Virtual Cocrystal Screening Results Based on the MEP (Δ*E*_MEP_)^a^

coformer		approach 1 (kJ mol^–1^)	approach 2 (kJ mol^–1^)
investigated in this work	GAL	–15.84 ± 2.74	–11.29
	4-ABA	–13.82 ± 0.18	–13.69
	RES	–11.95 ± 1.13	–12.82
	3-HBA	–7.35 ± 1.14	–6.93
	GNT	–7.32 ± 3.10	–9.76
	4-HBA	–6.97 ± 2.96	–9.07
	SAL	–5.25 ± 1.50	–7.23
	MA	–5.05 ± 2.62	–7.76
	OPHTA	–2.26 ± 1.37	–4.18
	NCT	–1.76 ± 0.81	–3.38
	ADP	–1.47 ± 1.07	–0.05
	BA	–0.90 ± 0.37	–1.39
other literature cocrystals	3,5-DHBA	–13.59 ± 1.45	–14.78
	PGL	–12.30 ± 4.08	–16.82
	GAL-ET	–4.14 ± 3.06	–1.48

a Approach 1 is based on the range of experimentally observed conformations, and for approach 2, only the combination of the lowest *E*^MNZ^, *E*^CF^, and binary combination values were used. MA – malic acid.

## 4 Conclusions

MNZ has the ability to form cocrystals with PGL, 3,5- DHBA, GNT, GAL, and GAL-ET. All of these coformers are phenols, which are aromatic compounds, and they contain at least two hydroxyl groups. Additionally, three of the coformers have a carboxylic acid function. The molecular characteristics of the coformers allow for the formation of strong hydrogen-bonding and aromatic interactions. Moreover, the coformers have the ability to compensate for the imbalance between the hydrogen-bonding acceptor and donor groups of MNZ. It appears that having more than one hydroxyl group is crucial for the formation of cocrystals with aromatic carboxylic acids.

The screening of MNZ-GAL and MNZ-GNT cocrystal polymorphs confirmed the dimorphism of the MNZ-GAL system and resulted in a second polymorph of MNZ-GNT. The choice of solvent played a crucial role in determining the polymorphic outcome for MNZ-GAL. Initially, cocrystal form II or a mixture of cocrystal forms I° and II was obtained, which transformed into the thermodynamically stable form I° when solvents with relative polarity values above 0.35 were employed. In these solvents, the cocrystal exhibits better solubility compared to those of solvents with lower polarity values. Conversely, solvents with lower polarity values prompted the formation of form II, and no transformation into form I° was observed. Thus, the polarity of the solvent and cocrystal solubility influence the kinetics of cocrystallization and transformation of the monotropically related system (II → I°). Rapid crystallization techniques, namely ES and SD, exclusively led to the formation of the metastable MNZ-GAL form II. The high kinetic stability of form II can be attributed to its structural features and the small enthalpy difference of 0.3 kJ mol^–1^ between the two polymorphs.

The MNZ-GNT cocrystals exhibit an enantiotropic relationship, with form I being the high-temperature form. The reversible transition occurring at 118.5 ± 4.5 °C involves a change from *Z*′ = 2 ↔ *Z*′ = 1. The two polymorphs are isosymmetric, and the relatively high transition energy of 5.3 kJ mol^–1^ originates solely from the 180° torsional flips of –COOH and –OH groups.

This study also demonstrated the potential of ES, SD, and freeze-drying as methods for cocrystallization. Solvent properties, such as surface tension and volatility, the instrument setup, and process conditions, including the emitter diameter, the solution feeding rate, the applied voltage, the needle-to-collector distance, and the temperature, were identified as crucial factors for obtaining cocrystals.

The range of coformers investigated in this study provided a rigorous evaluation of the virtual cocrystal screening methods. First, the flexibility of the molecules examined influenced the results of MC and MEP calculations. Second, the selected coformers feature resemblance (mainly aromatic molecules, –COOH, and –OH functional groups), but their different tendency toward cocrystallization challenges the MCHBP tool, which focuses on molecular fragments. The MC screening tool falsely indicated the potential for cocrystallization for a large number of coformers. Applying the MCHBP tool, the majority of the coformers produced a result close to 0, indicating that cocrystal and single-component crystallization might be equally favored. Among the three virtual cocrystal screening methods for MNZ, the Δ*E*_MEP_ calculations were able to successfully identify three out of five cocrystal-forming coformers, making it the most effective approach. Overall, the results indicate that when dealing with large and flexible pharmaceuticals, the virtual cocrystal screenings need to be carefully conducted, with particular attention given to the conformations.

Finally, this study emphasizes the need for thorough investigations of cocrystal systems, and further experimental validation data are required to refine the virtual screening techniques.

## Supporting Information Available

MNZ polymorph screening experiment list and results; MNZ cocrystal screening (stage 1 and stage 2) experiment list and results; MNZ-GAL cocrystal polymorphs–slurry experiment results and process parameters for ES and SD experiments; MNZ-GNT cocrystal polymorphs–cocrystallization screen, form II°↔I transformation, and form I and II° structure; pairwise intermolecular energy calculations and energy framework diagrams; and virtual cocrystal screening results (PDF)

## Dedication

^⊥^ Deceased Author
