# Supplementary material for: Metronidazole Cocrystal Polymorphs with Gallic and Gentisic Acid Accessed through Slurry, Atomization Techniques, and Thermal Methods
Source: Cryst Growth Des. 2023 Oct 12;23(11):8241–60. doi: 10.1021/acs.cgd.3c00951 (PMC10626573; doi:10.1021/acs.cgd.3c00951)
Supplement: Supplementary file 1 — cg3c00951_si_001.pdf [file cg3c00951_si_001.pdf]

Electronic Supplementary Information

Metronidazole cocrystal polymorphs with gallic and gentisic acid accessed through slurry, atomization techniques and thermal methods

Aleksandra J. Dyba[1,2], Ewa Wiącek[2], Maciej Nowak[2], Jan Janczak[3], †Karol P. Nartowski[2,4], Doris E. Braun[1,*]

[1]Institute of Pharmacy, University of Innsbruck, Innrain 52c, 6020 Innsbruck, Austria

[2]Department of Drug Form Technology, Wroclaw Medical University, Borowska 211A, 50-556 Wroclaw, Poland

[3]Institute of Low Temperature and Structure Research, Polish Academy of Sciences, P.O. Box 1410, Okolna 2, 50-950 Wroclaw, Poland

[4]School of Pharmacy, University of East Anglia, Norwich Research Park, NR4 7TJ Norwich, United Kingdom

†Deceased Author

*Corresponding Author: Dr Doris E. Braun, Institute of Pharmacy, University of Innsbruck, Innrain 52c, 6020 Innsbruck, Austria, e-mail: doris.braun@uibk.ac.at

Contents

1. MNZ polymorph screening 3
2. MNZ cocrystal screening 5
2.1. Stage 1 cocrystal screening 5
2.2. Stage 2 cocrystal screening 10
3. MNZ-GAL cocrystal polymorphs 14
3.1. Stirred suspension cocrystallization (slurry experiments) 14
3.2. Electrospraying – process parameters for cocrystallization 16
3.3. Spray drying - process parameters for cocrystallization 17
4. MNZ-GNT cocrystal polymorphs 18
4.1. Cocrystallization screen – electrospraying, freeze drying, and spray drying 18
4.2. MNZ-GNT cocrystal form II° ↔ I transformation 19
4.3. MNZ-GNT cocrystal form I structure 20
4.4. MNZ-GNT cocrystal form II° structure 22
5. Pairwise intermolecular energy calculations 24
5.1. Metronidazole 24
5.2. Gallic acid 24
5.3. Gentisic acid 25
5.4. MNZ-GAL cocrystals 26
5.5. MNZ-GNT cocrystals 27
5.6. Energy framework diagrams 29
6. Virtual cocrystal screening 31
6.1. Molecular complementarity 31
6.2. Multi-component hydrogen-bond (MCHB) propensity screen 31
6.3. Molecular electrostatic potential (MEP) maps 32
7.3.1 MNZ–pyrogallol 33
7.3.2 MNZ–ethyl gallate 33
7.3.3 MNZ–4-aminobenzoic acid 34
7.3.4 MNZ–3-hydroxybenzoic acid 34
7.3.5 MNZ–4-hydroxybenzoic acid 35
7.3.6 MNZ–3,5-dihydroxybenzoic acid 35
7.3.7 MNZ–adipic acid 36
7.3.8 MNZ–benzoic acid 36
7.3.9 MNZ–gallic acid 36
7.3.10 MNZ–gentisic acid 37
7.3.11 MNZ–malic acid 37
7.3.12 MNZ–nicotinamide 37
7.3.13 MNZ–resorcinol 38
7.3.14 MNZ–salicylic acid 38
7.3.15 MNZ–*o*-phtalic acid 38
References 39

1. MNZ polymorph screening

Table S1. List of **MNZ polymorph screening** experiments and outcomes.

Technique	Protocol	Solvents used	Outcome
Stirred suspension	200 mg of MNZ suspended in solvent, stirred at 500 rpm at RT for 1 week	toluene (TOL)<br>*t*-BuOH<br>MeOH<br>*i*-PrOH<br>EtOH<br>acetonitrile (ACN)<br>*n*-BuOH<br>H_2O<br>chloroform (Clf)	MNZ form I
Slow evaporation	100 mg of MNZ dissolved in solvent at RT, left to crystallize	EtOH<br>MeOH<br>H_2O<br>acetonitrile (ACN)<br>*i*-PrOH<br>*n*-BuOH	MNZ form I

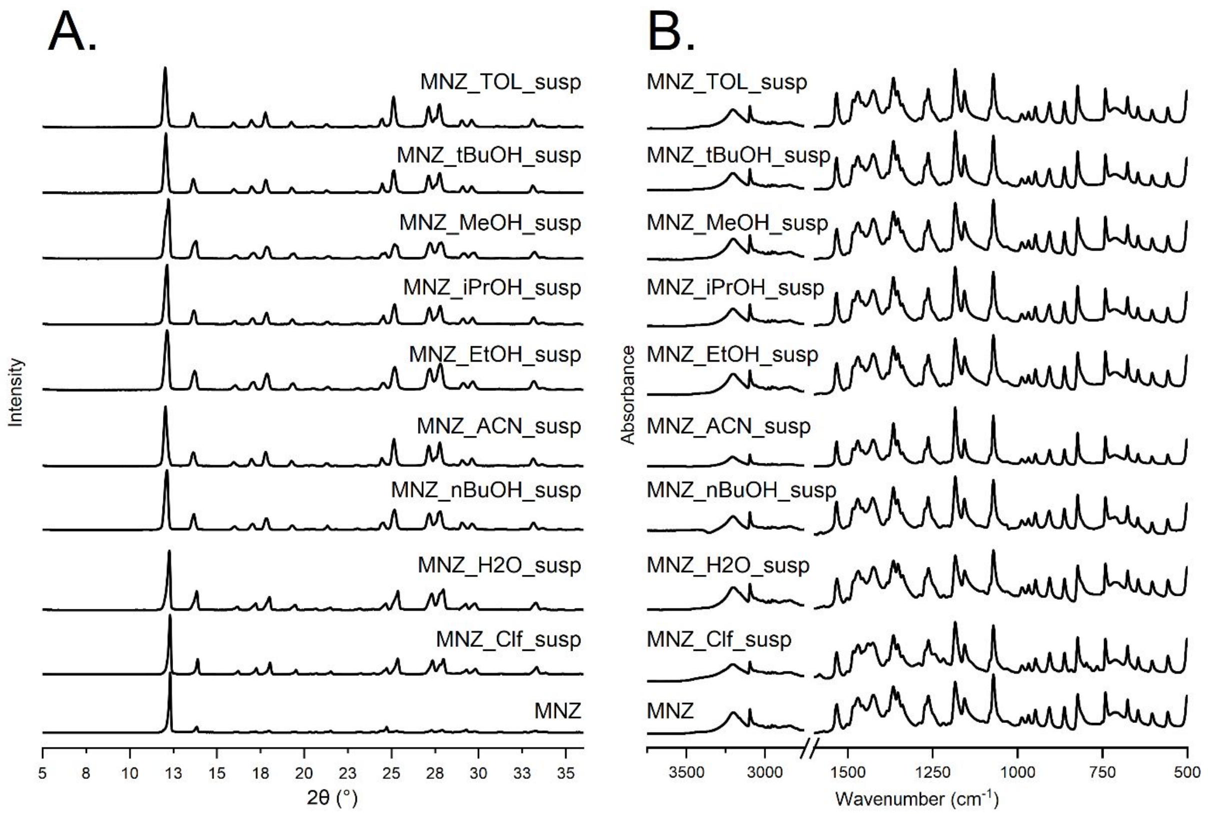

Figure S1. PXRD patterns (A.) and IR spectra (B.) recorded for MNZ **polymorph screening experiments** using the stirred suspension technique.

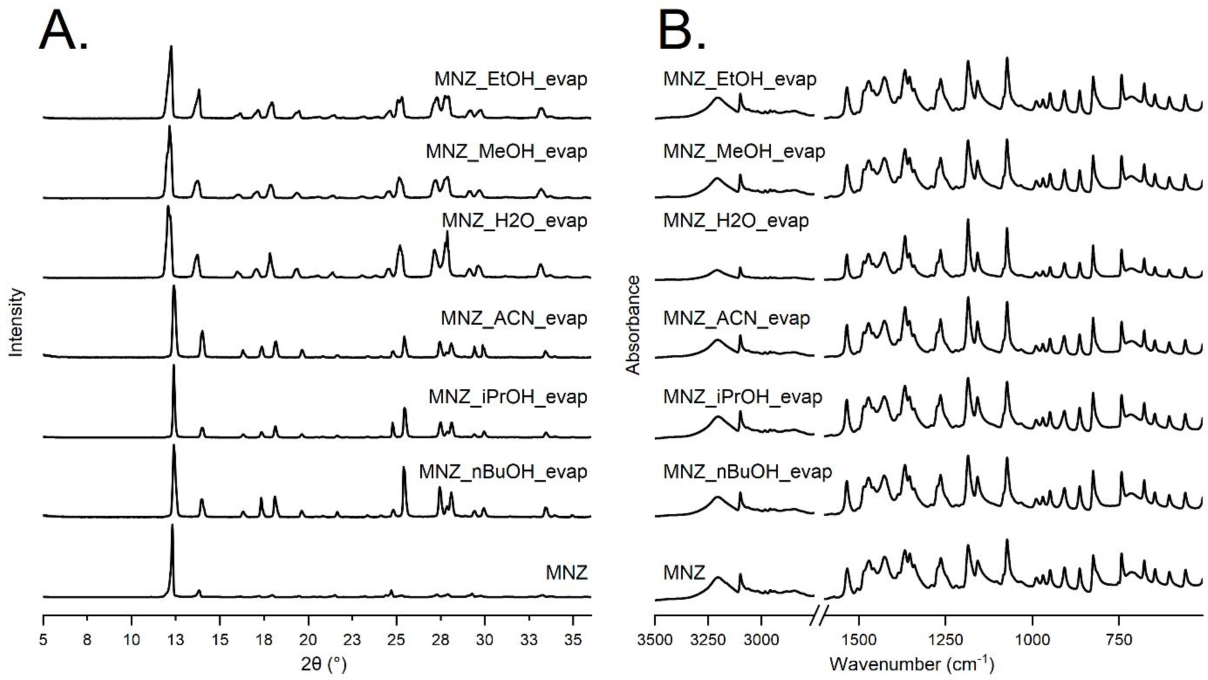

Figure S2. PXRD patterns (A.) and IR spectra (B.) recorded for **MNZ polymorph screening experiments** using slow evaporation.

2. MNZ cocrystal screening

2.1. Stage 1 cocrystal screening – adipic acid (ADP), L-malic acid (LMA), DL-malic acid (DLMA), nicotinamide (NCT), resorcinol (RES), and gentisic acid (GNT)

Table S2. List of experiments and outcomes. *PM* – physical mixture.

Coformer	Technique	Protocol	Solvents used	Outcome
ADP (adipic acid)<br>NCT (nicotinamide)<br>RES (resorcinol)<br>LMA (L-malic acid)<br>DLMA (DL-malic acid)	Stirred suspension	200 mg of 1:1 PM was suspended in solvent and stirred at 500 rpm at RT for 1 week	acetonitrile (ACN)<br>chloroform (Clf)<br>toluene (TOL)<br>*n*-BuOH<br>*i*-PrOH<br>EtOH<br>MeOH<br>*t*-BuOH	PM
**GNT (gentisic acid; 2,5-dihydroxybenzoic acid)**			acetonitrile (ACN)<br>chloroform (Clf)<br>toluene (TOL)<br>*t*-BuOH<br>*n*-BuOH	Cocrystal form II°
			ethyl acetate (EtAc)	Cocrystal form II° and PM
			i-PrOH<br>DMSO<br>EtOH<br>MeOH<br>DMF	PM
NCT (nicotinamide)	Slow evaporation	100 mg of PM dissolved in solvent at RT and left to crystallize	acetonitrile (ACN)<br>EtOH<br>MeOH<br>*i*-PrOH<br>*n*-BuOH	PM

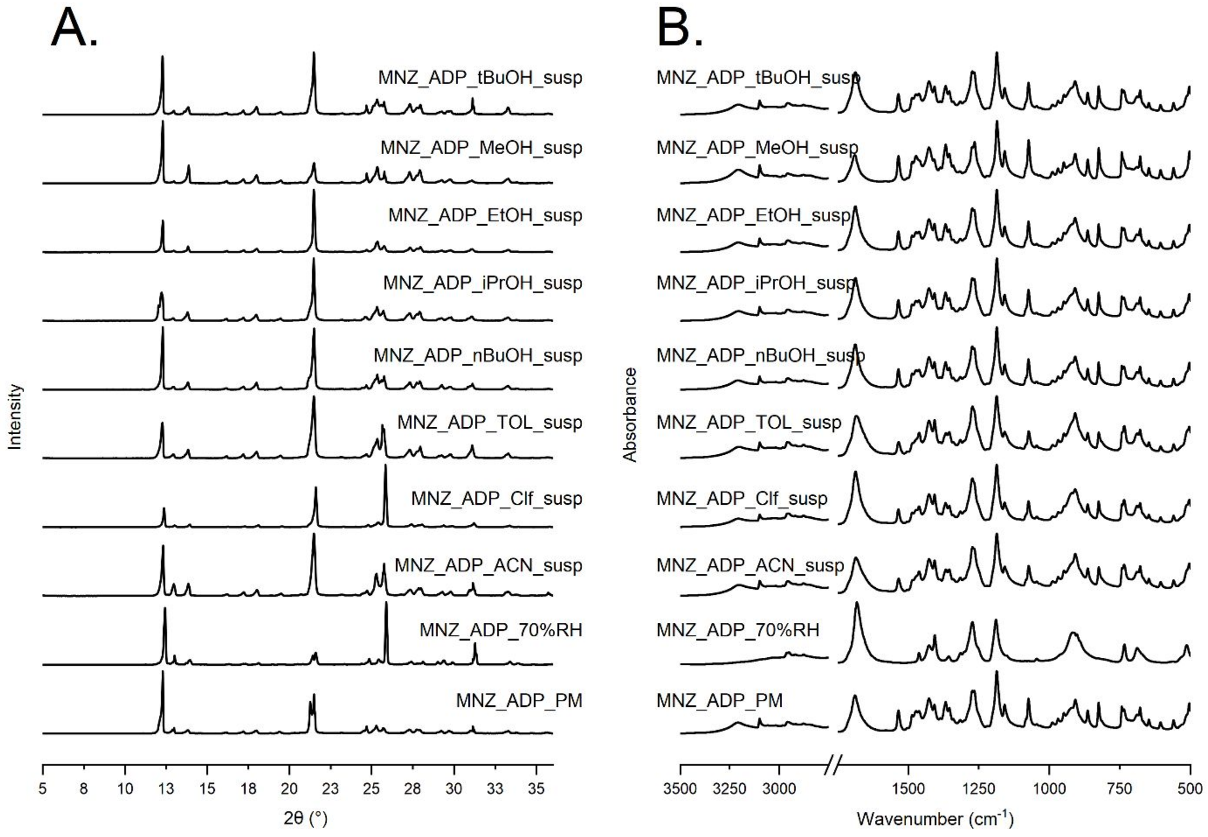

Figure S3. PXRD patterns (A.) and IR spectra (B.) recorded for **MNZ cocrystal screening** experiments using **adipic acid (ADP)** and the stirred suspension technique.

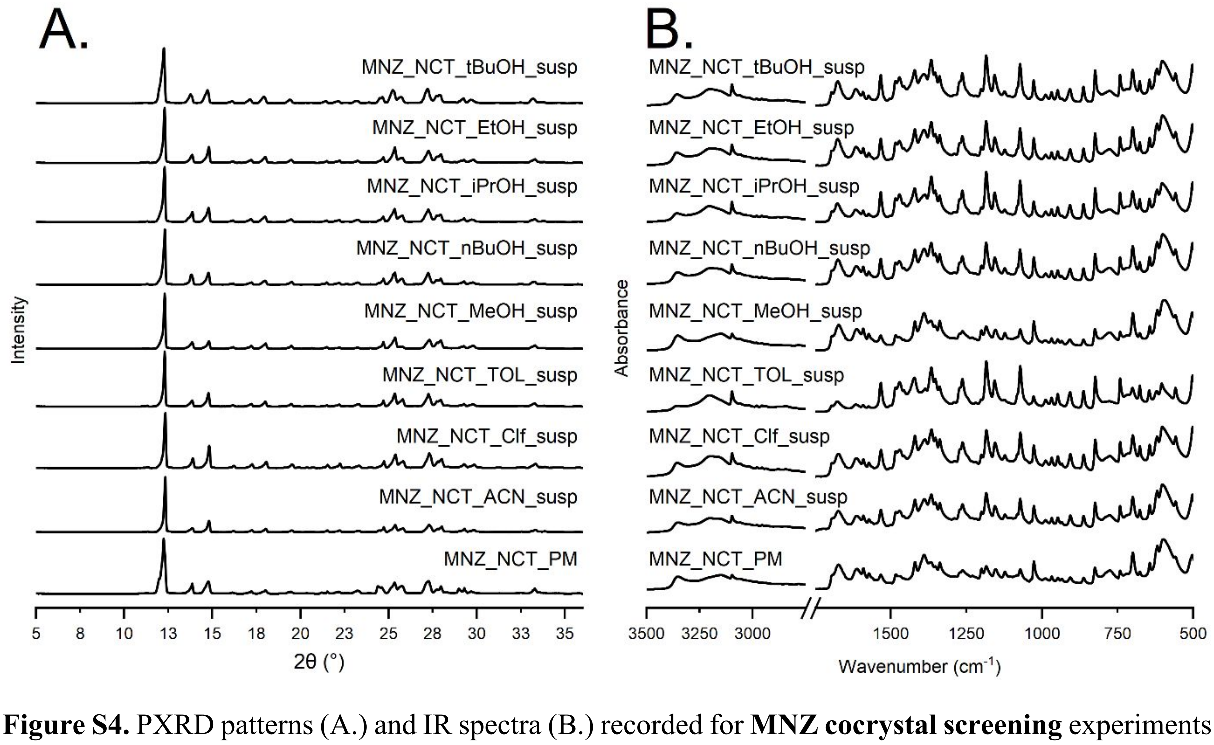

Figure S4. PXRD patterns (A.) and IR spectra (B.) recorded for **MNZ cocrystal screening** experiments using **nicotinamide (NCT)** and the stirred suspension technique.

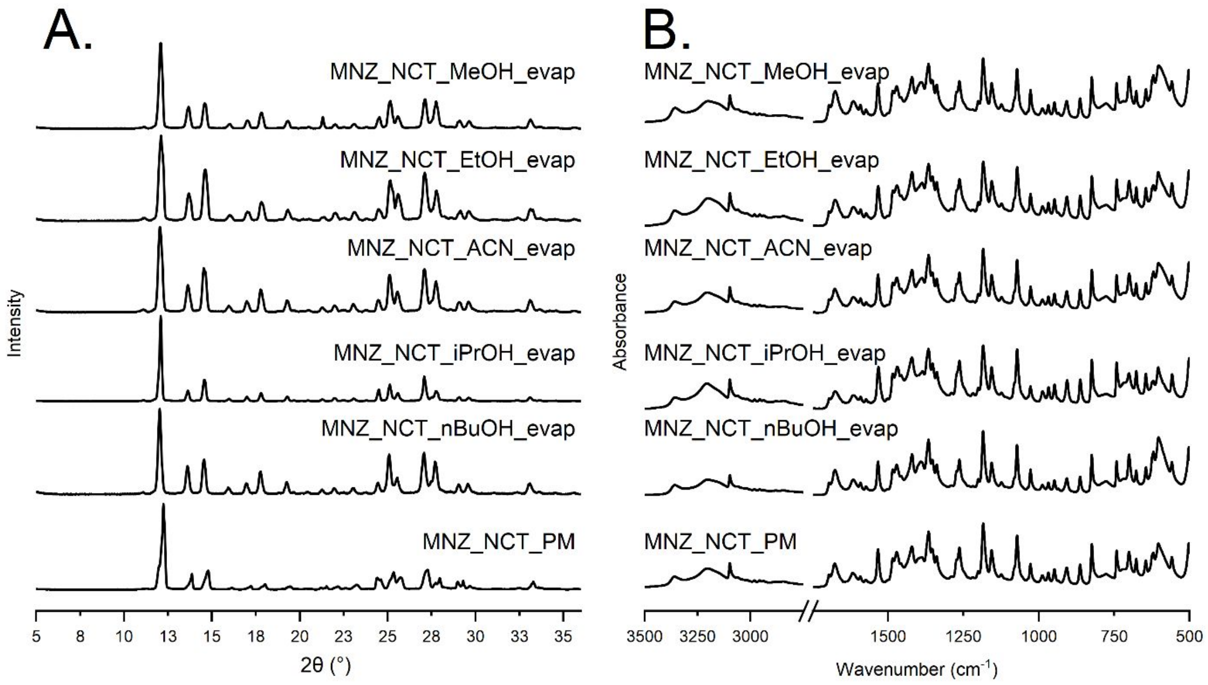

Figure S5. PXRD patterns (A.) and IR spectra (B.) recorded for **MNZ cocrystal screening** experiments using **nicotinamide (NCT)** and the slow evaporation technique.

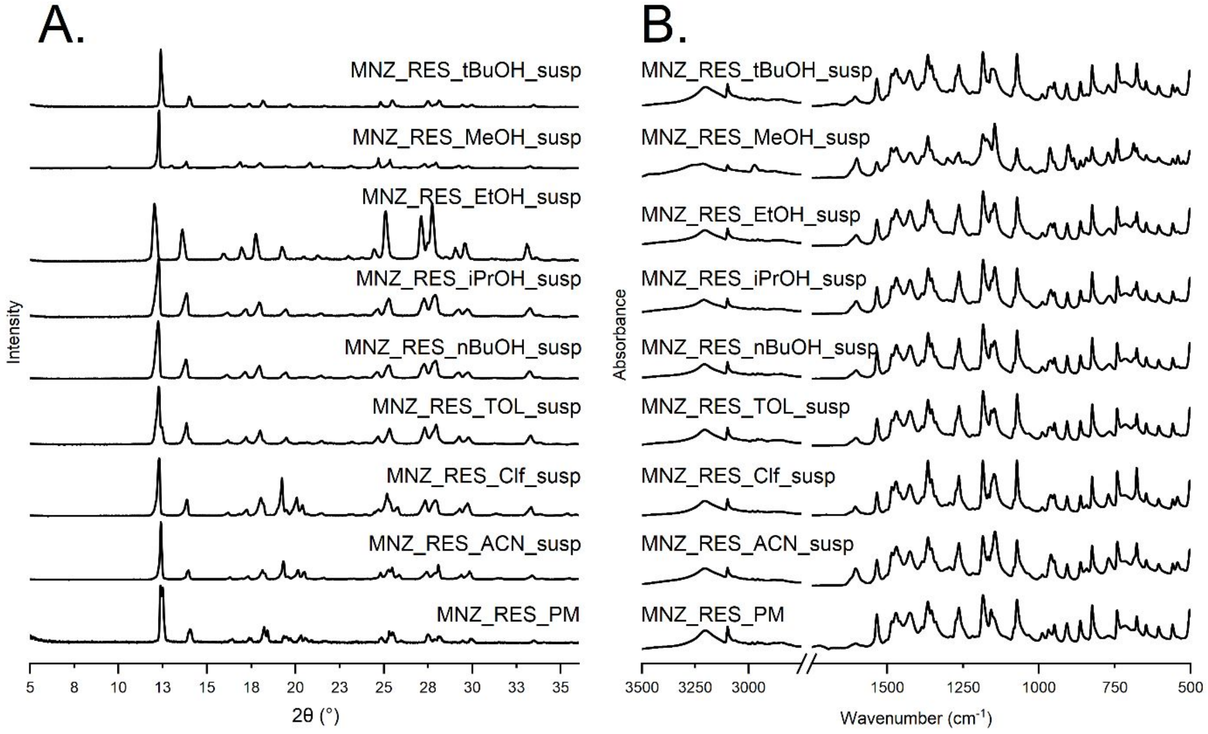

Figure S6. PXRD patterns (A.) and IR spectra (B.) recorded for **MNZ cocrystal screening** experiments using **resorcinol (RES)** and the stirred suspension technique.

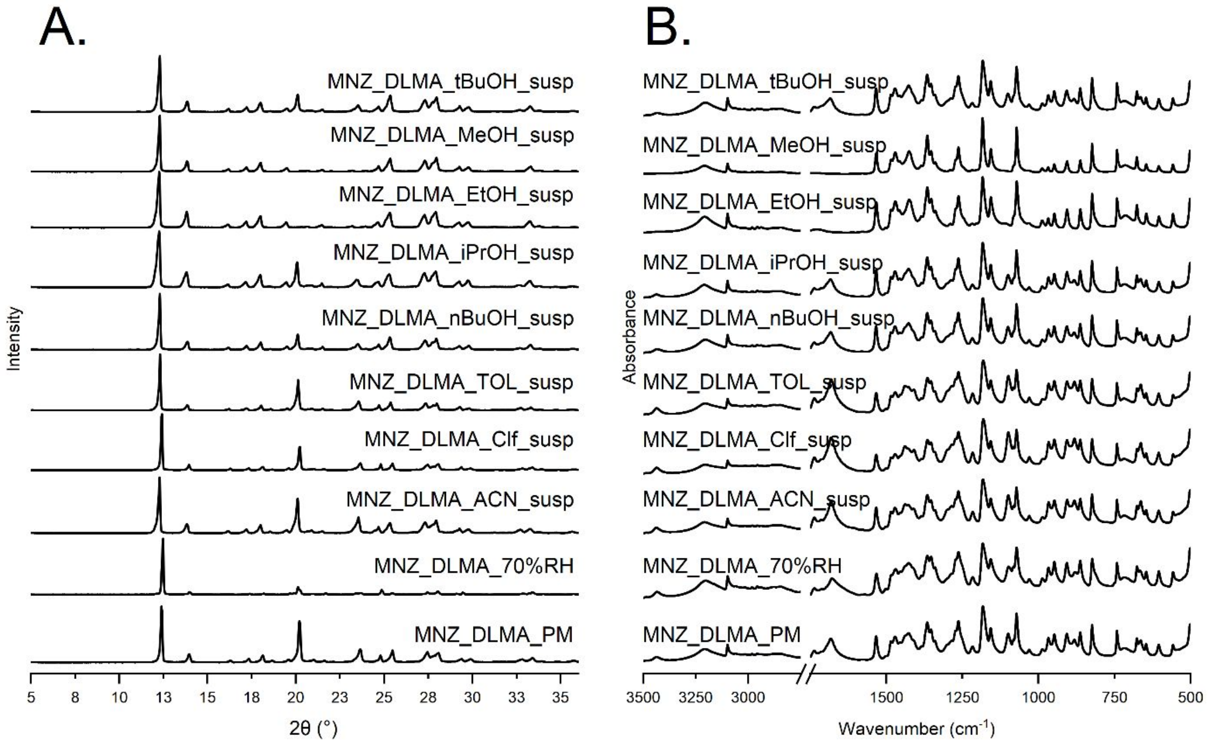

Figure S7. PXRD patterns (A.) and IR spectra (B.) recorded for **MNZ cocrystal screening** experiments using **DL-malic acid (DLMA)** and the stirred suspension technique.

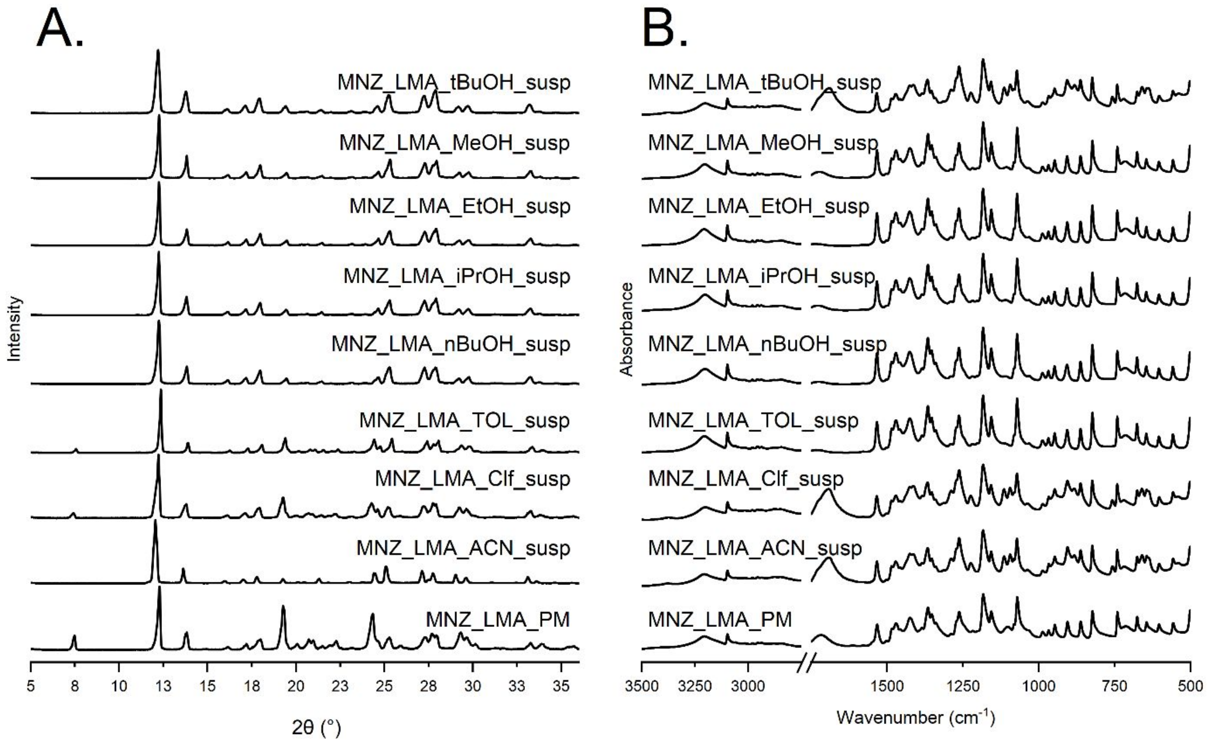

Figure S8. PXRD patterns (A.) and IR spectra (B.) recorded for **MNZ cocrystal screening** experiments using **L-malic acid (LMA)** and the stirred suspension technique.

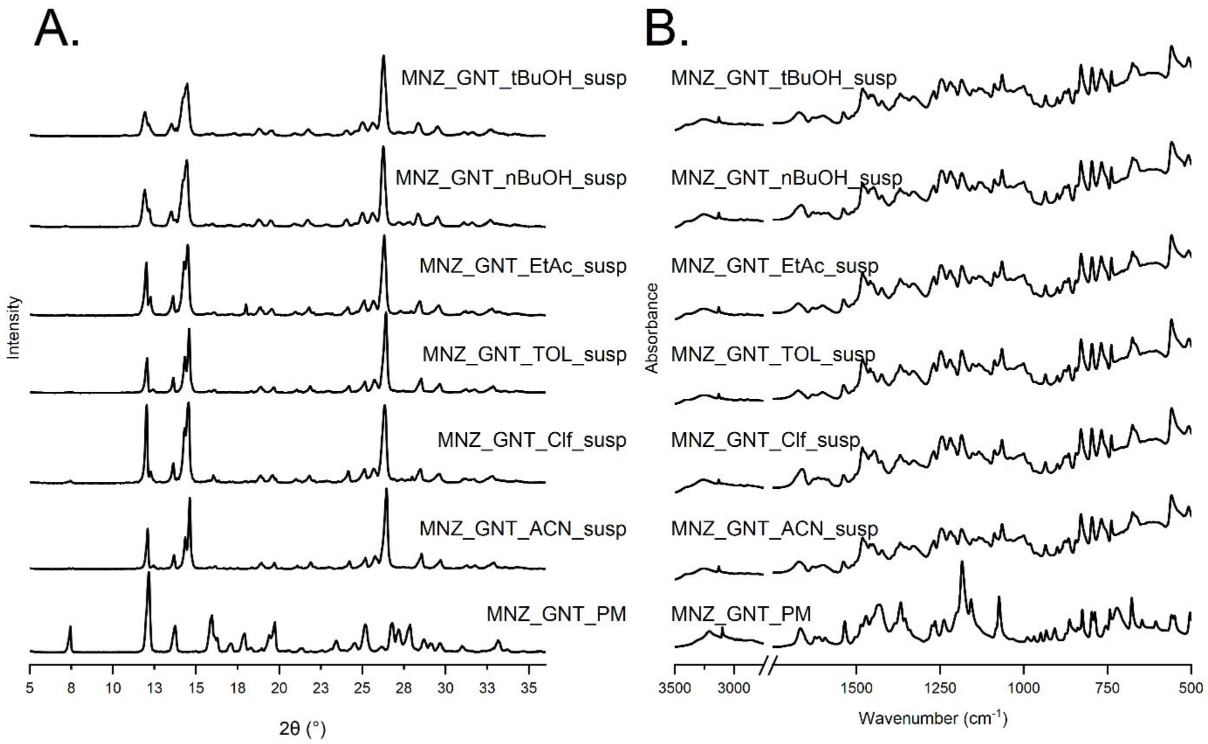

Figure S9. PXRD patterns (A.) and IR spectra (B.) recorded for **MNZ cocrystal screening** experiments using **gentisic acid (GNT)** and the stirred suspension technique.

2.2. Stage 2 cocrystal screening – 3-hydroxbenzoic acid (3-HBA), 4-aminobenzoic acid (4-ABA), 4-hydroxybenzoic acid (4-HBA), benzoic acid (BA), salicylic acid (SAL), *o*-phthalic acid (OPHTA), and gallic acid (GAL)

Table S3. List of experiments and outcomes. *PM* – physical mixture.

Coformer	Technique	Protocol	Solvents used	Outcome
3-HBA (3-hydroxybenzoic acid)	Stirred suspension	200 mg of 1:1 PM was suspended in solvent and stirred at 500 rpm at RT for 1 week	acetonitrile (ACN) chloroform (Clf) toluene (TOL)	PM
4-ABA (4-aminobenzoic acid)				PM
4-HBA (3-hydroxybenzoic acid)				PM
BA (benzoic acid)				PM
SAL (salicylic acid)				PM
OPHTA (*o*-phthalic acid)				PM
**GAL (gallic acid, 3,4,5-trihydroxybenzoic acid)**				ACN – Cocrystal form I° Clf, TOL – Cocrystal form II
3-HBA	Freeze drying	According to the procedure from the main text	H_2O	PM
4-ABA				PM
4-HBA				PM
BA				PM

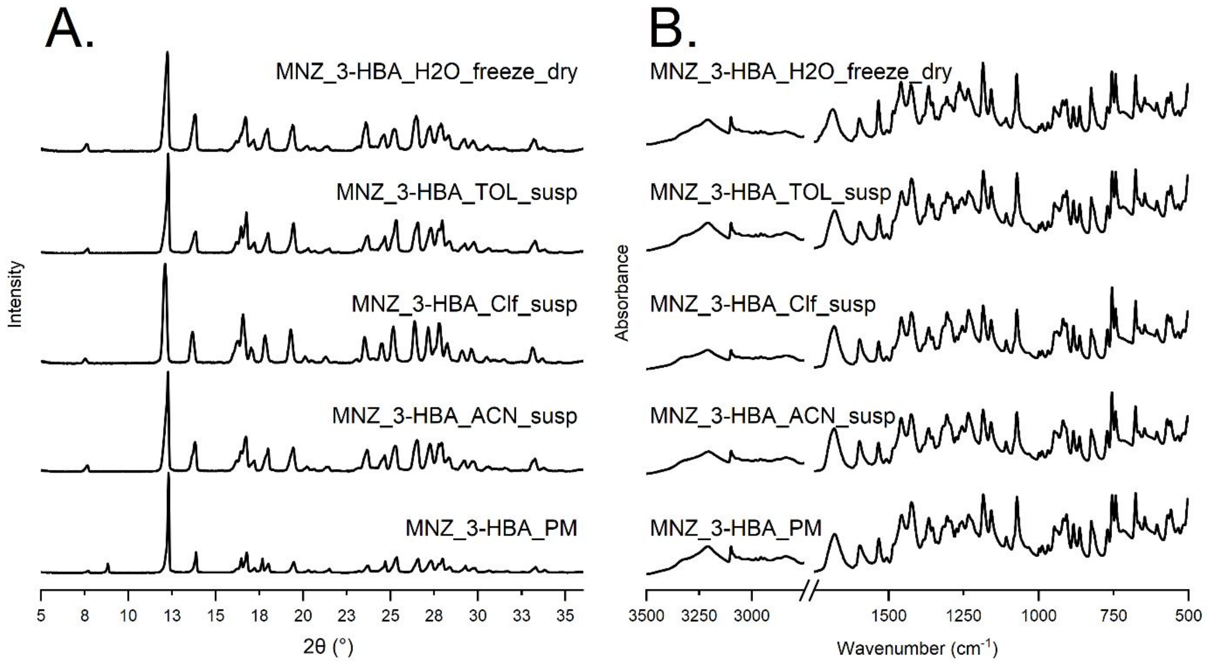

Figure S10. PXRD patterns (A.) and IR spectra (B.) recorded for **MNZ cocrystal screening** experiments using **3-hydroxybenzoic acid (3-HBA)** and the stirred suspension or freeze drying technique.

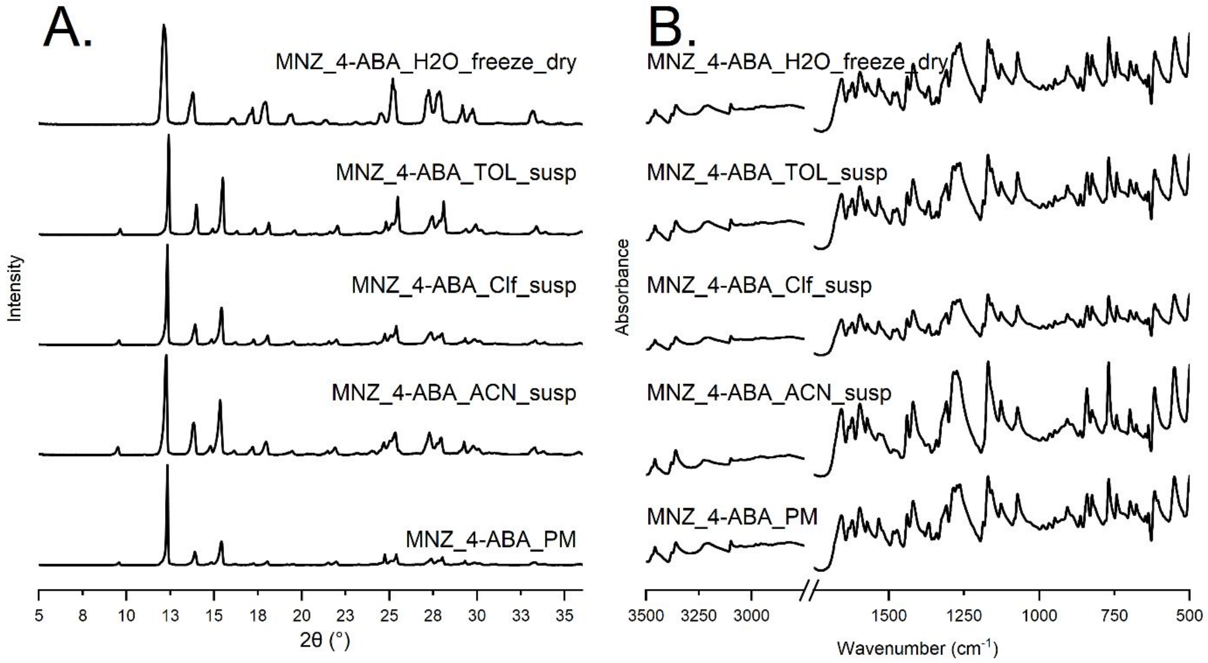

Figure S11. PXRD patterns (A.) and IR spectra (B.) recorded for **MNZ cocrystal screening** experiments using **4-aminobenzoic acid (4-ABA)** and the stirred suspension or freeze drying technique.

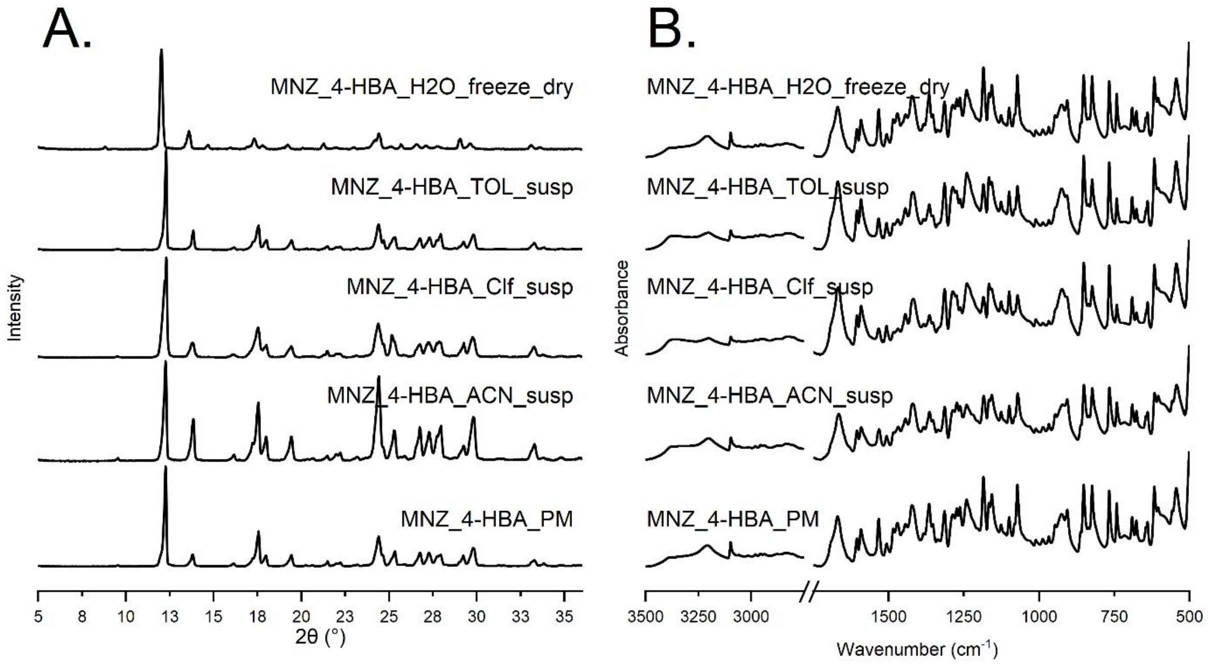

Figure S12. PXRD patterns (A.) and IR spectra (B.) recorded for **MNZ cocrystal screening** experiments using **4-hydroxybenzoic acid (4-HBA)** and the stirred suspension or freeze drying technique.

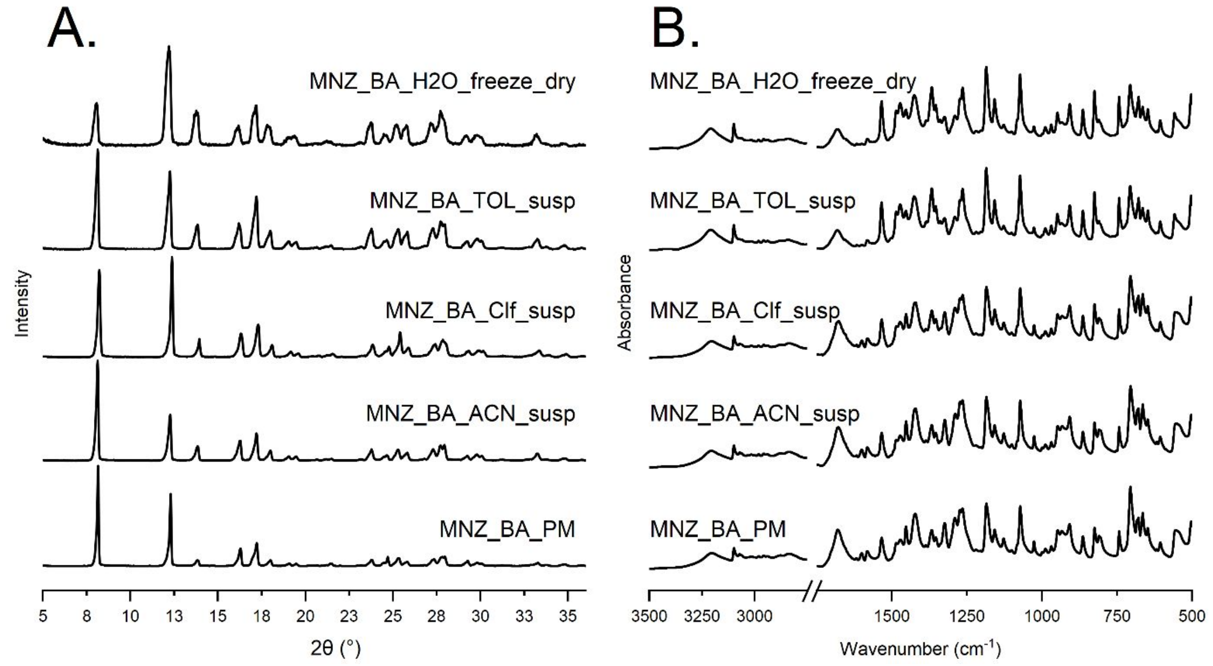

Figure S13. PXRD patterns (A.) and IR spectra (B.) recorded for **MNZ cocrystal screening** experiments using **benzoic acid (BA)** and the stirred suspension or freeze drying technique.

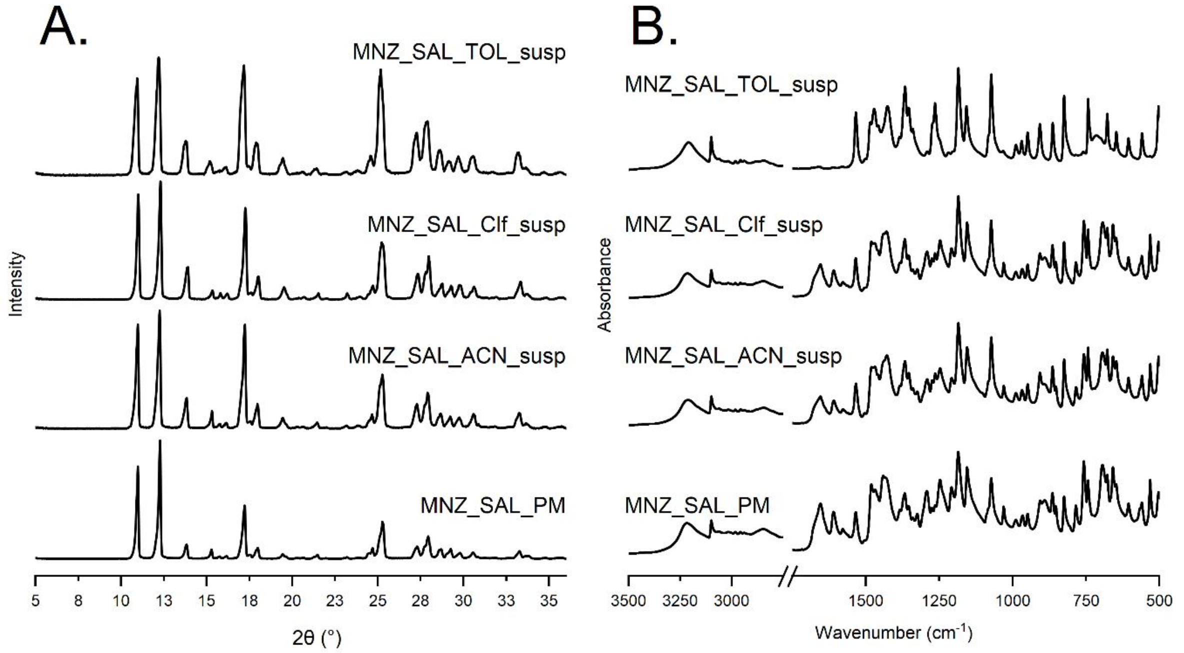

Figure S14. PXRD patterns (A.) and IR spectra (B.) recorded for **MNZ cocrystal screening** experiments using **salicylic acid (SAL)** and the stirred suspension technique.

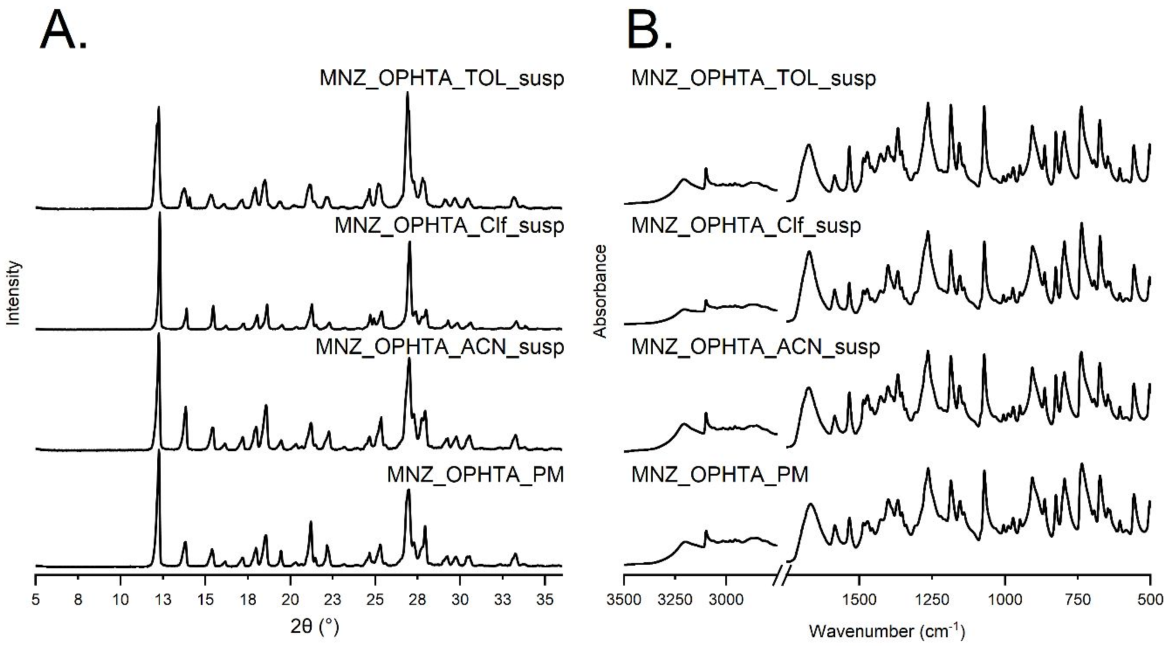

Figure S15. PXRD patterns (A.) and IR spectra (B.) recorded for **MNZ cocrystal screening** experiments using ***o*-phthalic acid (OPHTA)** and the stirred suspension technique.

3. MNZ-GAL cocrystal polymorphs

3.1. Stirred suspension cocrystallization (slurry experiments)

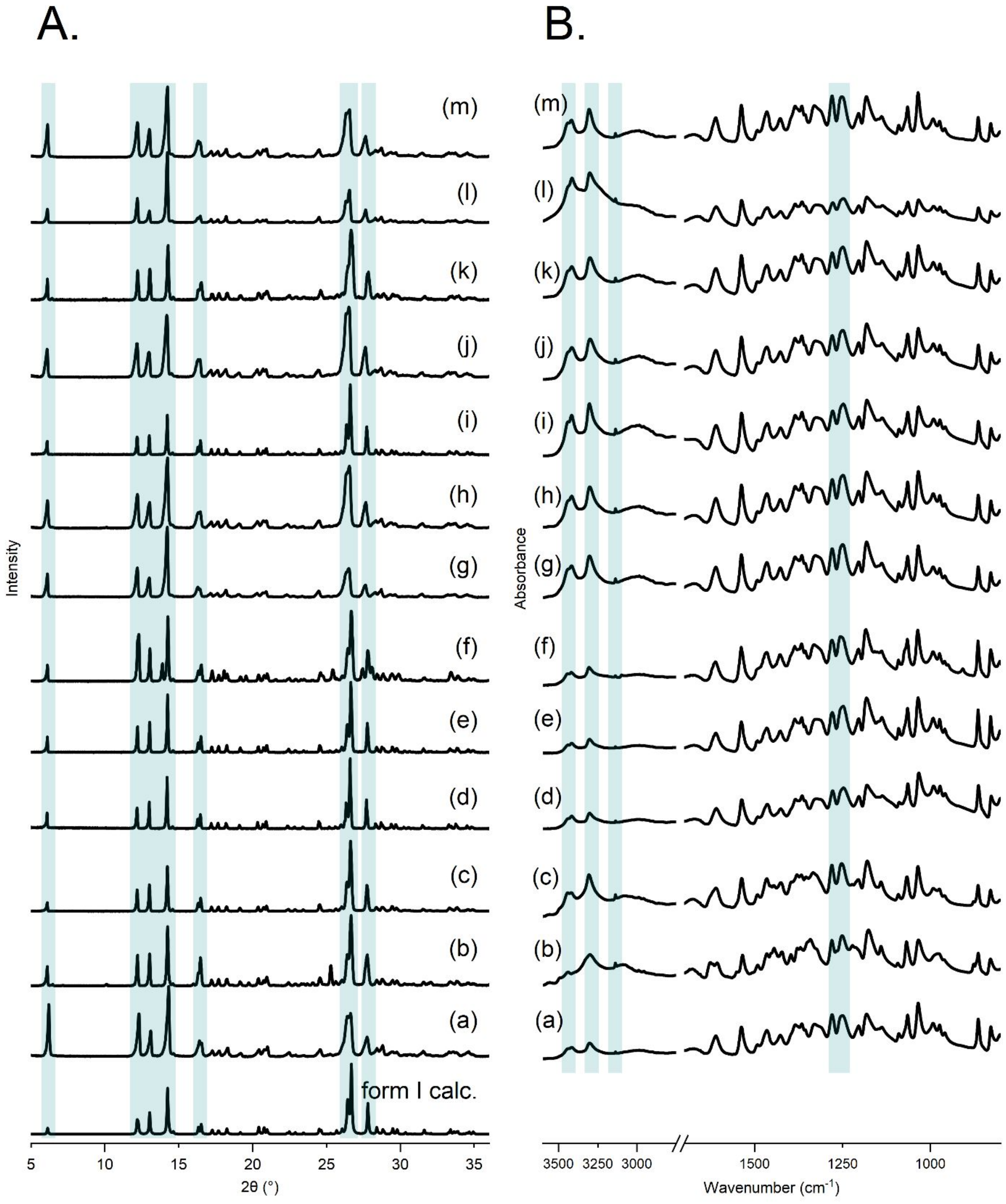

Figure S16. MNZ-GAL cocrystal form I°: PXRD patterns (A.) and IR spectra (B.) obtained by the stirred suspension method using (a) methyl isobutyl ketone, (b) acetone, (c) t-tert-butanol, (d) acetonitrile, (e) *iso*-propanol, (f) *iso*-butanol, (g) *n*-butanol, (h) *n*-propanol, (i) acetic acid, (j) ethanol, (k) methanol, and (m) 2-pentanone. *Form I° calc.* – PXRD pattern calculated from single crystal structure (VOKYEC). Reflection positions and absorbance bands characteristic for the MNZ-GAL cocrystal form I° are marked in green.

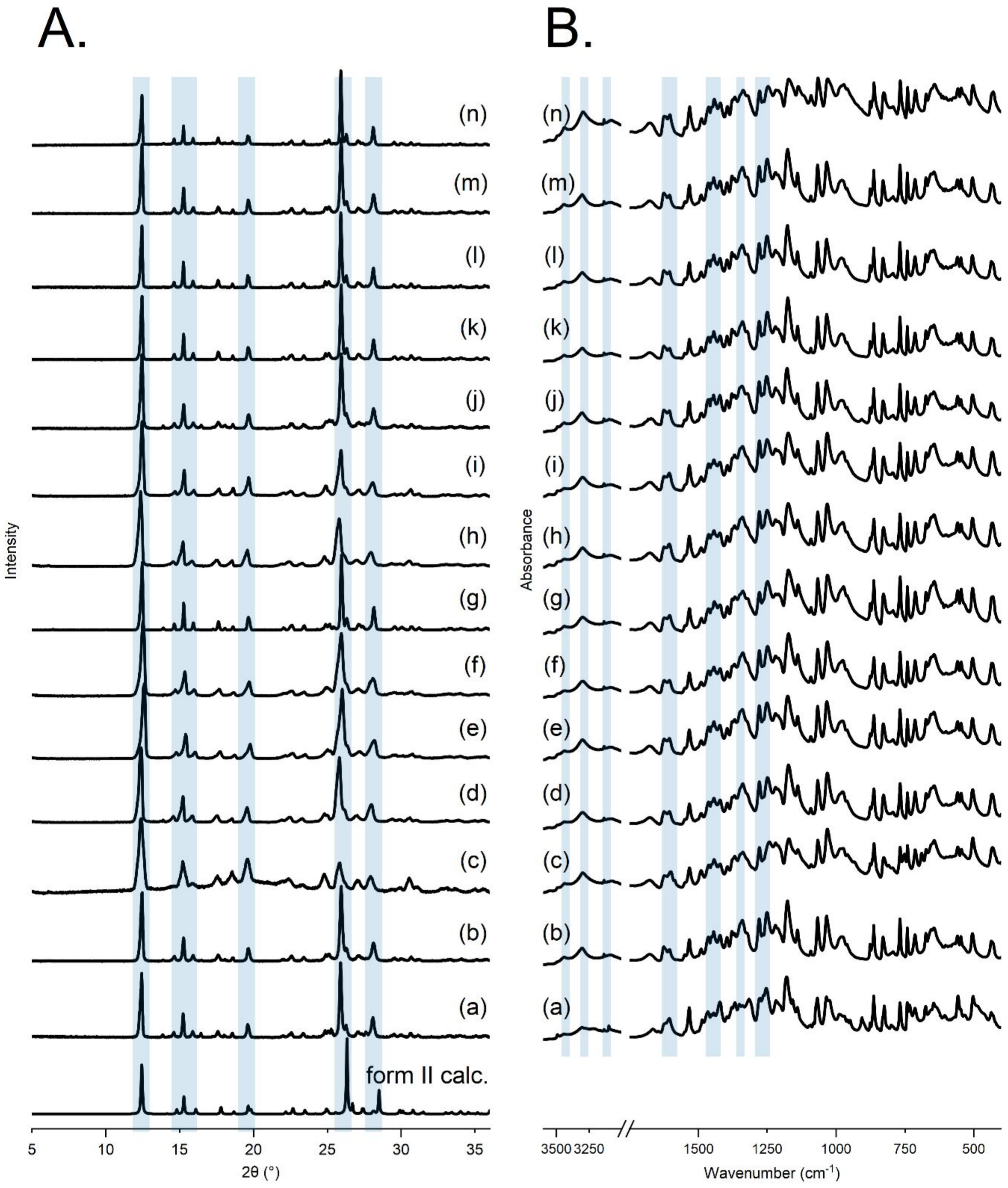

Figure S17. MNZ-GAL cocrystal form II: PXRD patterns (A.) and IR spectra (B.) obtained by the stirred suspension method using (a) hexane, (b) toluene, (c) 2-phenoxypropanol, (d) diethyl ether, (e) isopropyl ether, (f) *n*-buthylmethylether, (g) tetrahydrofuran, (h) ethyl acetate, (i) *n*-butyl acetate, (j) α,α,α-trifluorotoluene, (k) chloroform, (l) dichloromethane, (m) 1,2-dichloroethane, and (n) dimethyl isosorbide. *Form II calc.* – PXRD pattern calculated from single crystal structure (VOKYEC02). Reflection positions and absorbance bands characteristic for the MNZ-GAL cocrystal form II are marked in blue.

3.2. Electrospraying – process parameters for cocrystallization

Table S4. Process parameters for MNZ:GAL electrospraying cocrystallization experiments. *PM* – physical mixture.

Solvent	Content	Feed rate [μL s^{-1}]	Voltage applied [kV]	Needle/collector distance [cm]	Solvent surface tension [mN m^{-1}] at 25°C
THF	100 mg of 1:1 PM in 10 mL	2.00	15.30	10	26.590 (1)
DCM	100 mg of 1:1 PM in 50 mL	2.00	16.50	10	27.15 (2)
BuAc	25 mg of 1:1 PM in 50 mL	2.00	13.50	6	24.6 (2)
EtAc	100 mg of 1:1 PM in 30 mL	2.00	12.70	6	23.340 (3)
ACT	100 mg of 1:1 PM in 10 mL	0.5	12.30	10	22.996 (4)
MeOH	100 mg of 1:1 PM in 10 mL	1.5	16.50	8	21.930 (5)
EtOH	100 mg of 1:1 PM in 15 mL	1.0	12.48	8	21.820 (5)
H_2O	50 mg of 1:1 PM in 50 mL	0.019	20-22	10	72.02 (6)

3.3. Spray drying - process parameters for cocrystallization

Table S5. Process parameters for MNZ-GAL spray drying cocrystallization experiments. *PM* – physical mixture.

Solvent	Content	Solvent boiling point [°C]	Processing (inlet) temp. [°C]	Outlet temp. [°C]	Feeding rate [mL min^{-1}]	Aspirator [%]
EtAc	500 mg of 1:1 PM in 800 mL	77	70	50-52	3.0	100
BuAc	500 mg of 1:1 PM in 1 L	126	120	63-65	3.0	100
ACT	750 mg of 1:1 PM in 400 mL	56.2	50	35	4.0	80
EtOH	500 mg of 1:1 PM in 200 mL	78.5	70	50-52	3.0	80
MeOH	500 mg of 1:1 PM in 200 mL	64.6	60	45-47	3.0	80
H_2O	500 mg of 1:1 PM in 500 mL	100	135	70-75	3.0	100

3.4 Thermal stability of the MNZ-GAL cocrystal polymorphs

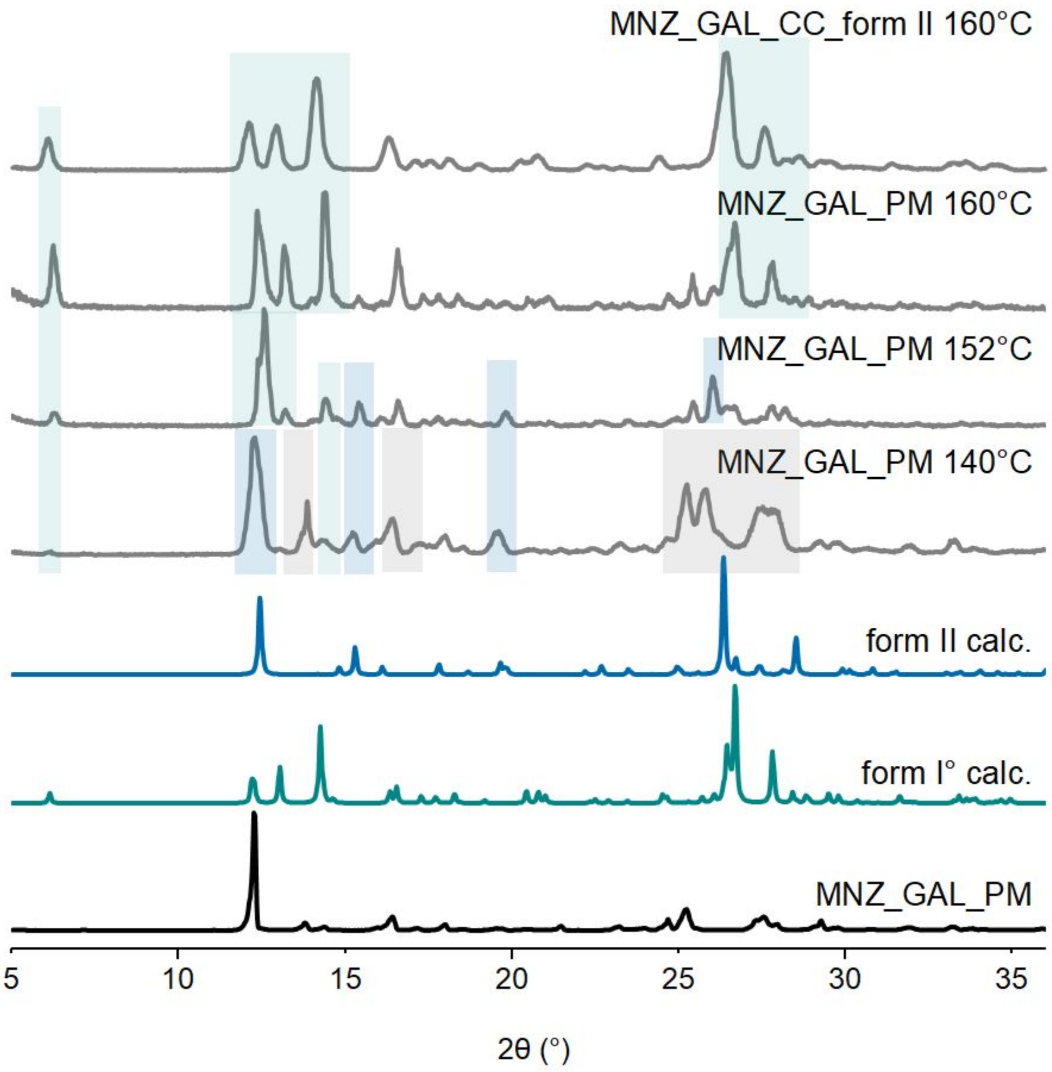

Figure S18. Enlarged PXRD patterns of the MNZ-GAL 1:1 physical mixture and MNZ-GAL cocrystal forms I° and II (calc. – calculated; CSD VOKYEC and VOKYEC01, respectively). The MNZ-GAL 1:1 physical mixture was heated up to 140 °C, 152 °C, and 160 °C. The top PXRD pattern corresponds to the cocrystal form II heated up to 160 °C. The reflexes corresponding to the conversion of the physical mixture or cocrystal form II into cocrystal form I are marked with: gray (starting reagents), blue (cocrystal form II), and green (cocrystal form I°).

4. MNZ-GNT cocrystal polymorphs

4.1. Cocrystallization screen – electrospraying, freeze drying, and spray drying

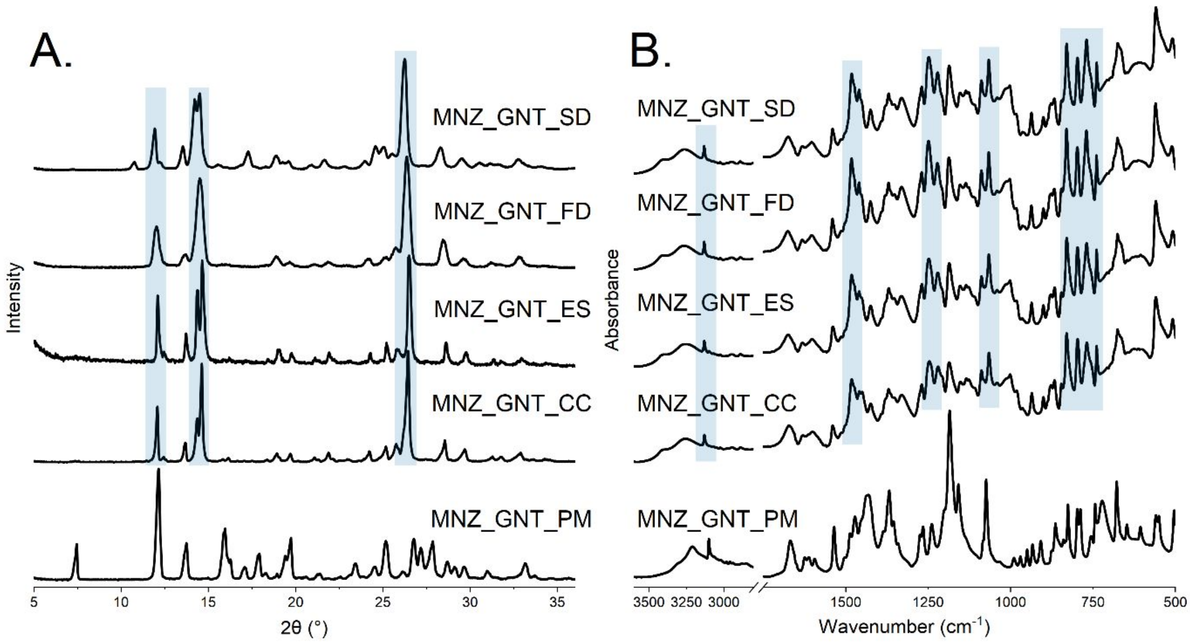

Figure S19. PXRD patterns (A.) and FTIR spectra (B.) of the MNZ-GNT II° 1:1 physical mixture, MNZ-GNT cocrystal form II° obtained experimentally from suspension crystallization in acetonitrile (ACN), by electrospraying (ES), freeze drying (FD), and spray drying (SD) in water. Reflection positions and absorbance bands characteristic for the MNZ-GNT cocrystal form II° are marked in blue.

4.2. MNZ-GNT cocrystal form II° ↔ I transformation

FT-IR spectroscopy

Temperature controlled FTIR spectra were recorded using a diamond ATR crystal (PIKE GaldiATR, Madison, US) on a Bruker Vertex 70 FTIR spectrometer (Bruker Analytische Messtechnik GmbH, Germany). The spectra were recorded from 4000 to 400 cm^{-1}, with 32 scans recorded per spectrum at a 2 cm^{-1} resolution. Temperature controller (PIKE, Madison US) was used to set the experiment conditions. Both heating and cooling rates were set to 2 °C min^{-1} using the TempPRO software (PIKE, Madison, US). Upon reaching the programmed temperature when heating, isothermal conditions were kept for 5 min per step; for the cooling 10 min steps were implemented.

The temperature dependent IR measurements revealed that the two cocrystals (form I and II°) exhibit a high structural resemblance. Furthermore, based on the vibrational bands it could be concluded that the same protonation state is present in the two solid-state forms (Figure S19).

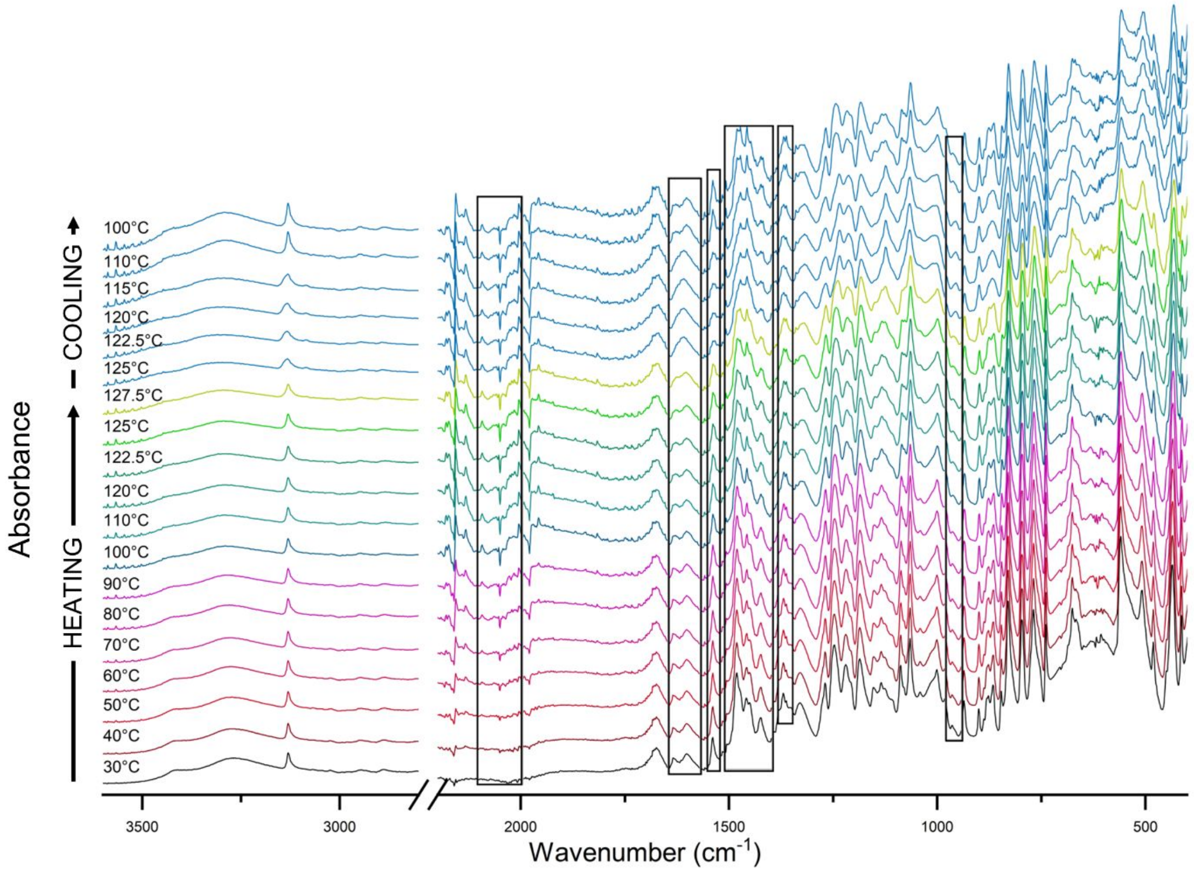

Figure S20. Temperature-dependent IR spectra of the MNZ-GNT cocrystals. Black rectangles mark spectral regions exhibiting changes upon heating. The sample was heated from 30 °C to 127.5 °C and then cooled to 100 °C as indicated by arrows.

PXRD measurements

The temperature-dependent PXRD measurements clearly indicate the presence of different solid-state forms (Figure S21).

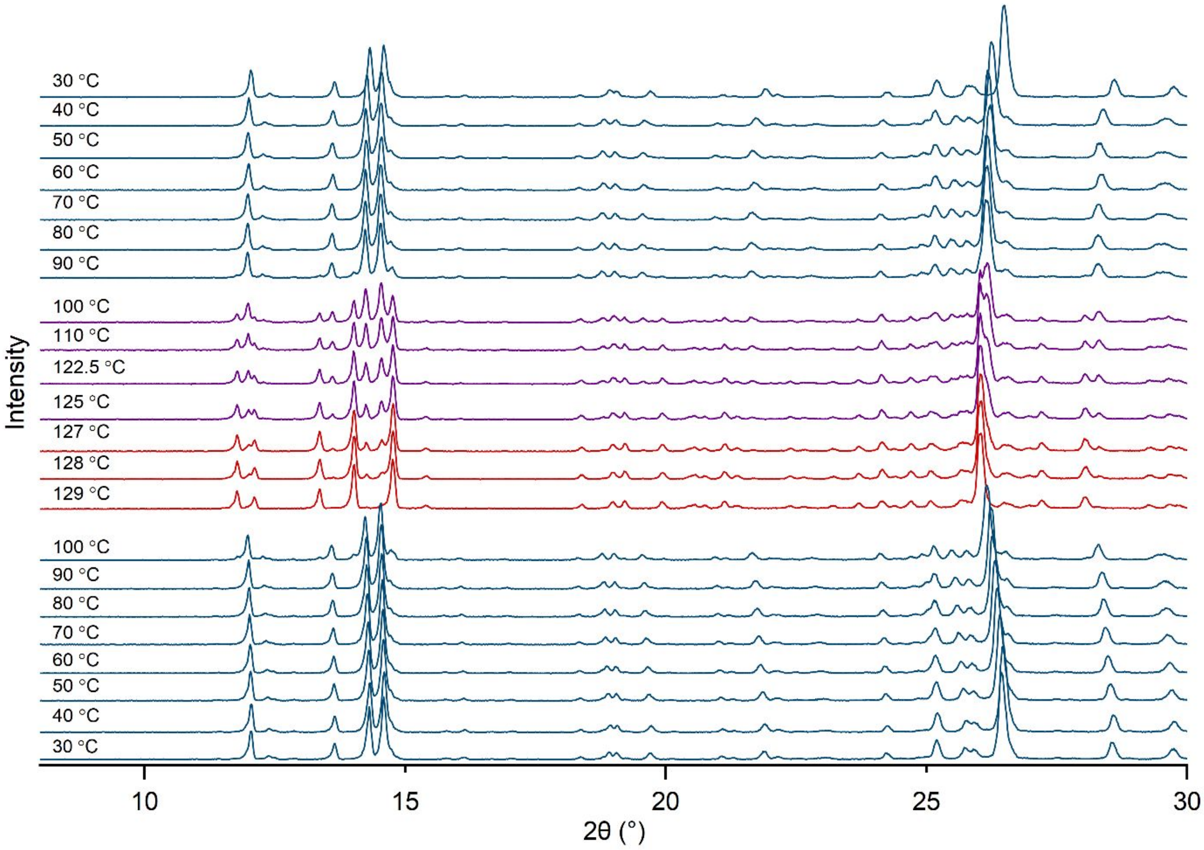

Figure S21. Temperature-dependent PXRD diffractograms of the MNZ-GNT cocrystals. The top PXRD pattern is the start of the experiment (heating from 30 °C to 127 °C and then cooling back to 30 °C). The RT-stable form is presented in blue and the high temperature form is presented in red. Purple represents the phase transformation part of the heating experiment.

4.3. MNZ-GNT cocrystal form I structure

The crystal structure of the high temperature form was solved from PXRD data measured at 123 °C (Figure S22). Electronic structure calculations were used to support the structure solution.

Using CASTEP v20.11 (7) and the PBE generalized gradient approximation (GGA) exchange-correlation density functional (8), the cocrystal structure was optimized. Ultra-soft pseudopotentials (9) were employed along with the MBD* dispersion correction (10). The selection of k-points ensured a maximum spacing of $2\pi \cdot 0.07$ Å^{-1}, while a basis set cut-off of

780 eV was applied. The convergence criteria were as follows: <2x10^{-5} eV per atom, atomic displacements <1x10^{-3} Å, maximum forces <5x10^{-2} eV Å^{-1}, and maximum stresses <0.1 GPa.

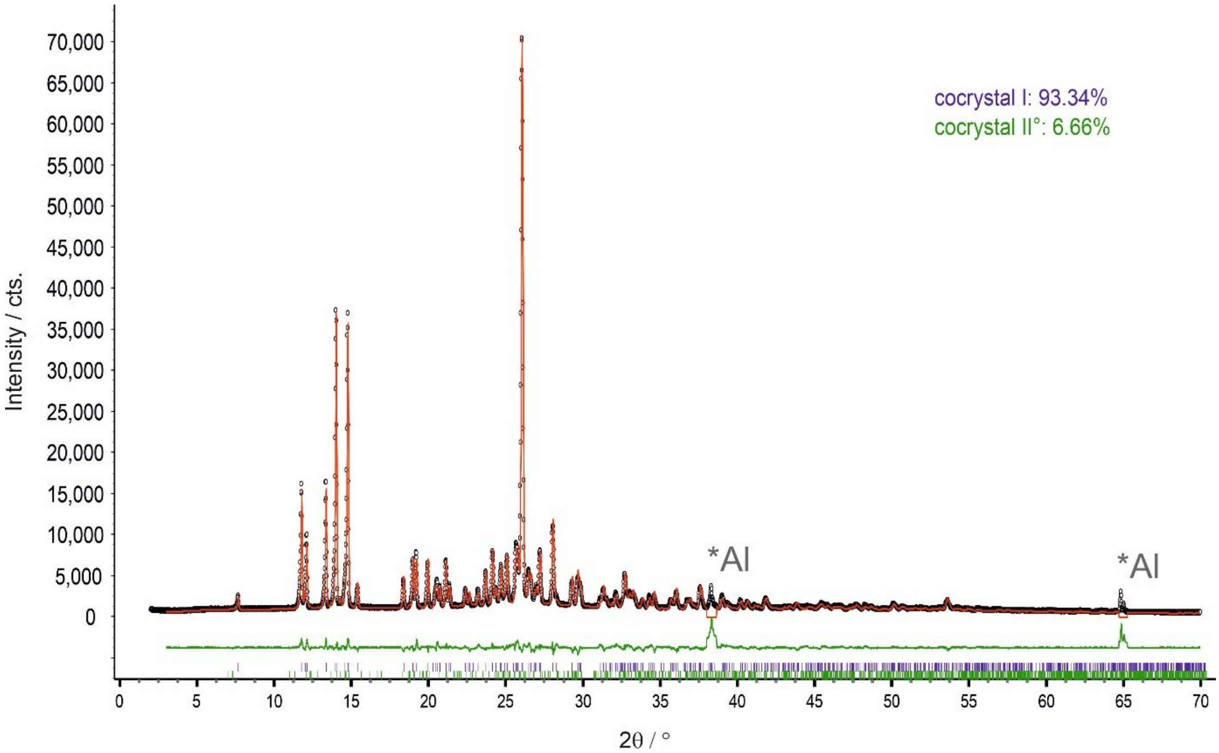

Figure S22. Powder X-ray diffraction pattern and Rietveld fit (rigid body) of MNZ-GNT form I: Observed (black points), calculated (red line), and difference profiles (green). Blue tick marks denote the form I peak positions and green tick marks the form II° peak positions (as impurity present, 6.7%). The sample was measured on an Al foil. Key Al reflections are highlighted (*Al) and were excluded from the refinement.

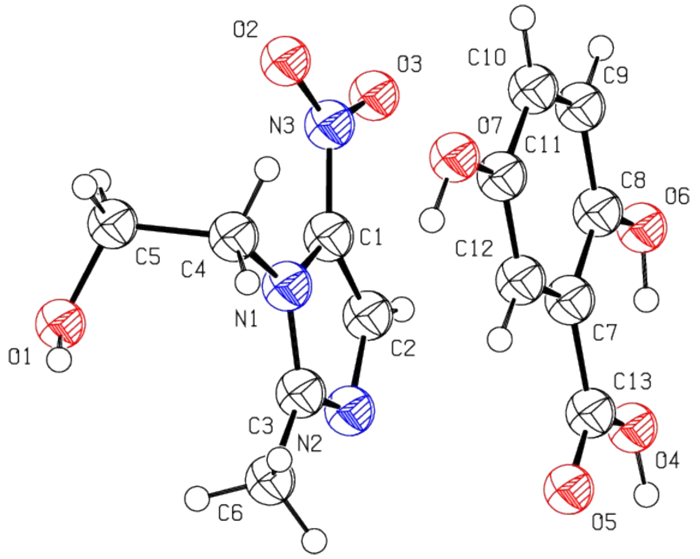

Figure S23. A view of the structure of MNZ-GNT form I (396 K), showing the atom-labelling scheme. Displacement ellipsoids are drawn at the 50% probability level.

Experimental tables for the form I structure are compiled together with the form II° results (see next section).

4.4. MNZ-GNT cocrystal form II° structure

Single crystals of the MNZ-GNT cocrystal form II° were obtained by slow evaporation from either water, toluene or acetonitrile. A physical mixture consisting of 105.2 mg of MNZ and 94.8 mg GNT (a 1:1 molar ratio) was weighted and ground using mortar and pestle. The powder was then placed in a glass vial and 2−3 mL of solvent were added. The mixture was stirred until the solids had completely dissolved. The solution was then left to slowly evaporate at RT, and crystals formed after one day. A suitable single crystal, obtained from water, was selected for structure determination.

The X-ray single crystal measurement was performed at RT (295 K) and LT (100 K), confirming that upon cooling no phase transformation occurred.

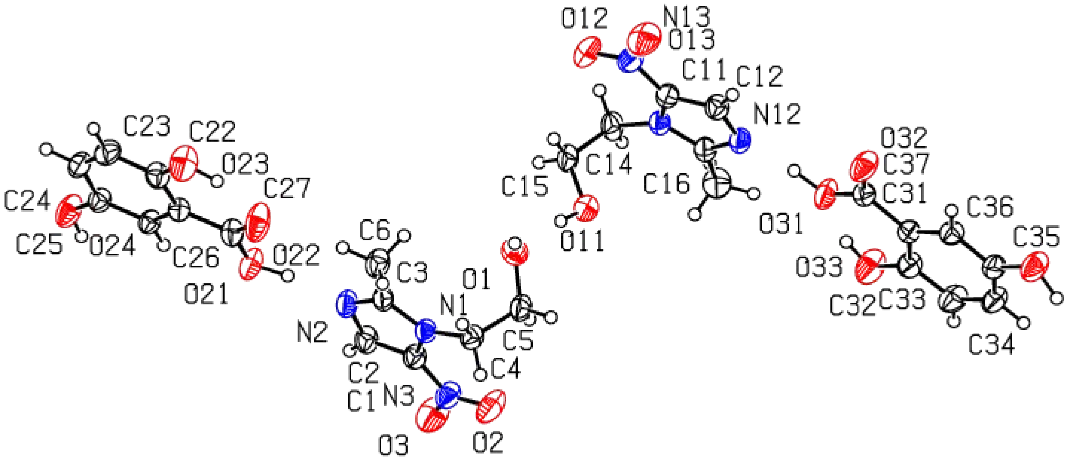

Figure S24. A view of the structure of MNZ-GNT form II° (295 K), showing the atom-labelling scheme. Displacement ellipsoids are drawn at the 50% probability level.

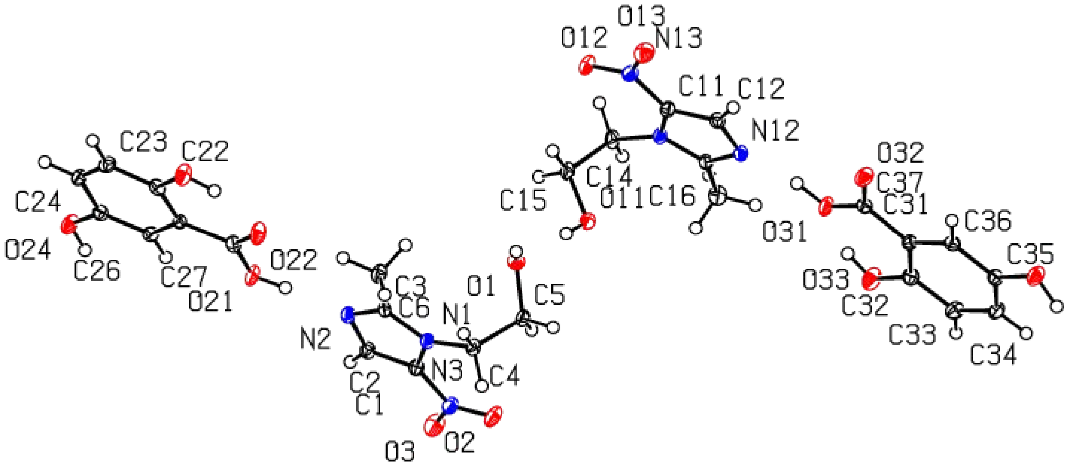

Figure S25. A view of the structure of MNZ-GNT form II° (100 K), showing the atom-labelling scheme. Displacement ellipsoids are drawn at the 50% probability level.

Table S6. Experimental details for the MNZ-GNT cocrystals.

Crystal data	**MNZ-GNT form I**	**MNZ-GNT form II°**	**MNZ-GNT form II°**
Chemical formula	$C_6H_9N_3O_3 \cdot C_7H_6O_4$	$C_6H_9N_3O_3 \cdot C_7H_6O_4$	$C_6H_9N_3O_3 \cdot C_7H_6O_4$
Molar mass	325.28	325.28	325.28
Crystal system	Triclinic	Triclinic	Triclinic
Space group	*P*-1	*P*-1	*P*-1
a (Å)	8.40063 (14)	8.3901 (3)	8.3289(3)
b (Å)	8.60425 (15)	14.3391 (5)	14.0687(4)
c (Å)	11.5573 (2)	14.3022 (4)	14.2777(5)
α [°]	84.1056 (14)	61.317 (5)	61.261(3)
β [°]	89.0926 (12)	74.655 (4)	74.698(4)
γ [°]	61.2686 (15)	87.287 (4)	86.607(3)
Volume (Å^3)	728.11 (3)	1449.22 (11)	1410.39(9)
Z	2	4	4
Radiation type	Cu $K\alpha_{1.2}$	Mo Kα	Mo Kα
μ (mm−1)	1.05	0.12	0.13
Crystal size (mm)		0.28 × 0.26 × 0.22	0.28 × 0.26 × 0.22
Data collection			
Diffractometer	Panalytical XPert PRO	Xcalibur, Atlas	Xcalibur, Atlas
Absorption correction		Multi-scan	Multi-scan
Temperature (K)	396	295	100
Data collection mode	Transmission		
2θ values (°)	$2\theta_{min}$ = 2.001 $2\theta_{max}$ = 69.985 $2\theta_{step}$ = 0.013		
T_{min}, T_{max}		0.981, 1.000	0.982, 1.000
No. of measured, independent and observed [I > 2σ(I)] reflections		98618 6625 5142	36780 6465 5183
R_{int}		0.029	0.030
(sin θ/λ)$_{max}$ (Å^{-1})		0.650	0.650
Refinement			
$R[F^2 > 2\sigma(F^2)]$, $wR(F^2)$, S		0.041 0.120 0.98	0.037 0.091 1.00
R factors and goodness of fit	R_p = 0.043 R_{wp} = 0.063		

	R_{exp} = 0.025<br>R_{Bragg} = 0.036<br>χ^2 = 6.202		
No. of reflections		6625	6465
No. of parameters		441	441
H-atom treatment		mixture of independent and constrained refinement	mixture of independent and constrained refinement
$\Delta\rho_{max}$, $\Delta\rho_{min}$ (e Å^{-3})		0.30<br>-0.22	0.40<br>−0.31

5. Pairwise intermolecular energy calculations

5.1. Metronidazole

Table S7. Pairwise intermolecular interactions[a] seen in **MNZ**.

No.	Interaction	Symop.	*n*	R Å	E_E	E_P	E_R kJ mol^{-1}	E_D	E_{tot}
1	π···π & C–H···O	-x, -y, -z	1	3.92	–41.4	–4.6	–48.6	66.1	–48.6
2	O–H···N	x, -y+1/2, z+1/2	2	7.41	–68.5	–17.6	–17.2	89.6	–45.0
3	π···π	-x, -y, -z	1	4.24	–14.1	–4.3	–46.2	28.9	–40.6
4		-x, y+1/2, -z+1/2	2	6.76	–6.3	–3.9	–14.0	11.2	–14.8
5		x, -y+1/2, z+1/2	2	7.89	–9.4	–1.9	–6.5	9.9	–10.8
6		-x, y+1/2, -z+1/2	2	7.11	–3.2	–1.8	–11.4	6.3	–10.7
7		-x, -y, -z	1	8.96	–5.4	–0.8	–6.2	3.9	–9.3
8		x, y, z	2	8.49	–5.5	–1.9	–6.9	6.5	–9.2
9		-x, -y, -z	1	9.10	–1.9	–0.3	–2.0	0	–3.9

[a] electrostatic (E_E), polarization (E_P), dispersion (E_D), and exchange-repulsion (E_R). $E_{tot} = k_E E_E + k_P E_P + k_D E_D + k_R E_R$, with k being scale factors. (11)

5.2. Gallic acid

Table S8. Pairwise intermolecular interactions[a] seen in **GAL form I**.

No.	Interaction	Symop.	*n/2*	R Å	E_E	E_P	E_R kJ mol^{-1}	E_D	E_{tot}
1	2x O_{acid}–H···O_{acid}	-x, -y, -z	0.5	8.80	–164.4	–40.2	–13.0	219.8	–79.3
2	2x O_{acid}–H···O_{acid}	-x, -y, -z	0.5	8.82	–154.7	–34.0	–12.7	197.5	–77.7
3	O–H···O_{acid}	-	1	6.82	–53.6	–2.1	–10.6	49.4	–36.9
4	π···π	-x, -y, -z	0.5	3.73	–11.2	–2.1	–42.3	26.2	–34.1
5	π···π	-x, -y, -z	0.5	3.88	–9.2	–2.1	–36.5	18.2	–31.7
6	2x O–H···O	-x, -y, -z	0.5	8.07	–46.5	–10.5	–13.4	62.7	–29.9
7	O–H···O	-	1	6.94	–48.1	–6.2	–12.0	58.4	–29.8
8	O–H···O	-	1	6.2	–35.5	–6.5	–18.7	50.6	–27.4

9	O–H···O	-	1	7.21	–30.2	–9.4	–12.2	43.6	–22.6
10	π···π	-x, -y, -z	0.5	3.49	1.5	–2.1	–49.8	37.2	–20.3
11	O–H···O	-	1	6.97	–38.6	–0.5	–13.4	56.2	–18.1
12		-x, -y, -z	0.5	4.98	2.3	–2.1	–20.7	7.2	–12.7
13		x, y, z	1	8.22	–6.8	–0.6	–6.2	1.6	–12.1
14		x, y, z	1	7.21	–3.7	–0.6	–9.3	1.8	–11.4
15		-	1	6.23	–3.3	–0.6	–11.3	7.5	–9.1
16		-	1	8.5	–4	–0.5	–1.8	0	–6.1
17		-x, -y, -z	0.5	9.20	1.1	0	–4.5	1.6	–1.8
18		-	1	8.53	1.5	–0.4	–1.5	0	–0.1
19		-	1	7.92	4.5	–0.6	–3.8	0.4	1.2
20		-	1	8.05	9.3	–1.1	–3.4	0.4	6.3

[a] electrostatic (E_E), polarization (E_P), dispersion (E_D), and exchange-repulsion (E_R). $E_{tot} = k_E\ E_E + k_P\ E_P + k_D\ E_D + k_R\ E_R$, with k being scale factors. (11)

Table S9. Pairwise intermolecular interactions[a] seen in **GAL form II°**.

No.	Interaction	Symop.	*n*	R<br>Å	E_E	E_P	E_R<br>kJ mol⁻¹	E_D	E_{tot}
1	2x O_{acid}–H···O_{acid}	-x, -y, -z	1	8.87	–154.5	–35.6	–12.7	191.9	–82.2
2	O–H···O_{acid}	x, -y, z+1/2	2	5.67	–70	–17.0	–20.1	87.1	–50.3
3	O–H···O	-x+1/2, y+1/2, -z+1/2	2	7.50	–32.8	–6.8	–10.5	43.9	–21.7
4	H···π	-x, -y, -z	1	6.57	–9.1	–1.4	–17.0	8.1	–20.5
5	O–H···O	x, -y, z+1/2	2	6.63	–9.1	–1.5	–12.8	9.6	–16.0
6	π···π	-x+1/2, -y+1/2, -z	1	6.57	–5.1	–0.9	–12.9	3.3	–15.3
7		x, y, z	2	4.86	2.7	–2.1	–28.6	15.4	–14.1
8		-x+1/2, -y+1/2, -z	1	8.86	–9.0	–0.9	–7.0	10.2	–10.0
9		-x, y, -z+1/2	2	7.33	–2.2	–0.4	–6.3	1.7	–7.1
10		-x, y, -z+1/2	2	8.80	9.2	–1.3	–2.8	0.3	6.5

[a] electrostatic (E_E), polarization (E_P), dispersion (E_D), and exchange-repulsion (E_R). $E_{tot} = k_E\,E_E + k_P\,E_P + k_D\,E_D + k_R\,E_R$, with *k* being scale factors. (11)

Table S10. Pairwise intermolecular interactions[a] seen in **GAL form III**.

No.	Interaction	Symop.	*n*	R<br>Å	E_E	E_P	E_R<br>kJ mol⁻¹	E_D	E_{tot}
1	2x O_{acid}–H···O_{acid}	-x, -y, -z	1	8.86	–158.8	–36	–12.8	203.9	–79.7
2	O–H···O_{acid}	x, y, z	2	7.28	–69.7	–14.4	–12.4	78.5	–46.7
3	O–H···O	-x, y+1/2, -z+1/2	2	7.52	–39.2	–8.1	–10.7	54.9	–22.8
4	H···π	-x, -y, -z	1	6.77	–8.3	–1.2	–14.4	5.3	–19
5	π···π	x, y, z	2	4.81	1.8	–2.1	–28.6	15.2	–15.2
6		-x, y+1/2, -z+1/2	2	6.94	–4.9	–1.5	–12.5	8.0	–12.2
7		x, y, z	2	5.47	2.4	–2.2	–17.4	6.9	–9.9
8		-x, -y, -z	1	9.38	–3.1	–0.2	–1.8	0	–5.0
9		-x, -y, -z	1	7.44	16.1	–2.9	–10.8	7.6	10.1

[a] electrostatic (E_E), polarization (E_P), dispersion (E_D), and exchange-repulsion (E_R). $E_{tot} = k_E\,E_E + k_P\,E_P + k_D\,E_D + k_R\,E_R$, with *k* being scale factors. (11)

5.3. Gentisic acid

Table S11. Pairwise intermolecular interactions[a] seen in **GNT form I**.

No.	Interaction	Symop.	*n/2*	R<br>Å	E_E	E_P	E_R<br>kJ mol⁻¹	E_D	E_{tot}
1	2x O_{acid}–H···O_{acid}	-	1	7.62	–144.0	–33.0	–13.5	188.5	–72.0
2	O–H···O	-	1	7.39	–47.6	–10.8	–14	62.2	–32.1
3	O–H···O	-	1	8.55	–45.8	–9.6	–8.5	62	–24.7
4	π···π	-	1	5.47	–7.6	–1.6	–20.0	10.8	–19.9
5	π···π	x, y, z	1	4.83	–3.8	–1.6	–26.4	15.8	–18.4
6	π···π	x, y, z	1	4.83	–3.1	–1.3	–26.6	16.6	–17.1
7		-	1	7.87	–9.1	–1.5	–6.9	7.3	–12.2

8		x, -y, z+1/2	1	5.75	–3.6	–1.2	–11.1	3.7	–12.1
9		-	1	6.6	–3.9	–0.8	–11.9	7.0	–10.8
10		x, -y, z+1/2	1	7.37	–4.9	–0.8	–10.0	9.7	–8.5
11		x, -y, z+1/2	1	5.77	0.9	–0.6	–10.2	2.6	–6.8
12		x, -y, z+1/2	1	7.4	–2.7	–0.9	–9.8	8.5	–6.7
13		-	1	7.76	–6.1	–1.5	–7.1	11.8	–6.4
14		-	1	6.73	2.4	–0.9	–10.8	6.1	–3.8

[a] electrostatic (E_E), polarization (E_P), dispersion (E_D), and exchange-repulsion (E_R). $E_{tot} = k_E E_E + k_P E_P + k_D E_D + k_R E_R$, with k being scale factors. (11)

Table S12. Pairwise intermolecular interactions[a] seen in **GNT form II°**.

No.	Interaction	Symop.	*n*	R Å	E_E	E_P	E_R kJ mol^{-1}	E_D	E_{tot}
1	2x O_{acid}–H···O_{acid}	-x, -y, -z	1	7.65	–128.4	–27.7	–13.0	158.0	–70.0
2	O–H···O	-x+1/2, y+1/2, -z+1/2	2	7.82	–47.6	–10.1	–10.9	62.2	–28.8
3	O–H···O	-x, -y, -z	1	5.61	–8.7	–1.6	–18.5	9.0	–21.0
4	π···π	x, y, z	2	4.76	–3.6	–1.2	–28.5	20.0	–17.2
5	π···π	x, y, z	2	5.67	–3.4	–1.2	–15.8	7.4	–13.6
6	π···π	x, y, z	2	7.4	–6.8	–0.9	–7.0	3.5	–11.9
7		-x+1/2, y+1/2, -z+1/2	2	6.69	–4.6	–0.6	–13.8	12.2	–9.7
8		-x, -y, -z	1	7.28	7.9	–0.9	–10.4	10.0	4.8

[a] electrostatic (E_E), polarization (E_P), dispersion (E_D), and exchange-repulsion (E_R). $E_{tot} = k_E E_E + k_P E_P + k_D E_D + k_R E_R$, with k being scale factors. (11)

5.4. MNZ-GAL cocrystals

Table S13. Pairwise intermolecular interactions[a] seen in **MNZ-GAL form I°**.

No.	Interaction	Symop.	*n*	R Å	E_E	E_P	E_R kJ mol^{-1}	E_D	E_{tot}
1	2x O_{acid}–H···O_{acid}	-x, -y, -z	1	6.23	–120.9	–29.4	–20.1	163.6	–66.1
2	π···π	-	2	4.59	–68.9	–15.4	–39.2	105	–53.5
3	O_{acid}–H···O & C–H···O_{acid}	-	2	8.12	–96.8	–21.9	–13.4	132.1	–48.6
4	O–H···O	-	2	6.98	–44.3	–9.5	–17.9	63.0	–30.6
5	π···π	-	2	4.31	–2.8	–3.2	–47.4	33.9	–25.6
6	MNZ···MNZ	-x, y+1/2, -z+1/2	2	7.03	–13.8	–2.7	–14.5	9.6	–23.3
7	MNZ···GAL	-	2	7.78	–26.9	–5.1	–8.2	29.2	–21.4
8	MNZ···GAL	-	2	8.09	–12.8	–2.7	–6.7	11.0	–14.6
9	MNZ···MNZ	x, -y+1/2, z+1/2	2	7.43	–7.7	–2.8	–11.1	12.4	–12.2
10	MNZ···MNZ	-x, -y, -z	1	5.69	–5.6	–3.3	–17.2	18.2	–12.1
11	GAL···GAL	-x, y+1/2, -z+1/2	2	6.34	–0.1	–0.8	–15.2	4.6	–11.0
12	GAL···GAL	x, -y+1/2, z+1/2	2	7.53	–2.4	–0.5	–4.8	0.8	–6.6

13	MNZ···MNZ	-x, -y, -z	1	7.56	–2	–1.0	–3.6	0.2	–5.9
14	MNZ···GAL	-	2	8.67	–2.1	–0.4	–3.3	0.9	–4.8
15	MNZ···MNZ	-x, -y, -z	1	10.22	–1.9	–0.4	–2.5	0.7	–4.0
16	MNZ···GAL	-	2	8.82	0.5	–0.8	–4.4	1.9	–2.7
17	MNZ···MNZ	x, -y+1/2, z+1/2	2	8.78	0	–0.4	–1.7	0	–1.7
18	MNZ···MNZ	x, y, z	2	6.85	2.9	–0.5	–2.7	0.1	0.3

[a] electrostatic (E_E), polarization (E_P), dispersion (E_D), and exchange-repulsion (E_R). $E_{tot} = k_E E_E + k_P E_P + k_D E_D + k_R E_R$, with k being scale factors. (11)

Table S14. Pairwise intermolecular interactions[a] seen in **MNZ-GAL form II**.

No.	Interaction	Symop.	*n*	R Å	E_E	E_P	E_R kJ mol^{-1}	E_D	E_{tot}
1	2x O–H···O$_{acid}$	-x, -y, -z	1	6.11	–108.6	–24.6	–20.3	151.3	–57.3
2	O–H···O & π···π	-	2	4.24	–54.5	–13.3	–49.0	97.6	–49.8
3	O$_{acid}$–H···O & C–H···O$_{acid}$	-	2	8.04	–94.2	–21.2	–14.6	129	–48.3
4	O–H···O & C–H···O$_{acid}$	-	2	6.95	–72.3	–17.1	–19.3	101.3	–43.3
5	π···π	-	2	4.24	–8.9	–2.7	–35.9	18.2	–31.5
6	MNZ···MNZ	-x, y+1/2, -z+1/2	2	6.30	–12.6	–2.0	–8.8	1.6	–21.6
7	MNZ···GAL	-	2	7.27	–37.5	–8.5	–12.9	65.5	–16.8
8	MNZ···GAL	x, -y+1/2, z+1/2	2	7.47	–11.1	–1.3	–6.7	3.0	–16.7
9	MNZ···GAL	-	2	7.62	–13.3	–2.6	–10.7	15.8	–15.6
10	GAL···GAL	-x, y+1/2, -z+1/2	2	6.80	–4.2	–0.9	–11.5	2.5	–13.6
11	MNZ···MNZ	x, -y+1/2, z+1/2	2	7.47	–6.8	–1.6	–9.8	10	–10.7
12	MNZ···MNZ	-x, -y, -z	1	8.14	–0.3	–0.4	–10.1	6.7	–5.2
13	MNZ···MNZ	-x, -y, -z	1	9.32	–2.0	–0.6	–5.7	4.3	–4.8
14	MNZ···GAL	-	2	8.45	–0.5	–0.6	–4.1	1.7	–3.6
15	MNZ···MNZ	x, -y+1/2, z+1/2	2	8.85	–0.8	–0.1	–1.6	0	–2.3
16	MNZ···MNZ	x, y, z	2	7.1	3.6	–0.4	–2.1	0	1.8

[a] electrostatic (E_E), polarization (E_P), dispersion (E_D), and exchange-repulsion (E_R). $E_{tot} = k_E\,E_E + k_P\,E_P + k_D\,E_D + k_R\,E_R$, with k being scale factors. (11)

5.5. MNZ-GNT cocrystals

Table S15. Pairwise intermolecular interactions[a] seen in **MNZ-GNT form I.**

No.	Interaction	Symop.	*n*	R Å	E_E	E_P	E_R kJ mol^{-1}	E_D	E_{tot}
1	O–H···O & O–H···O	-x, -y, -z	1	5.92	–87.1	–19.9	–18.8	96.4	–63.6
2	O–H···N & C–H···O	-	2	7.45	–106.4	–31.9	–14.9	144.7	–59.7
3	π···π	-	2	3.82	–6.8	–2.6	–41.7	21.5	–32.2
4	O–H···O	-	2	7.28	–18.7	–5.1	–14.3	15.1	–26.7
5	C–H···N & C–H···O	-x, -y, -z	1	6.65	–17.7	–3.6	–13.8	11.2	–26.5
6	GNT···GNT	-x, -y, -z	1	4.67	–2.4	–1.9	–20.4	4.5	–18.9
7	MNZ···GNT	-	2	6.04	–6.8	–1.7	–21.4	15.2	–17.6
8	MNZ···MNZ	-x, -y, -z	1	5.00	–1.3	–3.1	–24.8	12.9	–17.2
9	MNZ···GNT	-	2	6.66	–6.4	–1.6	–10.8	4.5	–14.5
10	MNZ···GNT	-	2	8.07	–6.9	–2.2	–6.8	7.4	–10.3
11	MNZ···MNZ	-x, -y, -z	1	7.55	–3.1	–1.1	–7.5	4.3	–7.9
12	MNZ···GNT	-	2	7.95	–3.0	–0.5	–6.6	2.5	–7.8
13	GNT···GNT	-x, -y, -z	1	7.70	–2.1	–1.5	–4.9	0.4	–7.3
14	MNZ···MNZ	x, y, z	2	8.6	–1.6	–1.6	–5.4	2.8	–5.8
15	MNZ···GNT	-	2	8.75	–3.6	–1.0	–5.6	6.3	–5.5
16	MNZ···MNZ	-x, -y, -z	1	9.62	–1.6	–0.6	–5.3	2.8	–5.1
17	GNT···GNT	x, y, z	2	8.67	2.4	–0.3	–2.2	0	0.5

[a] electrostatic (E_E), polarization (E_P), dispersion (E_D), and exchange-repulsion (E_R). $E_{tot} = k_E\,E_E + k_P\,E_P + k_D\,E_D + k_R\,E_R$, with k being scale factors. (11)

Table S16. Pairwise intermolecular interactions[a] seen in **MNZ-GNT form II°.**

No.	Interaction	Symop.	$n/2$	R<br>Å	E_E	E_P	E_R<br>kJ mol^{-1}	E_D	E_{tot}
1	O–H···N & C–H···O	-	1	7.58	–105.2	–24.3	–14.0	134.6	–58.2
2	O–H···N & C–H···O	-	1	7.48	–116.4	–30.7	–14.9	170	–53.8
3	π···π	-	1	3.74	–18.4	–3.4	–47.2	31.8	–43.4
4	π···π & O–H···O	-	1	5.68	–60.9	–13.6	–32.1	98.5	–41.6
5	O–H···O	-	1	6.94	–47.7	–10.3	–20.6	62.6	–37.3
6	O–H···O (GTN)	-	1	6.43	–60.1	–13.1	–12.1	78.5	–35.3
7	O–H···O (MNZ)	-	1	7.49	–57.1	–12.0	–10.1	73.7	–32.5
8	π···π	-	1	3.71	–6	–2.8	–50.6	35.9	–30.3
9	NO_2···NO_2 (MNZ)	-x, -y, -z	0.5	5.03	–10.3	–5.2	–29.1	21.1	–27.1
10	2x C–H···O (MNZ)	-x, -y, -z	0.5	6.71	–23.8	–4.4	–16.6	29.1	–24.9
11	MNZ···MNZ	-x, -y, -z	0.5	6.54	–15.9	–3.5	–15.2	16.7	–22.3
12	MNZ···GNT	-	1	5.57	–9.4	–1.9	–25.6	21.1	–20.7
13	GNT···GNT	-x, -y, -z	0.5	4.71	–4.5	–1.6	–22.3	8.4	–20.1
14	GNT···GNT	-x, -y, -z	0.5	4.54	–0.6	–2.2	–24.9	8.4	–18.8
15	MNZ···MNZ	-x, -y, -z	0.5	4.92	–2.0	–3.4	–27.9	19.7	–16.8
16	MNZ···GNT	-	1	6.9	–9.1	–1.9	–12.9	12.2	–14.8
17	MNZ···GNT	-	1	8.07	–11.9	–2.6	–6.8	11	–13.6
18	MNZ···GNT	-	1	8.24	–14.3	–1.9	–6.2	15.2	–12.5
19	MNZ···GNT	-	1	8.52	–12.8	–1.4	–6.2	13	–11.9
20	MNZ···GNT	-	1	7.13	–5.1	–2.2	–15.1	13.8	–11.6
21	MNZ···GNT	-	1	7.69	–10.2	–2.6	–8.1	14.7	–10.6
22	MNZ···MNZ	-	1	8.14	–3.8	–1.0	–6.0	3.1	–8.2
23	MNZ···GNT	-	1	8.06	–2.1	–0.7	–5.7	1	–7.1
24	GNT···GNT	x, y, z	1	8.28	–3.8	–0.5	–2.9	0.4	–6.7
25	MNZ···GNT	-	1	7.87	–1.1	–0.8	–8.3	4.7	–6.2
26	MNZ···MNZ	-	1	8.44	0	–1.4	–5.1	2.4	–3.9
27	MNZ···GNT	-	1	7.35	1.7	–1.4	–8.2	4.1	–3.8
28	MNZ···MNZ	x, y, z	1	8.28	–1.0	–0.4	–2.3	0	–3.4
29	MNZ···MNZ	-x, -y, -z	0.5	9.99	–0.3	–0.5	–3.6	1	–3.2
30	MNZ···MNZ	-x, -y, -z	0.5	9.89	–0.3	–0.3	–3.7	1.1	–3.1
31	MNZ···MNZ	x, y, z	1	8.28	–0.3	–0.2	–1.9	0	–2.1
32	GNT···GNT	x, y, z	1	8.28	2.1	–0.5	–3.8	0.7	–1.1
33	GNT···GNT	-	1	7.71	3.3	–1.1	–4.7	0.4	–1.1

[a] electrostatic (E_E), polarization (E_P), dispersion (E_D), and exchange-repulsion (E_R). $E_{tot} = k_E E_E + k_P E_P + k_D E_D + k_R E_R$, with k being scale factors. (11)

5.6. Energy framework diagrams

A.

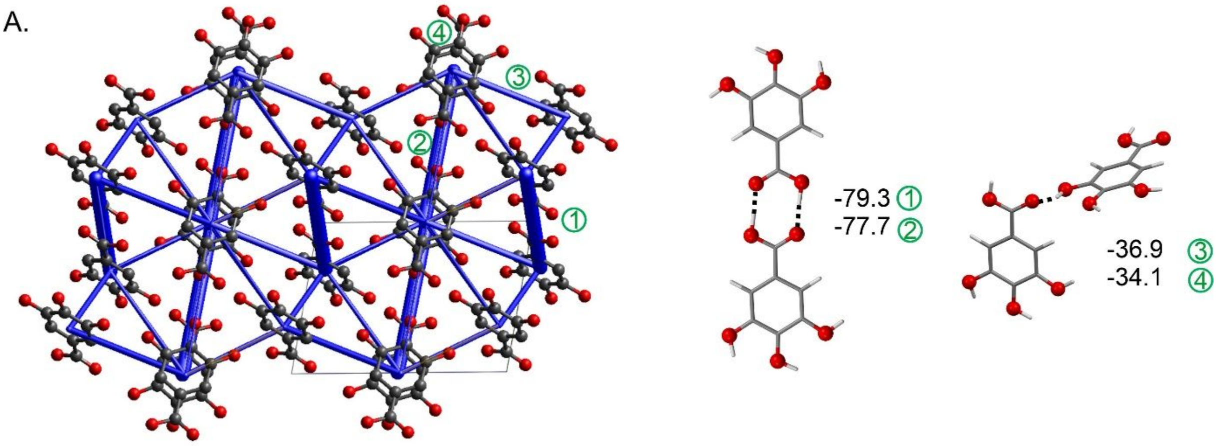

B.

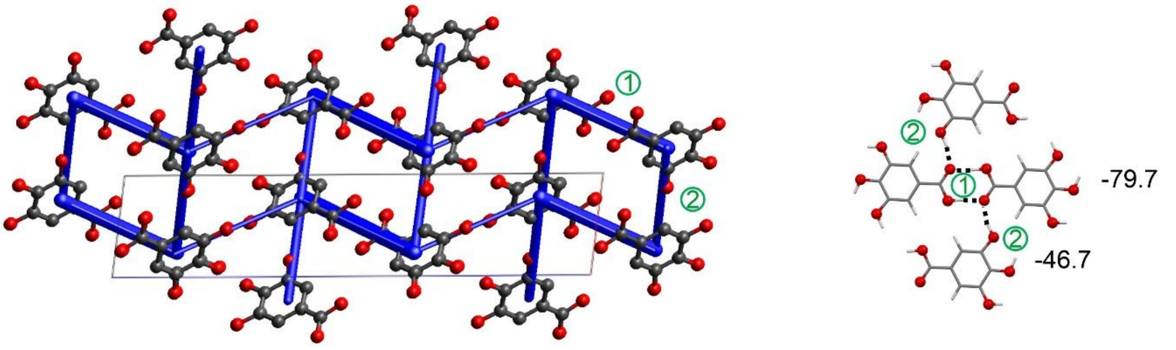

Figure S26. Energy framework diagram (total energy) for GAL form I (A.) and GAL form III (B.). The energy scale factor is 50. Stabilizing contacts are shown in blue and the thickness corresponds to the strength. Pairwise interaction energies <15 kJ mol^{-1} are omitted. Strongest pairwise interactions, incl. their energies in kJ mol^{-1}, are shown on the right.

Form I° Form II

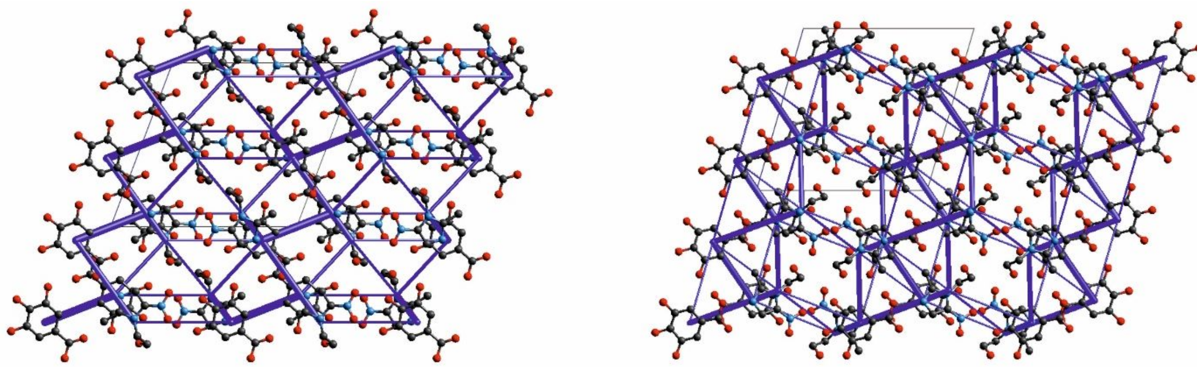

Figure S27. Energy framework diagram (total energy) for MNZ-GAL polymorphs. The energy scale factor is 50. Stabilizing contacts are shown in blue and the thickness corresponds to the strength. Pairwise interaction energies <15 kJ mol^{-1} are omitted.

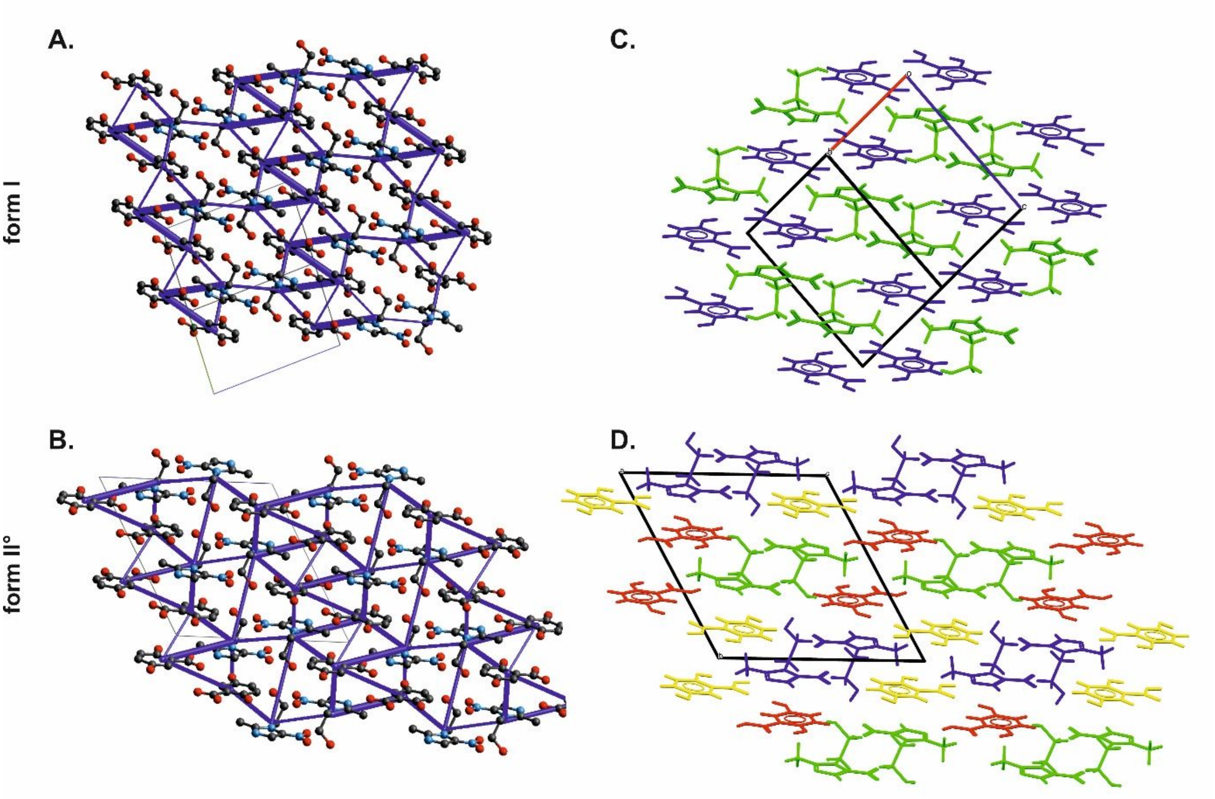

Figure S28. Energy framework diagram (total energy) for MNZ-GNT polymorphs (A. – B.). The energy scale factor is 50. Stabilizing contacts are shown in blue and the thickness corresponds to the strength. Pairwise interaction energies <15 kJ mol^{-1} are omitted. Packing diagrams of MNZ-GNT polymorphs (C. – D.). Crystallographically independent molecules are color coded. H-atoms are omitted for clarity.

6. Virtual cocrystal screening

6.1. Molecular complementarity (MC)

The results of the molecular complementarity search between metronidazole and the chosen coformers can be found in Table S17. Various conformations were considered, including those optimized in the gas phase and with flexible dihedrals fixed to experimentally observed values (constrained optimization).

In the case of adipic acid, only one out of seven conformations passed the M/L axis ratio criteria.

Table S17. Search for suitable coformers of metronidazole via molecular complementarity (MC).

Coformer	Overall	M/L axis ratio	S axis (Å)	S/L axis ratio	Dipole moment magnitude (Debye)	Fraction of N and O
malic acid	PASS	0.670-0.860	5.294-5.759	0.577-0.706	2.009-2.113	0.556
4-aminobenzoic acid	PASS	0.664	3.405	0.336	0.764	0.300
resorcinol	PASS	0.849-0.934	3.400-3.417	0.392-0.415	0.776-3.943	0.250
4-hydroxybenzoic acid	PASS	0.664-0.707	3.401-3.659	0.336-0.387	0.764-21.192	0.300
gallic acid	PASS	0.829-0.874	3.400-3.546	0.341-0.358	4.392	0.417
3-hydroxybenzoic acid	PASS	0.737-0.863	3.400-3.420	0.347-0.385	4.546-7.767	0.300
gentisic acid	PASS	0.811-0.862	3.401-3.427	0.361-0.382	0.803-3.041	0.364
salicylic acid	PASS	0.777-0.785	3.402	0.362-0.365	1.476-2.610	0.300
nicotinamide	PASS	0.702-0.710	3.708-4.929	0.395-0.528	0.691-2.399	0.333
adipic acid	PASS	0.444[a]-0.674	4.1455.682	0.331-0.487	0.185-0.709	0.400
benzoic acid	PASS	0.717-0.722	3.401-3.722	0.364-0.398	0.826-0.828	0.222
o-phthalic acid	PASS	0.921-0.988	3.892-5.272	0.446-0.569	0.525-2.572	0.333
ethyl gallate	PASS	0.694-0.669	4.168-4.703	0.339-0.383	5.956	0.357
3,5-dihydroxybenzoic acid	PASS	0.843-0.929	3.400-3.536	0.364-0.379	1.273-3.413	0.364
pyrogallol	PASS	0.917-0.993	3.400-3.828	0.392-0.481	2.863-5.764	0.333

[a] fail

6.2. Multi-component hydrogen-bond (MCHB) propensity screen

Table S18. Multi-component hydrogen-bond propensity screen results.

Rank	Component B	Multi-component score	Max interaction	Max A:B or B:A propensity	Max A:A propensity	Max B:B propensity
1	malic acid	0.22	A:B	0.84	0.62	0.52
2	4-aminobenzoic acid	0.18	B:A	0.8	0.59	0.62
3	3,5-dihydroxybenzoic acid	0.05	B:A	0.65	0.6	0.35
4	4-hydroxybenzoic acid	0.05	B:A	0.64	0.6	0.35
5	gallic acid	0.05	B:A	0.65	0.61	0.36
6	3-hydroxybenzoic acid	0.04	B:A	0.64	0.6	0.35
7	gentisic acid	0.04	B:A	0.64	0.6	0.34
8	salicylic acid	0.04	B:A	0.63	0.59	0.34
9	nicotinamide	-0.01	B:B	0.76	0.6	0.77
10	adipic acid	-0.02	A:A	0.61	0.62	0.37
11	benzoic acid	-0.03	A:A	0.59	0.62	0.38
12	o-phthalic acid	-0.03	A:A	0.6	0.63	0.37
13	ethyl gallate	-0.11	A:A	0.55	0.66	0.25
14	resorcinol	-0.12	A:A	0.53	0.65	0.1
15	pyrogallol	-0.13	A:A	0.53	0.66	0.1

6.3. Molecular electrostatic potential (MEP) maps

Using the identified local maxima (MEP_{max}) and minima (MEP_{min}), the H-bond donor parameter (α) and H-bond acceptor parameter (β) were calculated using equations (1) and (2), respectively, as described in previous studies (12).

$$\alpha = 0.0000162\ MEP_{max}^2 + 0.00962\ MEP_{max} \quad (1)$$

$$\beta = 0.000146\ MEP_{min}^2 - 0.00930\ MEP_{min} \quad (2)$$

The total pairing energy in the solid state was estimated by summing over a hierarchical listing of a specific number of complementary H-bond donor-acceptor sites, as shown in equation (3):

$$E = -\sum_{ij}\alpha_i\beta_j \quad (3)$$

To calculate the potential energy gain (ΔE_{MEP}, in kJ mol^{-1}) upon cocrystal formation, equation (4) was used:

$$\Delta E_{MEP} = E^{cc} - (E^{MNZ} + E^{CF}) \quad (4)$$

Here, CC, MNZ and CF represent the cocrystal, metronidazole, and coformer, respectively, and the energies correspond to the interaction energies calculated from the α and β values.

The following section provides the data used for deriving the *lowest* ΔE_{MEP} values (Table 3 of the manuscript).

6.3.1 MNZ–pyrogallol

Table S19. MEP calculations for **MNZ and pyrogallol**.

No.	α	kJ mol^{-1}	β	kJ/mol
1	4.3729	301.49	7.2392	–193.09
2	4.0713	285.73	5.5103	–165.02
3	3.7016	265.80	5.4471	–163.91
4	2.6706	206.08	5.0864	–157.50
5	2.0013	163.19	3.4375	–124.86
6	1.3440	116.75	1.4223	–71.86
7	1.2958	113.15	1.2192	–64.92
8	1.1960	105.56	1.1968	–64.13
9	1.1326	100.67	1.1679	–63.09
10	1.1254	100.11	0.9998	–56.82
11	1.0433	93.67	0.7542	–46.76

6.3.2 MNZ–ethyl gallate

Table S20. MEP calculations for **MNZ and ethyl gallate**.

No.	α	kJ mol^{-1}	β	kJ/mol
1	4.6439	315.31	6.0822	-174.73
2	4.0713	285.73	6.0688	-174.50
3	3.7748	269.80	5.5103	-165.02
4	3.0213	227.16	5.4471	-163.91
5	2.0013	163.19	5.3598	-162.38
6	1.3440	116.75	5.0864	-157.50
7	1.2958	113.15	3.4375	-124.86
8	1.1960	105.56	0.9151	-53.49
9	1.1326	100.67	0.9017	-52.95
10	1.1254	100.11	0.8188	-49.53
11	1.0369	93.17	0.7631	-47.15
12	0.9995	90.20	0.6252	-40.93
13	0.8772	80.32	0.6243	-40.88
14	0.8762	80.24	0.5183	-35.71
15	0.8750	80.14	0.5140	-35.49

6.3.3 MNZ–4-aminobenzoic acid

Table S21. MEP calculations for **MNZ and 4-aminobenzoic acid**.

No.	α	kJ mol^{-1}	β	kJ/mol
1	4.0713	285.73	5.5103	-165.02
2	3.3310	245.10	6.2139	-176.90
3	3.0441	228.51	5.4471	-163.91
4	3.0241	227.33	5.0864	-157.50
5	2.0013	163.19	3.4375	-124.86
6	1.3440	116.75	2.0504	-90.86
7	1.2958	113.15	2.0218	-90.06
8	1.1960	105.56	1.8893	-86.28
9	1.1326	100.67	0.9193	-53.66
10	1.1254	100.11	0.8762	-51.91
11	1.0369	93.17	0.8513	-50.88
12	0.8396	77.23	0.7665	-47.30
13	0.6593	62.05	0.7645	-47.21
14	0.6575	61.90	0.7252	-45.49

6.3.4 MNZ–3-hydroxybenzoic acid

Table S22. MEP calculations for **MNZ and 3-hydroxybenzoic acid**.

No.	α	kJ mol^{-1}	β	kJ/mol
1	4.2117	293.12	5.5103	-165.02
2	4.0713	285.73	5.4471	-163.91
3	3.7465	268.26	5.2304	-160.08
4	2.0013	163.19	5.0864	-157.50
5	1.3440	116.75	3.4375	-124.86
6	1.2958	113.15	3.0978	-117.26
7	1.2891	112.63	1.4315	-72.17
8	1.1960	105.56	1.4199	-71.78
9	1.1326	100.67	0.4688	-33.15
10	1.1254	100.11	0.4687	-33.15
11	1.0369	93.17	0.4372	-31.47
12	09497	86.20	0.4368	-31.44

6.3.5 MNZ–4-hydroxybenzoic acid

Table S23. MEP calculations for **MNZ and 4-hydroxybenzoic acid**.

No.	α	kJ mol^{-1}	β	kJ/mol
1	4.4044	303.11	5.5103	-165.02
2	4.0713	285.73	5.4471	-163.91
3	3.6277	261.73	5.2967	-161.26
4	2.0013	163.19	5.0864	-157.50
5	1.3440	116.75	3.4375	-124.86
6	1.2958	113.15	2.3620	-99.27
7	1.1960	105.56	1.5537	-76.12
8	1.1326	100.67	1.5471	-75.90
9	1.1254	100.11	0.4521	-32.27
10	1.0953	97.76	0.4500	-32.16
11	1.0369	93.17	0.3656	-27.47
12	0.9704	87.87	0.3653	-27.45
13	0.9037	82.48	0.3652	-27.45
14	0.6575	61.90	0.3646	-27.41
15	0.5713	54.40	0.3563	-26.93
16	0.5709	54.37	0.3545	-26.82

6.3.6 MNZ–3,5-dihydroxybenzoic acid

Table S24. MEP calculations for **MNZ and 3,5-dihydroxybenzoic acid**.

No.	α	kJ mol^{-1}	β	kJ/mol
1	4.4602	305.98	5.5103	-165.02
2	4.4217	304.00	5.4471	-163.91
3	4.0713	285.73	5.2943	-161.22
4	3.7165	266.62	5.0864	-157.50
5	2.0013	163.19	3.4375	-124.86
6	1.3440	116.75	2.9814	-114.56
7	1.2958	113.15	2.7009	-107.84
8	1.1960	105.56	1.6121	-77.95
9	1.1326	100.67	1.6110	-77.92
10	1.1254	100.11	0.5625	-37.92
11	1.0369	93.17	0.5620	-37.89
12	0.7422	69.11	0.4988	-34.72
13	0.6575	61.90	0.4985	-34.70
14	0.6557	61.74	0.2105	-17.71
15	0.6500	61.25	0.2078	-17.52

6.3.7 MNZ–adipic acid

Table S25. MEP calculations for **MNZ and adipic acid**.

No.	α	kJ mol^{-1}	β	kJ/mol
1	4.5485	310.48	5.7970	-169.94
2	4.5472	310.42	5.7939	-169.89
3	4.0713	285.73	5.5103	-165.02
4	2.0013	163.19	5.4471	-163.91
5	1.4010	120.98	5.0864	-157.50
6	1.3988	120.82	4.1771	-140.27
7	1.3440	116.75	4.1743	-140.21
8	1.2958	113.15	3.4375	-124.86

6.3.8 MNZ–benzoic acid

Table S26. MEP calculations for **MNZ and benzoic acid**.

No.	α	kJ mol^{-1}	β	kJ/mol
1	4.0713	285.73	5.5103	-165.02
2	3.7600	269.00	5.4471	-163.91
3	2.0013	163.19	5.0864	-157.50
4	1.3440	116.75	5.0099	-156.11
5	1.2958	113.15	3.4375	-124.86
6	1.2512	109.77	1.3649	-69.95
7	1.2077	106.45	1.3639	-69.92
8	1.1960	105.56	0.4548	-32.41
9	1.1659	103.24	0.4547	-32.41
10	1.1326	100.67	0.4255	-30.83
11	1.1254	100.11	0.4250	-30.81
12	1.0369	93.17	0.4247	-30.79

6.3.9 MNZ–gallic acid

Table S27. MEP calculations for **MNZ and gallic acid**.

No.	α	kJ mol^{-1}	β	kJ/mol
1	4.6485	315.54	5.5103	-165.02
2	4.0713	285.73	5.5066	-164.95
3	4.0551	284.87	5.5050	-164.92
4	3.7910	270.69	5.4471	-163.91
5	3.1657	235.60	5.0864	-157.50
6	2.0013	163.19	4.7614	-151.53
7	1.3440	116.75	3.4375	-124.86
8	1.2958	113.15	1.1603	-62.82
9	1.1960	105.56	1.1578	-62.72
10	1.1326	100.67	0.6778	-43.36
11	1.1254	100.11	0.6769	-43.32
12	1.0369	93.17	0.3699	-27.71
13	0.6575	61.90	0.3692	-27.68
14	0.6558	61.75	0.3602	-27.15
15	0.6537	61.57	0.3595	-27.12

6.3.10 MNZ–gentisic acid

Table S28. MEP calculations for **MNZ and gentisic acid**.

No.	α	kJ mol^{-1}	β	kJ/mol
1	4.1786	291.39	5.5103	-165.02
2	4.1582	290.31	5.4471	-163.91
3	4.0713	285.73	5.0864	-157.50
4	2.0013	163.19	3.7688	-131.94
5	1.3440	116.75	3.4375	-124.86
6	1.2958	113.15	3.1983	-119.55
7	1.2664	110.92	3.0065	-115.14
8	1.1960	105.56	1.0789	-59.82
9	1.1326	100.67	1.0767	-59.74
10	1.1307	100.52	0.3781	-28.19
11	1.1254	100.11	0.3770	-28.12
12	1.0369	93.17	0.3327	-25.54
13	0.9109	83.07	0.3326	-25.53

6.3.11 MNZ–malic acid

Table S29. MEP calculations for **MNZ and malic acid**.

No.	α	kJ mol^{-1}	β	kJ/mol
1	4.0713	285.73	8.1356	-206.35
2	4.0685	285.58	5.5103	-165.02
3	3.9957	281.71	5.4471	-163.91
4	3.7635	269.19	5.0864	-157.50
5	2.0013	163.19	4.4719	-146.04
6	1.3567	117.70	3.4375	-124.86
7	1.3509	117.27	2.7708	-109.55
8	1.3440	116.75	0.9672	-55.55
9	1.3394	116.41	0.9415	-54.54

6.3.12 MNZ–nicotinamide

Table S30. MEP calculations for **MNZ and nicotinamide**.

No.	α	kJ mol^{-1}	β	kJ/mol
1	4.0713	285.73	6.8633	-187.29
2	3.4433	251.46	5.9604	-172.70
3	2.9665	223.93	5.5103	-165.02
4	2.0013	163.19	5.4471	-163.91
5	1.6722	140.55	5.0864	-157.50
6	1.5271	130.19	3.4375	-124.86
7	1.3440	116.75	0.3901	-28.87
8	1.2958	113.15	0.0672	-6.55

6.3.13 MNZ–resorcinol

Table S31. MEP calculations for **MNZ and resorcinol**.

No.	α	kJ mol^{-1}	β	kJ/mol
1	4.0713	285.73	5.5103	-165.02
2	3.8281	272.70	5.4471	-163.91
3	3.8275	272.67	5.0864	-157.50
4	2.0013	163.19	3.6801	-130.08
5	1.3440	116.75	3.6761	-129.99
6	1.2958	113.15	3.6754	-129.98
7	1.1960	105.56	3.4375	-124.86
8	1.1326	100.67	1.1817	-63.59
9	1.1254	100.11	1.1816	-63.58
10	1.0996	98.10	1.1066	-60.85
11	1.0369	93.17	1.1058	-60.82
12	0.6575	61.90	1.1051	-60.80
13	0.6422	60.58	1.1048	-60.79

6.3.14 MNZ–salicylic acid

Table S32. MEP calculations for **MNZ and salicylic acid**.

No.	α	kJ mol^{-1}	β	kJ/mol
1	4.2212	293.09	5.5103	-165.02
2	4.0713	285.73	5.4471	-163.91
3	2.0013	163.19	5.0864	-157.50
4	1.3440	116.75	3.6414	-129.26
5	1.2958	113.15	3.4375	-124.86
6	1.2943	113.03	3.1424	-118.28
7	1.2376	108.74	0.9209	-53.72
8	1.1960	105.56	0.9174	-53.58
9	1.1587	102.69	0.5128	-35.43
10	1.1326	100.67	0.5127	-35.43
11	1.1254	100.11	0.4914	-34.33
12	1.0607	95.05	0.4908	-34.30

6.3.15 MNZ–*o*-phtalic acid

Table S33. MEP calculations for **MNZ and *o*-phtalic acid**.

No.	α	kJ mol^{-1}	β	kJ/mol
1	4.9516	330.63	8.2151	-207.49
2	4.0713	285.73	6.2012	-176.69
3	2.0013	163.19	5.5103	-165.02
4	1.6801	141.11	5.4471	-163.91
5	1.6655	140.08	5.0864	-157.50
6	1.6044	135.74	3.4375	-124.86
7	1.5675	133.11	1.7419	-81.93
8	1.5645	132.89	0.4415	-31.70

References

1. Ramkumar DHS, Kudchadker AP. Mixture properties of the water + .gamma.-butyrolactone + tetrahydrofuran system. Part 2. Viscosities and surface tensions of .gamma.-butyrolactone + water at 303.15-343.15 K and .gamma.-butyrolactone + tetrahydrofuran at 278.15-298.15 K. J Chem Eng Data. 1989 Oct 1;34(4):463–5.

2. Mumford SA, Phillips JWC. 19. The physical properties of some aliphatic compounds. J Chem Soc. 1950;75.

3. Wanchoo RK, Narayan J, Raina GK, Rattan VK. Excess properties of (2-Propanol + Ethyl Acetate or Benzene) Binary Liquid Mixture. Chem Eng Commun. 1989 Jul 25;81(1):145–56.

4. Ernst RC, Litkenhous EE, Spanyer JW. The Physical Properties of the Ternary System Acetone-n-Butyl Alcohol-Water. J Phys Chem. 1932 Mar 1;36(3):842–54.

5. Vazquez G, Alvarez E, Navaza JM. Surface Tension of Alcohol Water + Water from 20 to 50 .degree.C. J Chem Eng Data. 1995 May 1;40(3):611–4.

6. Nikitas P, Pappa-Louisi A. Thermodynamic and modelistic study of surface solutions: aqueous solutions containing 2-butanol. J Phys Chem. 1990 Jan 1;94(1):361–70.

7. Clark SJ, Segall MD, Pickard CJ, Hasnip PJ, Probert MIJ, Refson K, et al. First principles methods using CASTEP. Zeitschrift für Krist - Cryst Mater. 2005 May 1;220(5–6):567–70.

8. Perdew JP, Burke K, Ernzerhof M. Generalized Gradient Approximation Made Simple. Phys Rev Lett. 1996 Oct 28;77(18):3865–8.

9. Vanderbilt D. Soft self-consistent pseudopotentials in a generalized eigenvalue formalism. Phys Rev B. 1990 Apr 15;41(11):7892–5.

10. Tkatchenko A, Alfè D, Kim KS. First-Principles Modeling of Non-Covalent Interactions in Supramolecular Systems: The Role of Many-Body Effects. J Chem Theory Comput. 2012 Nov 13;8(11):4317–22.

11. Mackenzie CF, Spackman PR, Jayatilaka D, Spackman MA. CrystalExplorer model energies and energy frameworks: extension to metal coordination compounds, organic salts, solvates and open-shell systems. IUCrJ. 2017 Sep 1;4(5):575–87.

12. Musumeci D, Hunter CA, Prohens R, Scuderi S, McCabe JF. Virtual cocrystal screening. Chem Sci. 2011;2(5):883–90.
